# Correction: Structural validity of the Rosenberg self-esteem scale in patients with schizophrenia in Indonesia

**DOI:** 10.1371/journal.pone.0308836

**Published:** 2024-08-08

**Authors:** Muhammad Muslih, Min-Huey Chung

There are errors in the Funding statement. The correct Funding statement is as follows: This work was supported by grants from the University System of Taipei Joint Research Program (USTP-NTPU-TMU-113-02).
