# Supplementary material for: Structural validity of the Rosenberg self-esteem scale in patients with schizophrenia in Indonesia
Source: PLoS One. 2024 May 10;19(5):e0300184. doi: 10.1371/journal.pone.0300184 (PMC11086833; doi:10.1371/journal.pone.0300184)
Supplement: Attachment — Submitted filename: PONE-D-23-08354_R1.pdf [file pone.0300184.s002.pdf]

PLOS ONE

Construct Validity of the Rosenberg Self-Esteem Scale in patients with Schizophrenia in Indonesia

--Manuscript Draft--

**Manuscript Number:**	PONE-D-23-08354R1
**Article Type:**	Research Article
**Full Title:**	Construct Validity of the Rosenberg Self-Esteem Scale in patients with Schizophrenia in Indonesia
**Short Title:**	Construct Validity of the Rosenberg Self-Esteem Scale in patients with Schizophrenia in Indonesia
**Corresponding Author:**	Min-Huey Chung, Ph.D.<br>Taipei Medical University<br>Taipei, TAIWAN
**Keywords:**	self-esteem; validity; reliability, schizophrenia
**Abstract:**	Background: The Rosenberg self-esteem scale (RSES) is a commonly employed instrument for measuring self-esteem in the general population and those with mental illness. However, confirmatory factor analyses (CFA) to determine the factor structure and model fit of the RSES for schizophrenia patients are limited.<br>Objectives: We examined the validity and reliability evidence of the RSES by measuring confirmatory factors among patients with schizophrenia in Indonesia.<br>Methods: The sample comprised 260 participants. Over two weeks, 30 subjects were added to investigate test-retest reliability. We used internal consistency (Cronbach's alpha) and test-retest reliability to evaluate the reliability evidence. The validity evidence was based on a CFA to determine the model fit.<br>Results: The RSES had an alpha Cronbach coefficient of 0.75. While 0.89 and 0.88 for the positive and negative self-esteem subscale, respectively. Test-retest reliability yielded adequate results with an interclass correlation score ranging from 0.87 to 0.93. Four different models were analyzed in this study. Considering the single-factor model (Model 1a), the overall fit criteria were inadequate. However, after some modification indices, all fit criteria were significantly adequate (Model 1b). The adequacy of all fit standards remained satisfactory when the two-factor model (Model 2) and hierarchical model (Model 3) were applied.<br>Conclusions: The current investigation provided evidence supporting the construct validity and reliability of the RSES, indicating that the RSES can be considered a valid and reliable measurement. This finding suggests that the use of the RSES is beneficial and applicable in assessing levels of self-esteem in individuals diagnosed with schizophrenia in Indonesia.
**Order of Authors:**	Muhammad Muslih
	Min-Huey Chung, Ph.D.
**Response to Reviewers:**	Dear Editor and Reviewer<br><br>Manuscript ID:<br>Manuscript Title: “Construct Validity of the Rosenberg Self-Esteem Scale in Patients with Schizophrenia in Indonesia”<br><br>We sincerely thank the editor and all reviewers for their valuable suggestions and for giving us a great opportunity to revise our manuscript entitled “Construct Validity of the Rosenberg Self-Esteem Scale in Patients with Schizophrenia in Indonesia”. We have incorporated all the suggested changes into the manuscript and have highlighted the revised sections. Hereby, our responses and revisions are based on the editor and reviewer’s comments.<br><br>---<br><br>EDITORIAL COMMENTS:

Powered by Editorial Manager® and ProduXion Manager® from Aries Systems Corporation

Please respond to and address all the reviewers' recommendations before resubmission. The reviewers have pointed out that the introduction and methodology sections of the paper require improvement. Specifically, a sample of 260 subjects was used to perform an EFA and CFA. It is crucial to take their suggestions and make the necessary revisions to ensure that the paper complies with the quality standards of the instrument validation.
Response: Thank you for your correction. The necessary amendments and recommendations provided by the reviewers have been incorporated into our work.

REVIEWER #1 COMMENTS:

1.The submission studies the psychometric properties of an Indonesian version of the Rosenberg Self-Esteem Scale using a sample of inpatients diagnosed with schizophrenia. The Introduction is brief and does not justify the approach of the study sufficiently. Some sentences are confusing, e.g., in the first paragraph when the authors state that SE plays a role in the development of mental illness (i.e., precedent) but also state that this is because SE is a key outcome of mental illnesses (i.e., subsequent).
Response: Thank you for your corrections. We want to explain that self-esteem and mental illnesses have a bidirectional pattern. Low self-esteem makes people more vulnerable to the development of mental illnesses, and mental illness, in turn, lowers self-esteem. The paragraph has been revised to enhance the reader's comprehension of the author's intended message.
(Page 3)
There exists a reciprocal relationship between self-esteem and mental illnesses. A previous study found that self-esteem plays a pivotal role in developing diverse mental illnesses and social problems encompassing a range of internalizing issues, such as depression, suicidal tendencies, eating disorders, and anxiety, as well as externalizing problems, including violence and substance abuse [6]. Conversely, it has been hypothesized that mental illnesses can lead to low self-esteem as a significant consequence [7]. Consistent with prior research [8] low self-esteem has been found to heighten susceptibility to the onset of mental illness. In contrast, the existence of mental illness subsequently diminishes self-esteem. Eventually, individuals with mental illnesses are likely to have fluctuating self-esteem levels. Hence, self-esteem is considered a component of self-assessment, which influences mental health and vice versa.

2.Some results are confusing, e.g., using the same sample to conduct first an EFA and second a CFA, or describing the psychometric properties as validity. As a reader, I would expect reports of associations with other constructs typically related to SE and some sort of criterion validity. The authors also discuss the two-factor model without acknowledging previous studies on factor solutions in relation to wording. What is however an interesting finding is the extremely small association between the positive and the negative factor which is not in line with previous research. It might be beneficial to study this further.
Response: Thank you for your corrections. We have made the necessary revisions to our work. As a result, we have decided to exclusively focus on presenting confirmatory factor analysis (CFA) in order to demonstrate validity evidence of the RSES. (Statistical analysis; Page 7); (Results; Page 8); (Discussion; Page 9).

3.The sample is interesting and the psychometric test plausible, but I would expect such tests in such a sample as a first step of a paper on further relevant research questions. The psychometric tests alone do not seem like a substantial contribution to

Powered by Editorial Manager® and ProduXion Manager® from Aries Systems Corporation

	the literature.<br>Response: Thank you for your corrections. As previously stated, the RSES has been widely used as a scale to measure self-esteem in a variety of populations; but, to the best of our knowledge, there has been no previous research employing the RSES in schizophrenia patients, particularly in Indonesia. As a result of our hypothesis, the RSES psychometric test in our study can make a significant contribution. In addition, we revised and added more information in the last paragraph of the introduction section to describe our novelties.<br>(Page 4)<br>Multiple countries, including Indonesia, have implemented the RSES to measure the self-esteem of college students and the general population [26]. Interestingly, some previous studies have shown that self-esteem has been associated with schizophrenia [27-29]. For example, there was a significant correlation between a decrease in the intensity of adverse symptoms and an enhancement in self-esteem, and conversely, [30]. Unfortunately, the scale for people diagnosed with schizophrenia was not evaluated using psychometric testing in a prior study [26]. Additionally, they only focused on internal consistency and factor structure invariance.<br><br>REVIEWER #2 COMMENTS:<br><br>ABSTRACT:<br>1.The use of some terms when mentioning types of validity evidence, do not follow the current Standards. For example, the term "construct validity" should be replaced, according to the last versions of the Standards (AERA, APA, & NCME, 1999, 2014).<br>Response: Thank you for your corrections. We have corrected and changed it following the current standards.<br>(Page 2)<br>We examined the validity and reliability evidence of the RSES by measuring confirmatory factors among patients with schizophrenia in Indonesia<br><br>INTRODUCTION:<br>2.According to literature [6], self-esteem also plays a crucial role in developing various mental illnesses" -->Please determine the type and characteristics of mental illnesses.<br>Response: Thank you for your corrections. According to the intended literature, we amended and added information about the types and characteristics of mental illnesses.<br>(Page 3)<br>There exists a reciprocal relationship between self-esteem and mental illnesses. A previous study found that self-esteem plays a pivotal role in developing diverse mental illnesses and social problems encompassing a range of internalizing issues, such as depression, suicidal tendencies, eating disorders, and anxiety, as well as externalizing problems, including violence and substance abuse [6]. Conversely, it has been hypothesized that mental illnesses can lead to low self-esteem as a significant consequence [7]. Consistent with prior research [8] low self-esteem has been found to heighten susceptibility to the onset of mental illness. In contrast, the existence of mental illness subsequently diminishes self-esteem. Eventually, individuals with mental illnesses are likely to have fluctuating self-esteem levels. Hence, self-esteem is considered a component of self-assessment, which influences mental health and vice versa.<br><br>3.The use of some terms when mentioning types of validity evidence, do not follow the current Standards. For example, the term "construct validity" should be replaced, according to the last versions of the Standards (AERA, APA, & NCME, 1999, 2014).<br>Response: Thank you for your corrections. We have corrected and changed it following the current standards.<br>(Page 4)<br>Hence, evaluating the RSES in individuals diagnosed with schizophrenia is imperative to ascertain its validity and reliability<br><br>METHODS:<br>4.This study employed a cross-sectional design"--> Please determine the correct type of study. This is an instrumental questionnaire validation study, not a cross-sectional study.<br>Response: Thank you for your correction. We have corrected and replaced it in

accordance with your suggestions.
(Page 5)
This is an instrumental questionnaire validation study.

5.(e) provision of informed consent"-->The following element cannot be considered an inclusion criterion. This aspect should be considered in the ethics section.
Response: Thank you for your correction. We have corrected it in accordance with your suggestions.

6.The participants were informed before signing the written consent form that participation is voluntary and their confidentiality was carefully guarded. The purposes of the study, procedure for filling out questionnaires, potential risk or inconvenient, confidentiality issues and contact detail of the researcher were explained in the written consent forms and informs the participant directly. The researcher also gives an option to the participants to not participate in the study. The participants in this study was vulnerable group (psychiatric patients/ case with mental illness), therefore, additional protection was provided" --> Please review the flow of information in the methods section. From my humble point of view there are certain aspects that are not in the corresponding section (in this case ethics), besides repeating information.
Response: Thank you for your correction. This section has been moved to incorporate an ethics section.

7.The minimum number of participants recommended by Mundfrom, Shaw [29] was 20 times the number of variables, which in this case equaled 200 participants. In this study, data was missing from four questionnaires because they were not accurately completed, and 21 participants were excluded because their MMSE score was less than 24. Considering the response rate of 20%, the final sample comprised 260 participants"--> I seem to remember that the rule of 5 and 20 participants has long been out of use, it is calculated on the basis of expected communalities and items per factor, taking into account the work in statistical analysis under optimal conditions. I refer the authors to the citations of Ferrando-Piera et al. (2022) and Lloret-Segura et al. (2014).
Response: Thank you for your correction and suggestions. We have corrected it in accordance with your suggestions.
(Page 5)
The size of 200 participants required in this study is acceptable based on recommendations from prior studies [31, 32].

8.Sinclair, Blais [9] found that the dependability of the RSES was satisfactory, with a Cronbach's alpha of 0.91" -->Please determine internal consistency by scales or sub-dimensions, currently presenting internal consistency of total scale scores is a major methodological error. I refer the authors to the citation of Prinsen et al. (2018).
Response: Thank you for your correction. We have corrected and changed it in accordance with your suggestions.
(Page 6)
The validity and reliability of the RSES were assessed in prior studies, and the obtained results are presented herein. According to a previous study [33] it is suggested to ensure that the Cronbach alpha criteria for each sub-scale is ≥ 0.70. The Cronbach's alpha coefficients for the positive and negative self-esteem subscales were determined to be 0.96 and 0.98, respectively [19]. A prior study conducted on individuals who are native English speakers has also yielded Cronbach alpha coefficients of 0.87 and 0.75 for the subscales measuring positive and negative self-esteem, respectively [14]. Concerning the evaluation of validity evidence (construct validity), prior studies have demonstrated that the RSES yielded an excellent model fit [19, 34].

9.Franck, De Raedt [21] tested the construct validity by correlating the RSES score with the NEO Five-Factor Inventory subscales. In line with Tinakon and Nahathai [10], the RSES negatively correlated with the Thai Depression Inventory" --> Please determine the magnitude of the correlations in the different studies. The use of some terms when mentioning types of validity evidence, do not follow the current Standards.
Response: Thank you for your correction. We have corrected and changed it in accordance with your suggestions.
(Page 6)

Powered by Editorial Manager® and ProduXion Manager® from Aries Systems Corporation

Concerning the evaluation of validity evidence (construct validity), prior studies have demonstrated that the RSES yielded an excellent model fit [19, 34].

10.In the last phase, the complete Indonesian version of the RSES scale was administered to the study sample, and its validity and reliability were determined" --> The use of some terms when mentioning types of validity evidence, do not follow the current Standards.
Response: Thank you for your correction. We have corrected and changed it in accordance with your suggestions.
(Page 6-7)
In the final phase, the full Indonesian version of the RSES scale was administered to the study sample and tested the evidence of validity and reliability.

11.A Cronbach's alpha equal to or better than 0.70 demonstrates adequate internal consistency [33, 34]" --> From my humble point of view, the authors should have calculated the ordinal alpha. The rest of the statistics in categorical response scales underestimate reliability. I refer the authors to the citation of Zumbo et al. (2007).
Response: Thank you for your correction and suggestion. In this section, we adhere to the same content as our initial composition. To the best of our knowledge, ordinal alpha is merely a measure of hypothetical reliability and does not reflect the actual reliability of the test. Herewith is an article pertaining to the misconceptions and the limited usefulness of ordinal alpha.
Reference: Chalmers, R. P. (2018). On misconceptions and the limited usefulness of ordinal alpha. Educational and Psychological Measurement, 78(6), 1056-1071.

12.We carried out an EFA (exploratory factor analysis) to examine the construct validity of the scale using a principal component analysis [36, 37] with varimax rotation [38]" -->I seem to remember that the use of orthogonal rotations in highly correlated multidimensional constructs may deform the underlying structure of test item scores. I refer the authors to the citations of Ferrando-Piera et al. (2022), Lloret-Segura et al. (2014) and Prinsen et al. (2018).
Response: Thank you for your correction. After conducting a comprehensive assessment of the relevant literature, we have made the necessary revisions to our work. As a result, we have decided to exclusively focus on presenting confirmatory factor analysis (CFA) in order to demonstrate validity evidence of RSES. Therefore, EFA analysis is not performed in our revision.

13.to determine the number of components, an eigenvalue greater than 1.00 was utilized [43, 44]" --> According to the latest standards it is more correct to use parallel analysis to determine the number of factors. I refer the authors to the citations of Ferrando-Piera et al. (2022) and Lloret-Segura et al. (2014). Adequacy of EFA (KMO; Barteltt's test of sphericity and determinant) should come before "parallel analysis" or "number of factors" to determine the number of factors to be retained, not after as now appears in several places of the text.
Response: Thank you for your correction. After conducting a comprehensive assessment of the relevant literature, we have made the necessary revisions to our work. As a result, we have decided to exclusively focus on presenting confirmatory factor analysis (CFA) in order to demonstrate validity evidence of RSES. Therefore, EFA analysis is not performed in our revision.

14.I do not observe the criteria used to retain or discard items in the EFA; the authors should provide scientific evidence in this regard.
Response: Thank you for your correction. After conducting a comprehensive assessment of the relevant literature, we have made the necessary revisions to our work. As a result, we have decided to exclusively focus on presenting confirmatory factor analysis (CFA) in order to demonstrate validity evidence of RSES. Therefore, EFA analysis is not performed in our revision.

15.The authors do not make it clear whether they randomly segmented the sample into two halves to perform the CFA and EFA.
Response: Thank you for your correction. The same sample was utilized for both exploratory factor analysis (EFA) and confirmatory factor analysis (CFA), without employing random allocation. After conducting a comprehensive assessment of the relevant literature, we have made the necessary revisions to our work. As a result, we

Powered by Editorial Manager® and ProduXion Manager® from Aries Systems Corporation

have decided to exclusively focus on presenting confirmatory factor analysis (CFA) in order to demonstrate validity evidence of RSES.

16.Why do the authors perform an EFA and a CFA with the same sample, what does this contribute to the study?
Response: Thank you for your correction. The same sample was utilized for both exploratory factor analysis (EFA) and confirmatory factor analysis (CFA), without employing random allocation. After conducting a comprehensive assessment of the relevant literature, we have made the necessary revisions to our work. As a result, we have decided to exclusively focus on presenting confirmatory factor analysis (CFA) in order to demonstrate validity evidence of RSES.

17.I still do not understand why with a sample of 260 participants and without segmenting it the authors perform an EFA and a CFA, taking into consideration that a sample of 130 to perform the different statistical methods would be unstable due to the small size. I refer the authors to the citations of Ferrando-Piera et al. (2022) and Lloret-Segura et al. (2014).
Response: Thank you for your correction. We have made the necessary revisions to our work, and the EFA analysis is not performed in our revision. We are focused on presenting confirmatory factor analysis (CFA) only.

18.From my humble point of view, it would be more interesting to perform only a CFA with several adjustments: a) congeneric model, b) correlated error model if the modification rates are greater than or equal to 35000 and c) a second order model to assess the existence of a single underlying construct.
Response: Thank you for your correction and suggestion. Our manuscript has been revised, and the results of several CFA models are presented in the result section (Page 8), discussion section (Page 10-11), Table 4, and Figures 1 - 4.

19."convergent validity"--> The use of some terms when mentioning types of validity evidence, do not follow the current Standards. I refer the authors to the citation of Prinsen et al. (2018).
Response: Thank you for your correction. The paper has been amended to emphasize the construct validity of the Rosenberg Self-Esteem Scale (RSES). Consequently, we will now proceed to provide the results of the Confirmatory Factor Analysis (CFA) to elucidate the aforementioned construct.

RESULTS:
20.There are variables that have not been described in the methodology section. The authors should include a section where readers can see all the sociodemographic variables collected. The authors should write the variables more clearly, specifically if they are continuous or categorical variables. In the case of categorical variables, they should include the response options in parentheses. The authors should describe the evaluation instruments more clearly. I recommend writing it with a hyphen, the name of the instrument, and its psychometric properties.
Response: Thank you for your correction. We have corrected and changed it in accordance with your suggestions.
(Page 5)
The study incorporates many socio-demographic data, including age as a continuous variable. The remaining variables as categorical variables, namely gender (1=male; 2=female), marital status (1=single; 2=married; 3=divorced, or widowed), employment status (1=employed; 2=unemployed), source of income (1=personal income; 2=family support; 3=personal and family support), education (1=elementary; 2=junior; 3=high school; 4=university/ college), previous hospitalization (1=yes; 2=no), and onset of illness (1=<1 year; 2=1-5 years; 3=>5 years).

(Page 6)
The validity and reliability of the RSES were assessed in prior studies, and the obtained results are presented herein.

21.The authors should provide normative and performance data for the scale items (ceiling and floor effects). Based on the results found, if floor and ceiling effects are found, the authors should provide percentiles to facilitate the interpretation of the scores. Authors should explore differences between men and women and provide

Powered by Editorial Manager® and ProduXion Manager® from Aries Systems Corporation

effect sizes. However, t-test for examining differences in direct score between ratings of men and women should be conducted after demonstrating measurement invariance across this variable, at least at the factor loadings level (weak o metric invariance) and item thresholds/intercepts level (strong or scalar invariance), to ensure that direct scores are operating in the same way and, therefore, are meaningfully comparable.
Response: Thank you for your correction. We have corrected and changed it in accordance with your suggestions.
(Page 9)
Floor and ceiling effect was found at 5.80% - 54.20%, respectively (Table 3).

DISCUSSION:
22.In addition, evidence of the construct validity of the RSES was demonstrated through EFA and CFA, contrary to previous studies conducted in Indonesia" --> Is it appropriate to do this with a total sample of 260 participants?
Response: Thank you for your correction. Considering the reviewers' advice regarding the sample size to be used in EFA and CFA, we updated our work to simply report the CFA results.

23.In line with this finding, a previous investigation found satisfactory levels of internal consistency, as measured by a Cronbach's alpha coefficient that ranged from 0.81 to 0.91 [9, 10, 21, 25]" --> What are the implications of obtaining an internal consistency greater than 0.90?
Response: Thank you for your correction. The reliability coefficient is bounded within 0 – 1, where values closer to 1 indicate better measurement precision (i.e., less sampling and measurement error). To the best of our knowledge, achieving an internal consistency value greater than 0.90 is indicative of a favorable level of reliability and consistency in the scale's ability to measure the intended construct.

24.Another study indicated that a two-factor model considerably increases the fit compared to a one-factor model [58]" -->The following statement supports my humble opinion of performing a CFA with several adjustments, specifically congeneric and a second-order model.
Response: Thank you for your correction and suggestion. The manuscript has been enhanced through the inclusion of Confirmatory Factor Analysis (CFA) outcomes derived from several different models.

(Page 10)
We examine four models in our study: the single-factor (Model 1a), the single-factor with correlated error (Model 1b), the two-factor (Model 2), and the hierarchical model (Model 3). The results of Model 1a indicate that there is inadequate evidence to support the adequacy of a single factor. A notable distinction was observed while implementing the correlated error in Model 1b, as it demonstrates an enhancement in the adequate fit criteria. This adjustment was carried out on the negative self-esteem factor, a negatively worded item. This finding aligns with some prior studies that have examined the presence of method effects related to negative items on the RSES [55-57]. The model with a single factor with correlated error yielded the best model fit.

(Page 10-11)
Surprisingly, adequate fit indexes were also obtained in Models 2 and 3. The two-factor and hierarchical models exhibit comparable model fit in their respective analyses. Based on the findings mentioned earlier, it is suggested that the Rosenberg Self-Esteem Scale (RSES) can be conceptualized as comprising two distinct constructs, specifically positive self-esteem and negative self-esteem. Similar findings were also demonstrated in a previous study that a two-factor model was deemed to have an adequate model fit [16, 19, 58, 59]. In summary, our evidence indicates that the RSES scale construct has an acceptable model fit, which means that the RSES is suitable for individuals with schizophrenia in Indonesia. In short, the evidence from our study shows that the RSES scale construct fits well, which means that the RSES is a valid scale to use with people with schizophrenia in Indonesia.

(Page 11)
Based on the rationale mentioned earlier, our study's results indicate that the RSES is a unidimensional measure of overall self-esteem, as initially proposed by Rosenberg

Powered by Editorial Manager® and ProduXion Manager® from Aries Systems Corporation

(9). As previously established by various studies, RSES was also shown to assess two separate variables (positive and negative self-esteem) [16, 19, 59]. According to a prior study [60] it has been firmly indicated that the RSES is uni-dimensional. However, the influence of wording impacts on scale items may result in or contribute to bi-dimensionality. Acknowledging the necessity of reassessing the utilization of the RSES and its theoretical foundations in administering the scale to target populations is imperative.
25.Authors should include in the limitations section that the majority of the sample on which we can interpret the item scores are men.
Response: Thank you for your corrections and suggestions. Hereby we present some of the limitations and ways to overcome them in our study.
(Page 11)
The present study also has limitations. Because our study sample included only patients with schizophrenia, these findings cannot be extrapolated to populations with other mental illnesses. Moreover, a significant majority of our study's participants are men, leading to a more prominent interpretation of item scores among this group. Future studies should aim to expand the participant pool by including individuals diagnosed with a diverse range of mental illnesses while also ensuring a balanced representation of both genders. In addition to the limitations mentioned above, it is essential to note that our study produced favorable outcomes regarding the validity and reliability of evidence. Hence establishing the RSES as a valid and reliable questionnaire appropriate for implementation within the tested sample group.

CONCLUSIONS:
26.The authors' statements in the conclusions section should be based on the statistical recommendations provided. Are we sure that the structure has not been influenced by orthogonal rotation?
Response: Thank you for your correction. We have corrected and changed it in accordance with your suggestions. However, in relation to the text revision, our attention was directed towards the results of the confirmatory factor analysis (CFA). Consequently, our validity assessment demonstrated that the model aligns with the RSES as indicated by these findings.

ADDITIONAL COMMENTS:
27.Table 1. Demographic Characteristics of the Study (n = 260)" --> The sum of the percentages exceeds or does not reach 100%, the authors should check the sum of the tables
Response: Thank you for your correction. We have corrected and replaced it in accordance with your suggestions. (Table 1)

REVIEWER #3 COMMENTS:
1.It is a ver straightforward article in which the construct validity of the Rosenberg Self-Esteem Scale for schizophrenia patients in Indonesia is assessed. I have to admit that I was not expecting the length of this article and I received it with great pleasure. Nowadays articles this long are not that easy to find. It is very easy to read and very straightforward as well, which is one of its strong points, in my humble opinion.
Response: We express our gratitude for your kind attention.

2.However, the introduction section is lacking some depth. The authors state that "self-esteem plays a crucial role in developing various mental illnesses" (page 3, line 7). This affirmation makes me think that self-esteem triggers some mental disorders. But then, following the previous sentence: "This is because low self-esteem has been postulated to be one of the primary outcomes of mental illness". This part needs some clarification. Also, in the last paragraph of the introduction (page 4, line 2), the authors state: "Additionally, some previous studies have shown that self-esteem has been associated with schizophrenia". In which way? In general, although I can guess the intention of the authors in making a short paper, I think the introduction section would benefit from deeper research.
Response: Thank you for your corrections. In paragraph 1, we want to explain that self-esteem and mental illnesses have a bidirectional pattern. Low self-esteem makes people more vulnerable to the development of mental illnesses, and mental illness, in turn, lowers self-esteem. The paragraph has been revised to enhance the reader's comprehension of the author's intended message.

	(Page 3)<br>There exists a reciprocal relationship between self-esteem and mental illnesses. A previous study found that self-esteem plays a pivotal role in developing diverse mental illnesses and social problems encompassing a range of internalizing issues, such as depression, suicidal tendencies, eating disorders, and anxiety, as well as externalizing problems, including violence and substance abuse [6]. Conversely, it has been hypothesized that mental illnesses can lead to low self-esteem as a significant consequence [7]. Consistent with prior research [8] low self-esteem has been found to heighten susceptibility to the onset of mental illness. In contrast, the existence of mental illness subsequently diminishes self-esteem. Eventually, individuals with mental illnesses are likely to have fluctuating self-esteem levels. Hence, self-esteem is considered a component of self-assessment, which influences mental health and vice versa.<br><br>Response: We have added an explanation of the relationship between self-esteem and schizophrenia in the last paragraph of the introduction. The paragraph has been revised to enhance the reader's comprehension of the author's intended message.<br>(Page 4)<br>Multiple countries, including Indonesia, have implemented the RSES to measure the self-esteem of college students and the general population [26]. Interestingly, some previous studies have shown that self-esteem has been associated with schizophrenia [27-29]. For example, there was a significant correlation between a decrease in the intensity of adverse symptoms and an enhancement in self-esteem, and conversely, [30]. Unfortunately, the scale for people diagnosed with schizophrenia was not evaluated using psychometric testing in a prior study [26]. Additionally, they only focused on internal consistency and factor structure invariance.<br><br>3.The methods, results, and discussion sections are great. They are short and straightforward, like the rest of the paper.<br>Response: We express our gratitude for your kind attention.<br><br>4.My main concern is the fact that references do not follow the APA format. Please revise it carefully.<br>Response: Thank you for your corrections. To the best of our knowledge, according to the journal writing guidelines, compiling a bibliography follows the Vancouver format.<br>https://journals.plos.org/plosone/s/submission-guidelines
**Additional Information:**	
**Question**	**Response**
**Financial Disclosure**<br><br>Enter a financial disclosure statement that describes the sources of funding for the work included in this submission. Review the submission guidelines for detailed requirements. View published research articles from *PLOS ONE* for specific examples.<br><br>This statement is required for submission and **will appear in the published article** if the submission is accepted. Please make sure it is accurate.	The authors did not received any funding for this study.

**Unfunded studies**<br>Enter: *The author(s) received no specific funding for this work.*<br><br>**Funded studies**<br>Enter a statement with the following details:<br>• Initials of the authors who received each award<br>• Grant numbers awarded to each author<br>• The full name of each funder<br>• URL of each funder website<br>• Did the sponsors or funders play any role in the study design, data collection and analysis, decision to publish, or preparation of the manuscript?<br>• **NO** - Include this sentence at the end of your statement: *The funders had no role in study design, data collection and analysis, decision to publish, or preparation of the manuscript.*<br>• **YES** - Specify the role(s) played.<br><br>* typeset	
**Competing Interests**<br><br>Use the instructions below to enter a competing interest statement for this submission. On behalf of all authors, disclose any competing interests that could be perceived to bias this work—acknowledging all financial support and any other relevant financial or non-financial competing interests.<br><br>This statement is required for submission and **will appear in the published article** if the submission is accepted. Please make sure it is accurate and that any funding sources listed in your Funding Information later in the submission form are also declared in your Financial Disclosure statement.<br><br>View published research articles from *PLOS ONE* for specific examples.	The authors declare that they have no conflicts of interest.

**NO authors have competing interests**<br><br>Enter: *The authors have declared that no competing interests exist.*<br><br>**Authors with competing interests**<br><br>Enter competing interest details beginning with this statement:<br><br>*I have read the journal's policy and the authors of this manuscript have the following competing interests: [insert competing interests here]*<br><br>* typeset	
**Ethics Statement**<br><br>Enter an ethics statement for this submission. This statement is required if the study involved:<br><br>• Human participants<br>• Human specimens or tissue<br>• Vertebrate animals or cephalopods<br>• Vertebrate embryos or tissues<br>• Field research<br><br>Write "N/A" if the submission does not require an ethics statement.<br><br>General guidance is provided below. Consult the submission guidelines for detailed instructions. **Make sure that all information entered here is included in the Methods section of the manuscript.**	The Ethics Research Committee approved this research of the University of Muhammadiyah Malang on July 19, 2018 (approval number: E.5.a/239/KEPK-UMM/VII/2018).The participants were informed before signing the written consent form that participation is voluntary and their confidentiality was carefully guarded. The purposes of the study, procedure for filling out questionnaires, potential risk or inconvenient, confidentiality issues and contact detail of the researcher were explained in the written consent forms and informs the participant directly. The researcher also gives an option to the participants to not participate in the study. The participants in this study was vulnerable group (psychiatric patients/ case with mental illness), therefore, additional protection was provided. The researcher was involved with the nurse and must be under supervision by the nurse in charge during data collection.

Powered by Editorial Manager® and ProduXion Manager® from Aries Systems Corporation

**Format for specific study types**<br><br>**Human Subject Research (involving human participants and/or tissue)**<br>• Give the name of the institutional review board or ethics committee that approved the study<br>• Include the approval number and/or a statement indicating approval of this research<br>• Indicate the form of consent obtained (written/oral) or the reason that consent was not obtained (e.g. the data were analyzed anonymously)<br><br>**Animal Research (involving vertebrate animals, embryos or tissues)**<br>• Provide the name of the Institutional Animal Care and Use Committee (IACUC) or other relevant ethics board that reviewed the study protocol, and indicate whether they approved this research or granted a formal waiver of ethical approval<br>• Include an approval number if one was obtained<br>• If the study involved *non-human primates*, add *additional details* about animal welfare and steps taken to ameliorate suffering<br>• If anesthesia, euthanasia, or any kind of animal sacrifice is part of the study, include briefly which substances and/or methods were applied<br><br>**Field Research**<br><br>Include the following details if this study involves the collection of plant, animal, or other materials from a natural setting:<br>• Field permit number<br>• Name of the institution or relevant body that granted permission	
**Data Availability**<br><br>Authors are required to make all data underlying the findings described fully available, without restriction, and from the time of publication. PLOS allows rare exceptions to address legal and ethical concerns. See the PLOS Data Policy and FAQ for detailed information.	Yes - all data are fully available without restriction

Powered by Editorial Manager® and ProduXion Manager® from Aries Systems Corporation

A Data Availability Statement describing where the data can be found is required at submission. Your answers to this question constitute the Data Availability Statement and **will be published in the article**, if accepted.<br><br>**Important:** Stating 'data available on request from the author' is not sufficient. If your data are only available upon request, select 'No' for the first question and explain your exceptional situation in the text box.<br><br>Do the authors confirm that all data underlying the findings described in their manuscript are fully available without restriction?	
**Describe where the data may be found in full sentences. If you are copying our sample text, replace any instances of XXX with the appropriate details.**<br><br>• If the data are **held or will be held in a public repository**, include URLs, accession numbers or DOIs. If this information will only be available after acceptance, indicate this by ticking the box below. For example: *All XXX files are available from the XXX database (accession number(s) XXX, XXX.).*<br>• If the data are all contained **within the manuscript and/or Supporting Information files**, enter the following: *All relevant data are within the manuscript and its Supporting Information files.*<br>• If neither of these applies but you are able to provide **details of access elsewhere**, with or without limitations, please do so. For example:<br><br>*Data cannot be shared publicly because of [XXX]. Data are available from the XXX Institutional Data Access / Ethics Committee (contact via XXX) for researchers who meet the criteria for access to confidential data.*<br><br>*The data underlying the results presented in the study are available from (include the name of the third party*	All relevant data are within the paper and its S1 Dataset files. Please inform the authors if data are being used.

and contact information or URL).<br>• This text is appropriate if the data are owned by a third party and authors do not have permission to share the data.<br><br>* typeset	
Additional data availability information:	

Manuscript
Click here to access/download;Manuscript;Manuscript20231004_clean.docx

Construct Validity of the Rosenberg Self-Esteem Scale in Patients with Schizophrenia in Indonesia

Muhammad Muslih [a, b], Min-Huey Chung [a, c*]

[a] School of Nursing, College of Nursing, Taipei Medical University, Taiwan

[b] School of Nursing, Faculty of Health Science, Universitas Muhammadiyah Malang, Indonesia

[c] Department of Nursing, Shuang Ho Hospital, Taipei Medical University, New Taipei City, Taiwan

Corresponding Author:

Min-Huey Chung, PhD, RN, FAAN

School of Nursing, College of Nursing, Taipei Medical University

No. 250, Wu-Xing Street, Taipei, Taiwan, 110, ROC.

Email address: minhuey300@tmu.edu.tw

Abstract

Background: The Rosenberg self-esteem scale (RSES) is a commonly employed instrument for measuring self-esteem in the general population and those with mental illness. However, confirmatory factor analyses (CFA) to determine the factor structure and model fit of the RSES for schizophrenia patients are limited.

Objectives: We examined the validity and reliability evidence of the RSES by measuring confirmatory factors among patients with schizophrenia in Indonesia.

Methods: The sample comprised 260 participants. Over two weeks, 30 subjects were added to investigate test-retest reliability. We used internal consistency (Cronbach's alpha) and test-retest reliability to evaluate the reliability evidence. The validity evidence was based on a CFA to determine the model fit.

Results: The RSES had an alpha Cronbach coefficient of 0.75. While 0.89 and 0.88 for the positive and negative self-esteem subscale, respectively. Test-retest reliability yielded adequate results with an interclass correlation score ranging from 0.87 to 0.93. Four different models were analyzed in this study. Considering the single-factor model (Model 1a), the overall fit criteria were inadequate. However, after some modification indices, all fit criteria were significantly adequate (Model 1b). The adequacy of all fit standards remained satisfactory when the two-factor model (Model 2) and hierarchical model (Model 3) were applied.

Conclusions: The current investigation provided evidence supporting the construct validity and reliability of the RSES, indicating that the RSES can be considered a valid and reliable measurement. This finding suggests that the use of the RSES is beneficial and applicable in assessing levels of self-esteem in individuals diagnosed with schizophrenia in Indonesia.

Keywords: self-esteem; validity; reliability, schizophrenia

1. Introduction

Self-esteem is an overall individual evaluation or appraisal of the self [1] and how a person thinks of themselves. Self-esteem is "the degree to which a person values, approves of, or likes himself or herself." [2]. Self-esteem is a crucial component of mental health and general psychological well-being. It influences an individual's achievements and successes, social interactions, and ability to cope with environmental stressors [3, 4]. Individuals with high self-esteem believe they possess many positive qualities and attitudes toward themselves [5]. In summary, self-esteem is a pivotal psychological construct that controls several facets of an individual's existence, encompassing mental well-being, accomplishments, interpersonal engagements, and coping abilities.

There exists a reciprocal relationship between self-esteem and mental illnesses. A previous study found that self-esteem plays a pivotal role in developing diverse mental illnesses and social problems encompassing a range of internalizing issues, such as depression, suicidal tendencies, eating disorders, and anxiety, as well as externalizing problems, including violence and substance abuse [6]. Conversely, it has been hypothesized that mental illnesses can lead to low self-esteem as a significant consequence [7]. Consistent with prior research [8] low self-esteem has been found to heighten susceptibility to the onset of mental illness. In contrast, the existence of mental illness subsequently diminishes self-esteem. Eventually, individuals with mental illnesses are likely to have fluctuating self-esteem levels. Hence, self-esteem is considered a component of self-assessment, which influences mental health and vice versa.

The Rosenberg Self-Esteem Scale (RSES), which was developed by Rosenberg (9), is one of the most extensively used instruments for measuring self-esteem globally [10-14]. Researchers often use the RSES to measure self-esteem in the clinical population [15]. In a prior study, the factor structure of the RSES was examined using psychometric tests, and it focused on adolescents [16], as they were the original target population of this scale. It has also been tested in adults [17] and the general population [18]. Other studies have tested the RSES in specific people, such as ex-prisoners [19], drug

users [20], and single mothers [21]. Accordingly, the RSES applies to participants from various samples or populations. Nevertheless, there is no available evidence supporting the utilization of RSES among individuals diagnosed with schizophrenia in Indonesia.

The RSES has been translated and adapted into a variety of languages, including German [18], Dutch [22], Estonian [23], French [24], Portuguese [25], Japanese [14], and Thai [11]. It has been adapted across 53 nations with distinct ethnic groups and cultures [26]. This finding indicates that the RSES is widely used to measure self-esteem. Supporting this idea, Sinclair, Blais (10) suggest that the popularity of the RSES can be attributed to its brevity and simplicity, as it comprises only ten questions that can be completed within a short timeframe of 1 to 2 minutes.

Multiple countries, including Indonesia, have implemented the RSES to measure the self-esteem of college students and the general population [26]. Interestingly, some previous studies have shown that self-esteem has been associated with schizophrenia [27-29]. For example, there was a significant correlation between a decrease in the intensity of adverse symptoms and an enhancement in self-esteem, and conversely, [30]. Unfortunately, the scale for people diagnosed with schizophrenia was not evaluated using psychometric testing in a prior study [26]. Additionally, they only focused on internal consistency and factor structure invariance.

Nevertheless, there is no available evidence supporting the utilization of RSES among individuals diagnosed with schizophrenia in Indonesia. Hence, evaluating the RSES in individuals diagnosed with schizophrenia is imperative to ascertain its validity and reliability. This study aimed to assess the construct validity of the RSES as a measure for patients with schizophrenia in Indonesia through confirmatory factor analyses (CFA).

2. Methods

2.1 Participants

This is an instrumental questionnaire validation study. Two psychiatric hospitals and one psychiatric rehabilitation center in East Java, Indonesia, were visited to obtain the required data. We distributed the questionnaire from August 2018 to February 2019. Participants were recruited using the convenience sampling technique. The following requirements had to be met for someone to be included: (a) they had been diagnosed with schizophrenia; (b) aged ≥ 20 years; (c) hospitalized in a psychiatric ward; and (d) able to speak, read, and write Indonesian. The Mini-Mental State Examination (MMSE) was utilized to screen out participants with cognitive impairment (i.e., MMSE scores < 24).

The size of 200 participants required in this study is acceptable based on recommendations from prior studies [31, 32]. In this study, data was missing from four questionnaires because they were not accurately completed, and 21 participants were excluded because their MMSE score was less than 24. Considering the response rate of 20%, the final sample comprised 260 participants. In addition, we recruited an additional 30 individuals to investigate test-retest reliability over two weeks.

2.2 Instruments

The study incorporates many socio-demographic data, including age as a continuous variable. The remaining variables as categorical variables, namely gender (1=male; 2=female), marital status (1=single; 2=married; 3=divorced, or widowed), employment status (1=employed; 2=unemployed), source of income (1=personal income; 2=family support; 3=personal and family support), education (1=elementary; 2=junior; 3=high school; 4=university/ college), previous hospitalization (1=yes; 2=no), and onset of illness (1=<1 year; 2=1-5 years; 3=>5 years).

The RSES is not licensed and is available for public use. Information about the scale can easily be gathered, and permission to use this resource can be sought at https://socy.umd.edu/about-us/rosenberg-self-esteem-scale. The scale consists of 10 items evaluated on a 4-point Likert scale from

1 (strongly disagree) to 4 (strongly agree). Scores vary from 10 to 40, with higher scores indicating a more positive self-esteem appraisal. To measure the reverse score, five questions are worded positively (items 1, 3, 4, 7, and 10), while five are worded negatively (items 2, 5, 6, 8, and 9).

The validity and reliability of the RSES were assessed in prior studies, and the obtained results are presented herein. According to a previous study [33] it is suggested to ensure that the Cronbach alpha criteria for each sub-scale is ≥ 0.70. The Cronbach's alpha coefficients for the positive and negative self-esteem subscales were determined to be 0.96 and 0.98, respectively [19]. A prior study conducted on individuals who are native English speakers has also yielded Cronbach alpha coefficients of 0.87 and 0.75 for the subscales measuring positive and negative self-esteem, respectively [14]. Concerning the evaluation of validity evidence (construct validity), prior studies have demonstrated that the RSES yielded an excellent model fit [19, 34].

2.3 Translation Procedure

Due to their specificity and straightforwardness, we adhered to the parameters suggested by Sousa and Rojjanasrirat (35). Initially, the original questionnaire was translated into Indonesian by two translators, a psychiatrist and a professional translator whose native language was Indonesian, with the author's approval. Both were bilingual and English-proficient. Second, we compared the two translated versions and created a new draft by combining the terminology and phrases supplied by the two translators in the previous step. Thirdly, the information was back-translated by two more independent translators with the same credentials and qualities as the first translators. Fourth, we compared the original questionnaire with the two back-translations of the questionnaire from the third phase. Considering the distance and time variations, we communicated with all four translators by email at this stage. Fifth, 30 volunteers were selected from a psychiatric hospital to evaluate the clarity of the questionnaire's instructions, items, and response format. In addition, we asked two professionals (a psychiatrist and a psychologist) for revisions and ideas. In the final phase, the full Indonesian version

of the RSES scale was administered to the study sample and tested the evidence of validity and reliability.

2.4 Statistical Analysis

This study utilized SPSS and AMOS version 23.0 software (IBM; Armonk, New York, USA). All statistical significance was indicated by a p-value < 0.05.

2.4.1 Descriptive analysis

Descriptive statistics were used to present the demographic characteristics of the study. The proportion of participants who obtained minimum and maximum scores is defined as floor and ceiling effect, respectively. Continuous variables are presented as means and standard deviations, whereas categorical variables are expressed as frequencies and percentages. The quantitative characteristics of the RSES were computed as mean, standard deviation (SD), skewness, and kurtosis. A skewness value between −1 and 1 was considered adequate [36]. The degree of vertical spread in the mean distribution corresponded to the kurtosis. Morgan and Griego (37) said that normality was assumed if the kurtosis value was less than 2.5 times the standard error.

2.4.2 Reliability evidence

Internal consistency (Cronbach's alpha) and the interclass correlation coefficient (ICC) were used to evaluate the RSES' test-retest reliability. A Cronbach's alpha equal to or better than 0.70 demonstrates adequate internal consistency [38, 39]. The ICC score between 0.75 and 0.90 indicated satisfactory reliability and consistency between two-time measurements, and a score greater than 0.90 revealed excellent reliability [40].

2.4.3 Validity evidence

A CFA was carried out to assess how well the RSES model fits the data. The following fit indices were utilized during the evaluation process: X^2/df, the comparative fit index (CFI), incremental fit index (IFI), the Tucker–Lewis index (TLI), goodness-of-fit index (GFI), adjusted goodness-of-fit index (AGFI), the standardized root mean square residual (SRMR), and the root mean square error of

approximation (RMSEA). A chi-square with a degree of freedom ratio of less than 5.0 indicated that the model was a good fit [41]. An acceptable model fit was characterized by a GFI greater than 0.80 [42] and an AGFI of 0.80 to 0.90 [43-47]. When the CFI, IFI, and TLI values were all greater than 0.90 [48], the SRMR value was less than 0.08 [48, 49], and the RMSEA value was less than 0.10 [42], the model fit was deemed to be satisfactory. Akaike Information Criterion (AIC) and Bayesian Information Criterion (BIC) were used to evaluate the alternative model, with lower AIC and BIC values indicating the best model fit [50, 51].

The composite reliability (CR) measurement and average variance extracted (AVE) are integral components of the comprehensive construct validity assessment. The values of CR were more significant than 0.70 [52], and AVE was more critical than 0.50 [53].

2.5 Ethical Approval

The Ethics Research Committee approved this research of the University of Muhammadiyah Malang on July 19, 2018 (approval number: E.5.a/239/KEPK- UMM/VII/2018).

3. Results

Table 1 presents the clinical demographics of the study. The mean age of the 260 participants was 38.13 (SD = 9.56). Most participants were men (169, 65%) and single (139, 53.5%). Most were unemployed (173, 66.5%) and received financial support from their families (196, 75.4%). For 127 participants (48.8%), the highest educational level was senior high school. In total, 220 participants (84.6%) had previously been hospitalized, and the illness duration to onset was >5 years for 142 participants (54.6%).

Table 2 displays the descriptive statistics, inter-item correlation, and item-total correlation of the RSES. Item 8 and item 3 received the highest and lowest mean scores of 2.57 (SD = 0.87) and 1.92 (SD = 0.81), respectively. The skewness score ranged from 0.39 to 0.90 for the total RSES items, and

the kurtosis score ranged from 0.74 to 0.75. The item-total correlation varied from 0.47 to 0.6. Floor and ceiling effect was found at 5.80% - 54.20%, respectively (Table 3).

3.1 Reliability evidence

The RSES had an alpha coefficient of 0.75, according to Cronbach's method. The results of Cronbach's alpha, which measures internal consistency, came in at 0.89 and 0.88 for each subscale (factor), indicating acceptable reliability. Test-retest reliability exhibited satisfactory results, with an ICC between 0.87 and 0.93 (Table 3).

3.2 Validity evidence

As presented in Table 3, the CR was calculated for positive and negative factors, and the values were 0.92 and 0.91, respectively. The AVE values were 0.69 and 0.68, and the square roots of the AVE were 0.83 and 0.82, indicating that each measured variable was significant. The goodness of indices for all alternative models is shown in Table 4. Considering the single-factor model (Model 1a), the overall fit criteria were inadequate (See Figure 1). However, after some modification indices (See Figure 2), all fit criteria were significantly adequate (Model 1b). The adequacy of all fit criteria remained satisfactory when the two-factor model (Model 2) and hierarchical model (Model 3) were applied (See Figures 3 and 4).

4. Discussion

This study aimed to establish the construct validity of the RSES. We included patients with schizophrenia in our study, which was not the case in the study by Schmitt and Allik (26). Our sample size was adequate to perform factor analysis. In addition, evidence of the construct validity of the RSES was demonstrated through CFA, contrary to previous studies conducted in Indonesia.

The total score on the Indonesian version of the RSES had a Cronbach's alpha coefficient of 0.75. In contrast, the positive self-esteem subscale had a coefficient of 0.89, and the negative self-esteem subscale had a coefficient of 0.88. In line with this finding, a previous investigation found satisfactory

levels of internal consistency, as measured by a Cronbach's alpha coefficient that ranged from 0.81 to 0.91 [10, 11, 22, 26]. Our results were consistent with a previous study by [54], conducted in individuals with severe mental illnesses, not specific only to patients with schizophrenia and reported strong internal consistency. This study also showed that the RSES has good internal consistency, especially for a particular group of people (those with schizophrenia).

In addition, we looked at the test-retest reliability of the RSES. The ICC values generated were 0.93 for the positive self-esteem subscale and 0.87 for the negative self-esteem subscale. The ICC results were adequate, indicating the stability of each factor of the RSES. Additionally, it was observed that there was a high correlation coefficient between the test-retest reliability and Cronbach's alpha. In conclusion, the findings of this study provide evidence supporting the robust reliability of the RSES as a reliable instrument for assessing self-esteem in individuals diagnosed with schizophrenia in the Indonesian context.

We examine four models in our study: the single-factor (Model 1a), the single-factor with correlated error (Model 1b), the two-factor (Model 2), and the hierarchical model (Model 3). The results of Model 1a indicate that there is inadequate evidence to support the adequacy of a single factor. A notable distinction was observed while implementing the correlated error in Model 1b, as it demonstrates an enhancement in the adequate fit criteria. This adjustment was carried out on the negative self-esteem factor, a negatively worded item. This finding aligns with some prior studies that have examined the presence of method effects related to negative items on the RSES [55-57]. The model with a single factor with correlated error yielded the best model fit.

Surprisingly, adequate fit indexes were also obtained in Models 2 and 3. The two-factor and hierarchical models exhibit comparable model fit in their respective analyses. Based on the findings mentioned earlier, it is suggested that the Rosenberg Self-Esteem Scale (RSES) can be conceptualized as comprising two distinct constructs, specifically positive self-esteem and negative self-esteem. Similar findings were also demonstrated in a previous study that a two-factor model was deemed to

have an adequate model fit [16, 19, 58, 59]. In summary, our evidence indicates that the RSES scale construct has an acceptable model fit, which means that the RSES is suitable for individuals with schizophrenia in Indonesia. In short, the evidence from our study shows that the RSES scale construct fits well, which means that the RSES is a valid scale to use with people with schizophrenia in Indonesia.

Based on the rationale mentioned earlier, our study's results indicate that the RSES is a unidimensional measure of overall self-esteem, as initially proposed by Rosenberg (9). As previously established by various studies, RSES was also shown to assess two separate variables (positive and negative self-esteem) [16, 19, 59]. According to a prior study [60] it has been firmly indicated that the RSES is uni-dimensional. However, the influence of wording impacts on scale items may result in or contribute to bi-dimensionality. Acknowledging the necessity of reassessing the utilization of the RSES and its theoretical foundations in administering the scale to target populations is imperative.

The present study also has limitations. Because our study sample included only patients with schizophrenia, these findings cannot be extrapolated to populations with other mental illnesses. Moreover, a significant majority of our study's participants are men, leading to a more prominent interpretation of item scores among this group. Future studies should aim to expand the participant pool by including individuals diagnosed with a diverse range of mental illnesses while also ensuring a balanced representation of both genders. In addition to the limitations mentioned above, it is essential to note that our study produced favorable outcomes regarding the validity and reliability of evidence. Hence establishing the RSES as a valid and reliable questionnaire appropriate for implementation within the tested sample group.

5. Conclusion

The current investigation provided evidence supporting the construct validity and reliability of the RSES, indicating that the RSES can be considered a valid and reliable measurement. This finding suggests that the use of the RSES is beneficial and applicable in assessing levels of self-esteem in individuals diagnosed with schizophrenia in Indonesia. Nevertheless, further research is needed to understand better the characteristics of the method factors for different populations.

Supporting information

S1 Dataset

Data availability

All relevant data are within the paper and its S1 Dataset files. Please inform the authors if data are being used.

Conflict of Interest

The authors declare that they have no conflicts of interest.

Funding

The authors did not receive any funding for this study.

Acknowledgment

We would like to acknowledge Wallace Academic Editing Company for editing this manuscript.

Authors' Contributions

M.M collected, analyzed, and interpreted the data and drafted the manuscript. M.H.C studied and analyzed the data and co-drafted the manuscript.

References

1. Cast AD, Burke PJ. A theory of self-esteem. Social forces. 2002;80(3):1041-68.
2. Blascovich J, Tomaka J. Measures of self-esteem. Measures of personality and social psychological attitudes. 1991;1:115-60.

3. Battle J. Promoting self-esteem, achievement and well being: An effective instructional curriculum for all levels: James Battle and Associates; 1994.

4. Huang C, Dong N. Factor structures of the Rosenberg self-esteem scale. European Journal of Psychological Assessment. 2012.

5. Bonfine N. Stigma self concept and stigma resistance among individuals with mental illness [Disertation]. Ann Arbor: Kent State University; 2013.

6. Mann M, Hosman CM, Schaalma HP, de Vries NK. Self-esteem in a broad-spectrum approach for mental health promotion. Health Educ Res. 2004;19(4):357-72. Epub 2004/06/17. doi: 10.1093/her/cyg041. PubMed PMID: 15199011.

7. Link BG, Struening EL, Neese-Todd S, Asmussen S, Phelan JC. Stigma as a barrier to recovery: The consequences of stigma for the self-esteem of people with mental illnesses. Psychiatric services. 2001;52(12):1621-6.

8. Silverstone PH, Salsali M. Low self-esteem and psychiatric patients: Part I – The relationship between low self-esteem and psychiatric diagnosis. Annals of General Hospital Psychiatry. 2003;2(1):2. doi: 10.1186/1475-2832-2-2.

9. Rosenberg M. Society and the Adolescent Self-Image. Revised edition. Middletown, CT: Wesleyan University Press; 1989.

10. Sinclair SJ, Blais MA, Gansler DA, Sandberg E, Bistis K, LoCicero A. Psychometric properties of the Rosenberg Self-Esteem Scale: Overall and across demographic groups living within the United States. Evaluation & the health professions. 2010;33(1):56-80.

11. Tinakon W, Nahathai W. A comparison of reliability and construct validity between the original and revised versions of the Rosenberg Self-Esteem Scale. Psychiatry investigation. 2012;9(1):54.

12. Martin CR, Thompson DR, Chan DS. An examination of the psychometric properties of the Rosenberg Self-Esteem Scale (RSES) in Chinese acute coronary syndrome (ACS) patients. Psychology, health & medicine. 2006;11(4):507-21.

13. Martín-Albo J, Núñez JL, Navarro JG, Grijalvo F. The Rosenberg Self-Esteem Scale: translation and validation in university students. The Spanish journal of psychology. 2007;10(2):458-67.

14. Mimura C, Griffiths P. A Japanese version of the Rosenberg Self-Esteem Scale: Translation and equivalence assessment. Journal of Psychosomatic Research. 2007;62(5):589-94.

15. Salerno L, Ingoglia S, Coco GL. Competing factor structures of the Rosenberg Self-Esteem Scale (RSES) and its measurement invariance across clinical and non-clinical samples. Personality and Individual Differences. 2017;113:13-9.

16. Marsh HW, Scalas LF, Nagengast B. Longitudinal tests of competing factor structures for the Rosenberg Self-Esteem Scale: traits, ephemeral artifacts, and stable response styles. Psychological assessment. 2010;22(2):366.

17. Gana K, Saada Y, Bailly N, Joulain M, Hervé C, Alaphilippe D. Longitudinal factorial invariance of the Rosenberg Self-Esteem Scale: Determining the nature of method effects due to item wording. Journal of Research in Personality. 2013;47(4):406-16.

18. Roth M, Decker O, Herzberg PY, Brähler E. Dimensionality and norms of the Rosenberg Self-Esteem Scale in a German general population sample. European Journal of Psychological Assessment. 2008;24(3):190-7.

19. Boduszek D, Hyland P, Dhingra K, Mallett J. The factor structure and composite reliability of the Rosenberg Self-Esteem Scale among ex-prisoners. Personality and individual differences. 2013;55(8):877-81.

20. Wang J, Siegal HA, Falck RS, Carlson RG. Factorial structure of Rosenberg's Self-Esteem Scale among crack-cocaine drug users. Structural Equation Modeling. 2001;8(2):275-86.

21. Hatcher J, Hall LA. Psychometric properties of the Rosenberg self-esteem scale in African American single mothers. Issues in Mental Health Nursing. 2009;30(2):70-7.

22. Franck E, De Raedt R, Barbez C, Rosseel Y. Psychometric properties of the Dutch Rosenberg self-esteem scale. Psychologica Belgica. 2008;48(1):25-35.

23. Pullmann H, Allik J. The Rosenberg Self-Esteem Scale: its dimensionality, stability and personality correlates in Estonian. Personality and Individual differences. 2000;28(4):701-15.

24. Vallieres EF, Vallerand RJ. Traduction et validation canadienne- française de l'échelle de l'estime de soi de Rosenberg. International journal of psychology. 1990;25(2):305-16.

25. Vasconcelos-Raposo J, Fernandes HM, Teixeira CM, Bertelli R. Factorial validity and invariance of the Rosenberg Self-Esteem Scale among Portuguese youngsters. Social Indicators Research. 2012;105(3):483-98.

26. Schmitt DP, Allik J. Simultaneous administration of the Rosenberg Self-Esteem Scale in 53 nations: exploring the universal and culture-specific features of global self-esteem. Journal of personality and social psychology. 2005;89(4):623.

27. Moritz S, Veckenstedt R, Randjbar S, Vitzthum F, Karow A, Lincoln TM. Course and determinants of self- esteem in people diagnosed with schizophrenia during psychiatric treatment. Psychosis. 2010;2(2):144-53.

28. Haug E, Øie MG, Andreassen OA, Bratlien U, Romm KL, Møller P, et al. The association between anomalous self-experiences, self-esteem and depressive symptoms in first episode schizophrenia. Frontiers in human neuroscience. 2016;10:557.

29. Kim EY, Jang MH. The Mediating Effects of Self-Esteem and Resilience on the Relationship Between Internalized Stigma and Quality of Life in People with Schizophrenia. Asian Nursing Research. 2019;13(4):257-63. doi: https://doi.org/10.1016/j.anr.2019.09.004.

30. Jones RM, Hansen L, Moskvina V, Kingdon D, Turkington D. The relationship between self-esteem and psychotic symptoms in schizophrenia: A longitudinal study. Psychosis. 2010;2(3):218-26.

31. Ferrando Piera PJ, Lorenzo Seva U, Hernández Dorado A, Muñiz Fernández J. Decálogo para el Análisis Factorial de los Ítems de un Test. Psicothema. 2022.

32. Lloret-Segura S, Ferreres-Traver A, Hernandez-Baeza A, Tomas-Marco I. Exploratory item factor analysis: A practical guide revised and updated. Anales de Psicología. 2014;30(3):1151-69.

33. Prinsen CA, Mokkink LB, Bouter LM, Alonso J, Patrick DL, De Vet HC, et al. COSMIN guideline for systematic reviews of patient-reported outcome measures. Quality of life research. 2018;27:1147-57.

34. Xu ML, Leung SO. Effects of varying numbers of Likert scale points on factor structure of the Rosenberg Self- Esteem Scale. Asian Journal of Social Psychology. 2018;21(3):119-28.

35. Sousa VD, Rojjanasrirat W. Translation, adaptation and validation of instruments or scales for use in cross-cultural health care research: a clear and user-friendly guideline. Journal of

Evaluation Clinical Practice. 2011;17:268-74. Epub 2010/09/30. doi: 10.1111/j.1365-2753.2010.01434.x. PubMed PMID: 20874835.

36. Indrayan A, Malhotra RK. Medical biostatistics: Chapman and Hall/CRC; 2017.

37. Morgan GA, Griego OV. Easy use and interpretation of SPSS for Windows: Answering research questions with statistics: Psychology Press; 1998.

38. Polit DF, Beck CT. Essentials of nursing research: Appraising evidence for nursing practice: Lippincott Williams & Wilkins; 2010.

39. Tavakol M, Dennick R. Making sense of Cronbach's alpha. International journal of medical education. 2011;2:53.

40. Koo TK, Li MY. A guideline of selecting and reporting intraclass correlation coefficients for reliability research. Journal of chiropractic medicine. 2016;15(2):155-63.

41. Marsh HW, Hocevar D. Application of confirmatory factor analysis to the study of self-concept: First-and higher order factor models and their invariance across groups. Psychological bulletin. 1985;97(3):562.

42. Meyers LS, Gamst G, Guarino AJ. Applied Multivariate Research: Design and Interpretation: SAGE Publications; 2006.

43. Bentler PM. Comparative fit indexes in structural models. Psychological bulletin. 1990;107(2):238.

44. Marsh HW, Balla JR, McDonald RP. Goodness-of-fit indexes in confirmatory factor analysis: The effect of sample size. Psychological bulletin. 1988;103(3):391.

45. Cole DA. Utility of confirmatory factor analysis in test validation research. Journal of consulting and clinical psychology. 1987;55(4):584.

46. Floyd FJ, Widaman KF. Factor analysis in the development and refinement of clinical assessment instruments. Psychological assessment. 1995;7(3):286.

47. Greenspoon PJ, Saklofske DH. Confirmatory factor analysis of the multidimensional students' life satisfaction scale. Personality and Individual Differences. 1998;25(5):965-71.

48. Hu Lt, Bentler PM. Cutoff criteria for fit indexes in covariance structure analysis: Conventional criteria versus new alternatives. Structural equation modeling: a multidisciplinary journal. 1999;6(1):1-55.

49. Hoyle RH. Structural equation modeling: Concepts, issues, and applications: Sage; 1995.

50. Akaike H. A new look at the statistical model identification. IEEE transactions on automatic control. 1974;19(6):716-23.

51. Schwarz G. Estimating the dimension of a model. The annals of statistics. 1978:461-4.

52. Grewal R, Cote JA, Baumgartner H. Multicollinearity and measurement error in structural equation models: Implications for theory testing. Marketing science. 2004;23(4):519-29.

53. Hair J, Anderson R, Black B, Babin B. Multivariate Data Analysis: Pearson Education; 2016.

54. Torrey WC, Mueser KT, McHugo GH, Drake RE. Self-esteem as an outcome measure in studies of vocational rehabilitation for adults with severe mental illness. Psychiatric Services. 2000;51(2):229-33.

55. Mullen SP, Gothe NP, McAuley E. Evaluation of the factor structure of the Rosenberg Self-Esteem Scale in older adults. Personality and individual differences. 2013;54(2):153-7.

56. Aluja A, Rolland J-P, García LF, Rossier J. Dimensionality of the Rosenberg Self-Esteem Scale and its relationships with the three-and the five-factor personality models. Journal of Personality Assessment. 2007;88(2):246-9.

57. Makhubela M, Mashegoane S. Psychological validation of the Rosenberg Self-Esteem Scale (RSES) in South Africa: Method effects and dimensionality in black African and white university students. Journal of Psychology in Africa. 2017;27(3):277-81.

58. Quilty LC, Oakman JM, Risko E. Correlates of the Rosenberg self-esteem scale method effects. Structural Equation Modeling. 2006;13(1):99-117.

59. Boduszek D, Shevlin M, Mallett J, Hyland P, O'Kane D. Dimensionality and construct validity of the Rosenberg self- esteem scale within a sample of recidivistic prisoners. Journal of Criminal Psychology. 2012;2(1):19-25.

60. Reise SP, Kim DS, Mansolf M, Widaman KF. Is the bifactor model a better model or is it just better at modeling implausible responses? Application of iteratively reweighted least squares to the Rosenberg Self-Esteem Scale. Multivariate behavioral research. 2016;51(6):818-38.

Table 1. Demographic Characteristics of the Study (*n* = 260)

**Characteristics**	**Participants (*n* = 260)**	
	**Mean (SD)**	**n (%)**
Age	38.13 (9.56)	
Gender		
Male		169 (65.00)
Female		91 (35.50)
Marital status		
Single		139 (53.46)
Married		81 (31.15)
Divorce or widowed		40 (15.39)
Employment status		
Employed		87 (33.50)
Unemployed		173 (66.50)
Source of income		
Personal income		30 (11.50)
Family support		196 (75.40)
Personal and family		34 (13.10)
Education		
Elementary school		63 (24.23)
Junior high school		44 (16.92)
Senior high school		127 (48.85)
University/college		26 (10.00)
Previous hospitalization		
Yes		220 (84.60)
No		40 (15.40)
Onset of illness		
<1 year		75 (28.85)
1–5 years		43 (16.54)
>5 years		142 (54.61)

*missing data = 4; **MMSE score < 24 = 21
SD = standard deviation; MMSE = Mini-Mental State Examination.

Table 2. Descriptive Statistics, Interitem, and Item-Total Correlation of the RSES Items (n = 260)

Items		Interitem correlation (n = 260)									
		1	2	3	4	5	6	7	8	9	10
RSES 1	On the whole, I am satisfied with myself.	1									
RSES 2	At times, I think I am no good at all.	−0.11	1								
RSES 3	I feel that I have a number of good qualities.	0.56	−0.12	1							
RSES 4	I am able to do things as well as most other people.	0.51	−0.04	0.63	1						
RSES 5	I feel I do not have much to be proud of.	−0.03	0.48	−0.13	−0.11	1					
RSES 6	I certainly feel useless at times.	−0.00	0.62	−0.04	−0.01	0.48	1				
RSES 7	I feel that I'm a person of worth, at least on an equal plane with others.	0.52	−0.01	0.64	0.86	−0.07	0.06	1			
RSES 8	I wish I could have more respect for myself.	−0.19	0.71	−0.22	−0.15	0.43	0.50	−0.15	1		
RSES 9	All in all, I am inclined to feel that I am a failure.	−0.1	0.85	−0.13	−0.09	0.56	0.63	−0.05	0.71	1	
RSES 10	I take a positive attitude toward myself.	0.55	0.01	0.58	0.63	−0.01	−0.03	0.64	−0.08	−0.04	1
Item-total correlation		**0.49**	**0.61**	**0.50**	**0.58**	**0.47**	**0.59**	**0.62**	**0.46**	**0.61**	**0.59**
Mean		2.05	2.39	1.92	1.98	2.32	2.30	2.02	2.57	2.31	1.97
SD		0.94	0.86	0.81	0.84	0.86	0.91	0.83	0.87	0.83	0.86
Skewness		0.58	−0.02	0.90	0.69	0.32	0.21	0.59	−0.39	0.28	0.72
Kurtosis		−0.55	−0.68	0.75	0.05	−0.47	−0.74	−0.08	−0.53	−0.40	0.00

SD = standard deviation

Table 3. Reliability Analysis for the Indonesian Adaptation of the Rosenberg Self-Esteem Scale (*n* = 260)

Items	% floor effect	% ceiling effect	Cronbach's alpha	Test-retest reliability		AVE	CR
				ICC	95% CI		
Positive self-esteem			0.89	0.93	0.88–0.96	0.69	0.92
RSES 1	9.20	39.60					
RSES 3	6.50	54.20					
RSES 4	6.50	48.80					
RSES 7	5.80	48.80					
RSES 10	6.90	46.90					
Negative self-esteem			0.88	0.87	0.78–0.93	0.68	0.91
RSES 2	8.50	38.10					
RSES 5	10.40	47.30					
RSES 6	10.40	40.00					
RSES 8	10.80	50.00					
RSES 9	8.50	47.70					

CI = confidence interval, ICC = intraclass correlation coefficient, AVE = average variance extracted, CR = composite reliability,

Table 4. The model fits criteria of the RSES Indonesian version.

Item	Model 1a	Model 1b	Model 2	Model 3
X^2/df	24.50	2.96	2.93	2.93
GFI	0.55	0.93	0.93	0.93
AGFI	0.30	0.88	0.88	0.88
CFI	0.48	0.97	0.96	0.96
TLI	0.33	0.94	0.95	0.95
IFI	0.48	0.97	0.96	0.96
SRMR	0.19	0.03	0.04	0.04
RMSEA	0.30	0.08	0.08	0.08
AIC	897.634	133.992	141.497	141.497
BIC	968.848	240.812	216.27	216.27

df = degree of freedom GFI = goodness of fit index; AGFI = adjusted goodness of fit index; CFI = comparative fit index; TLI = Tucker-Lewis index; IFI = incremental fit index; SRMR = standardized root mean square residual; RMSEA = root mean square error of approximation; AIC = Akaike information criterion; BIC = Bayesian information criterion

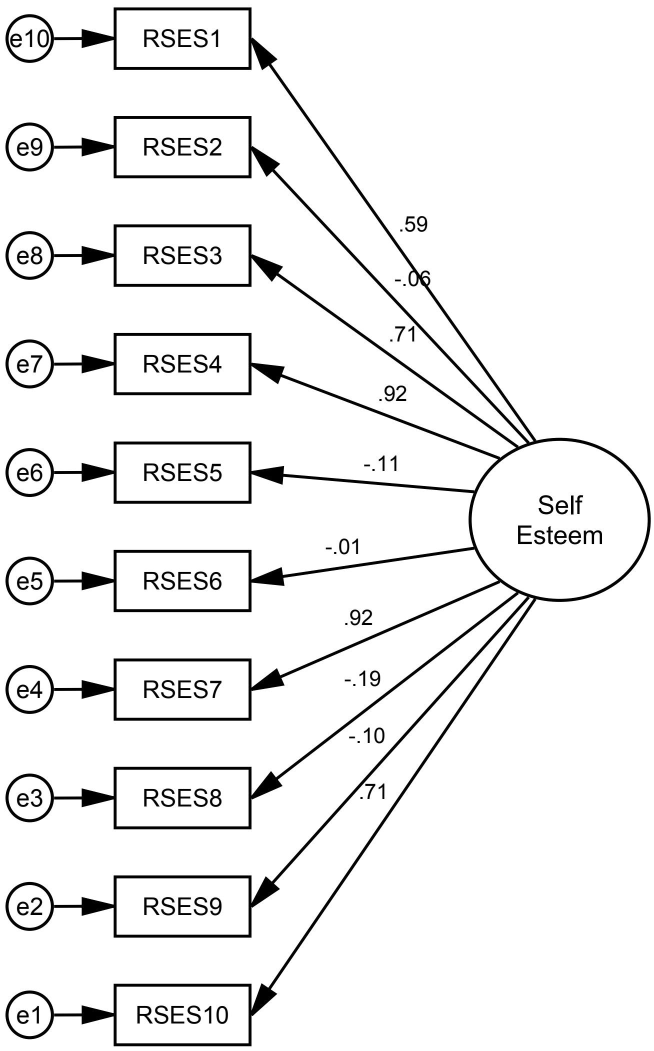

Figure 1. Single-factor model of RSES-Indonesian version (Model 1a)

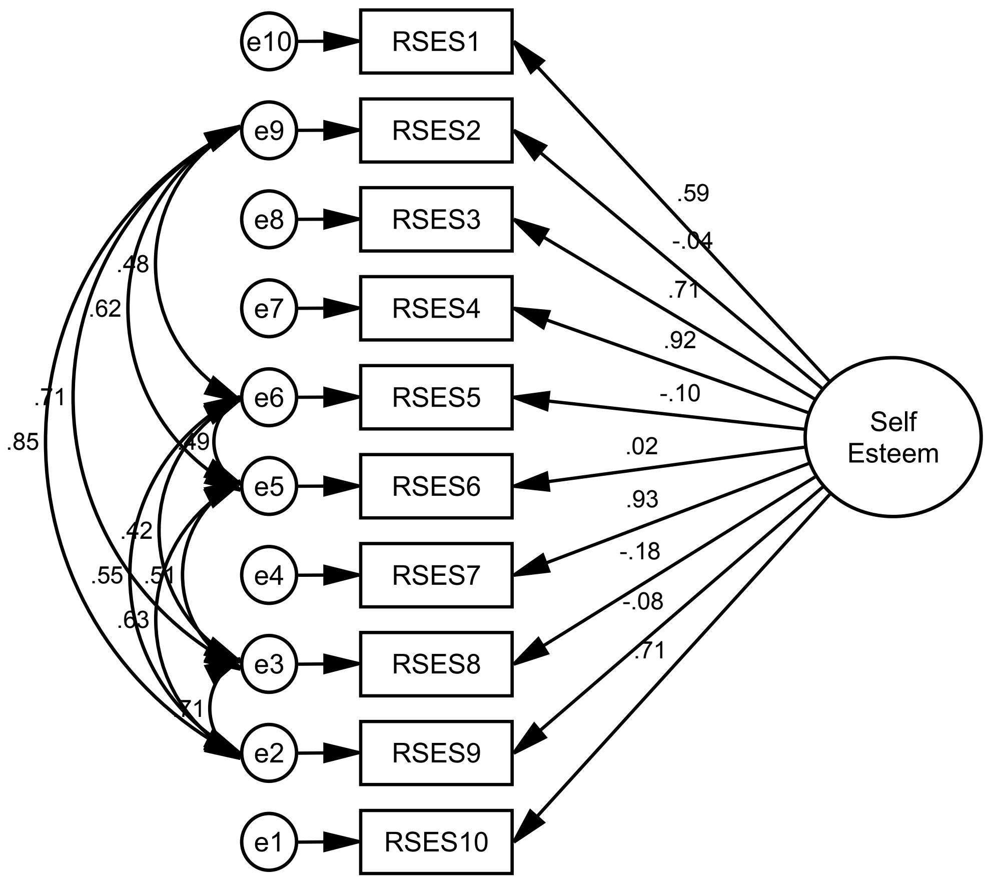

Figure 2. Single-factor model with correlated error (Model 1b)

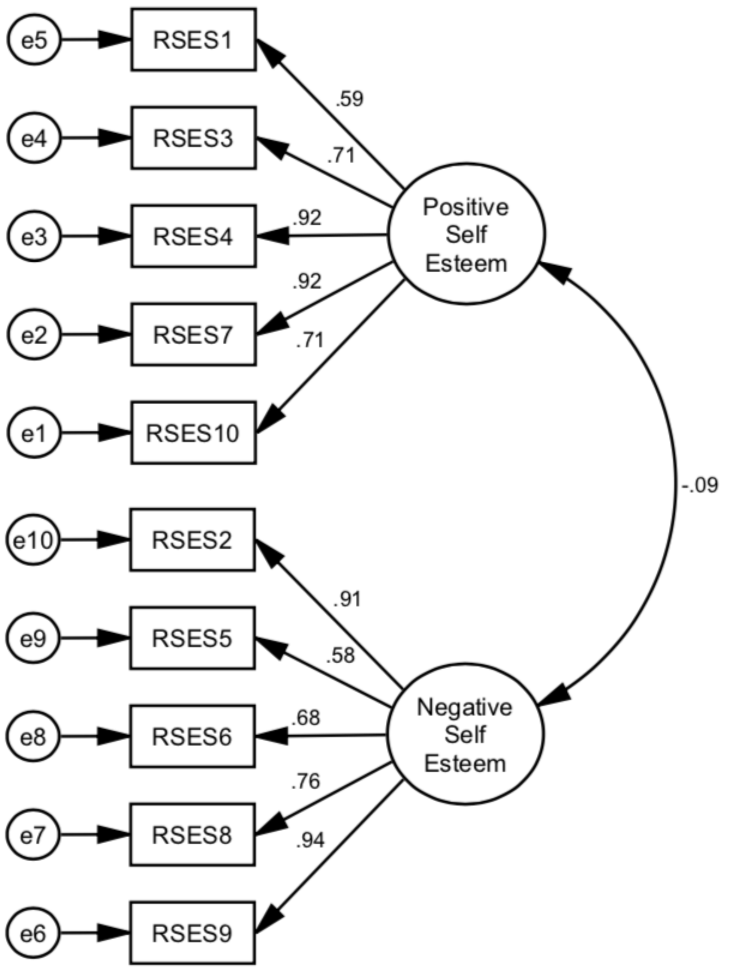

Figure 3. Two-factor model of RSES-Indonesian version (Model 2)

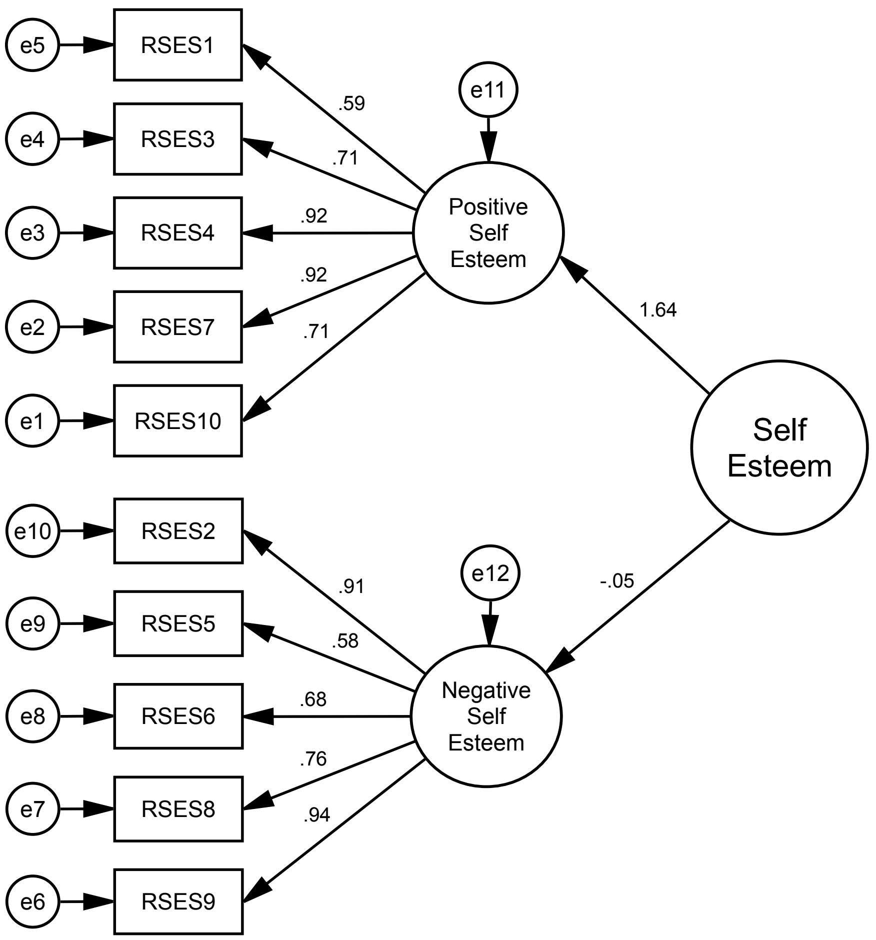

Figure 4. Hierarchical factor model of RSES-Indonesian version (Model 3)

Click here to access/download
Supporting Information
S1 Dataset.xlsx

Construct Validity of the Rosenberg Self-Esteem Scale in patientsPatients with Schizophrenia in Indonesia

Muhammad Muslih [a, b], Min-Huey Chung [a, c*]

[a] School of Nursing, College of Nursing, Taipei Medical University, Taiwan

[b] School of Nursing, Faculty of Health Science, University ofUniversitas Muhammadiyah Malang, Indonesia

[c] Department of Nursing, Shuang Ho Hospital, Taipei Medical University, New Taipei City, Taiwan

Corresponding Author:

Min-Huey Chung, PhD, RN, FAAN

School of Nursing, College of Nursing, Taipei Medical University

No. 250, Wu-Xing Street, Taipei, Taiwan, 110, ROC.

Email address: minhuey300@tmu.edu.tw

Abstract

Background: The Rosenberg self-esteem scale (RSES) is a commonly employed instrument for measuring self-esteem in the general population and those with mental illness. However, confirmatory factor analyses (CFA) to determine the factor structure and model fit of the RSES for schizophrenia patients are limited.However, exploratory and confirmatory factor analyses (EFA and CFA) to determine the factor structure and model fit of the RSES for schizophrenia patients have not been conducted.

Objectives: We examined the construct validity and reliability evidence of the RSES by measuring exploratory and confirmatory factors among patients with schizophrenia in Indonesia.

Methods: The sample comprised 260 participants. Over two weeks, 30 subjects were added to investigate test-retest reliabilityThirty participants were added to assess test-retest reliability over two weeks. We used internal consistency (Cronbach's alpha) and test-retest reliability Internal consistency (Cronbach's alpha) and test-retest reliability were utilized to evaluate the reliability evidence. The validity test evidence was based on an EFA to examine the factor structure and a CFA to determine the model fit.

Results: The RSES had an alpha Cronbach coefficient of 0.75. While 0.89 and 0.88 for the positive and negative self-esteem subscale, respectively. Test-retest reliability yielded adequate results with an interclass correlation score ranging from 0.87 to 0.93. Four different models were analyzed in this study. Considering the single-factor model (Model 1a), the overall fit criteria were inadequate. However, after some modification indices, all fit criteria were significantly adequate (Model 1b). The adequacy of all fit standards remained satisfactory when the two-factor model (Model 2) and hierarchical model (Model 3) were applied.The EFA validated a two-factor model. The value of 0.83 was obtained from the Kaiser-Meyer-Olkin test, and the Bartlett test of sphericity was significant (*p<0.001*). The ratio of the chi-square to the degree of freedom was 2.93 (*p<0.001*). The goodness-of-

fit index was 0.93, the adjusted goodness-of-fit index was 0.88, the comparative fit index was 0.96, the Tucker–Lewis index was 0.95, the incremental fit index was 0.96, and the standardized root means square residual was 0.04. The root mean square error of the approximation was 0.08.

Conclusions: The current investigation provided evidence supporting the construct validity and reliability of the RSES, indicating that the RSES can be considered a valid and reliable measurement. This finding suggests that the use of the RSES is beneficial and applicable in assessing levels of self-esteem in individuals diagnosed with schizophrenia in IndonesiaThe present study demonstrated that the RSES has satisfactory construct validity and reliability levels. This indicates that the RSES is valuable and practical for measuring self-esteem among patients with schizophrenia in Indonesia.

Keywords: self-esteem; validity; reliability, schizophrenia

1. Introduction

Self-esteem is an overall individual evaluation or appraisal of the self [1] and how a person thinks of themselves. Self-esteem is also defined as """the degree to which a person values, approves of, or likes himself or herself."" [2]. Self-esteem is a crucial component of mental health and general psychological well-being. It influences an individual'sindividual's achievements and successes, social interactions, and the ability to cope with environmental stressors [3, 4]. Individuals with high self-esteem believe they possess many positive qualities and attitudes toward themselves [5]. In summary, self-esteem is a pivotal psychological construct that controls several facets of an individual's existence, encompassing mental well-being, accomplishments, interpersonal engagements, and coping abilities.

There exists a reciprocal relationship between self-esteem and mental illnesses. A previous study found that self-esteem plays a pivotal role in developing diverse mental illnesses and social problems encompassing a range of internalizing issues, such as depression, suicidal tendencies, eating disorders, and anxiety, as well as externalizing problems, including violence and substance abuse According to

literature[6]. Conversely, it has been hypothesized that mental illnesses can lead to low self-esteem as a significant consequence, self-esteem also plays a crucial role in developing various mental illnesses. This is because low self-esteem has been postulated to be one of the primary outcomes of mental illness [7]. Consistent with prior research [8] low self-esteem has been found to heighten susceptibility to the onset of mental illness. In contrast, the existence of mental illness subsequently diminishes self-esteem. Eventually, individuals with mental illnesses are likely to have fluctuating self-esteem levels. Hence, self-esteem is considered a component of self-assessment, which influences mental health and vice versa.

Therefore, self-esteem is considered a component of self-assessment, which influences mental health. Individuals with mental illnesses are likely to have fluctuating self-esteem levels.

The Rosenberg Self-Esteem Scale (RSES), which was developed by Rosenberg (9), is one of the most extensively used instruments for measuring self-esteem globally [10-14]. Researchers often use the RSES to measure self-esteem in the clinical population [15]. In a prior study, the factor structure of the RSES was examined using psychometric tests, including just adolescents as participantsand it focused on adolescents [16], as they were the original target population of this scale. It has also been tested in adults [17] and the general population [18]. Other studies have tested the RSES in specific people, such as ex-prisoners [19], drug users [20], and single mothers [21]. Accordingly, the RSES applies to participants from various samples or populations. Nevertheless, there is no available evidence supporting the utilization of RSES among individuals diagnosed with schizophrenia in Indonesia.

The RSES has been translated and adapted into a variety of languages, including German [18], Dutch [22], Estonian [23], French [24], Portuguese [25], Japanese [14], and Thai [11]. It has been adapted across 53 nations with distinct ethnic groups and cultures [26]. This finding indicates that the RSES is widely used to measure self-esteemThis demonstrates that the RSES is a popular instrument used to measure self-esteem. Supporting this idea, Sinclair, Blais (10) suggest that the popularity of

the RSES can be attributed to its brevity and simplicity, as it comprises only ten questions that can be completed within a short timeframe of 1 to 2 minutes.explains that the RSES is popular because it is brief and straightforward and only includes ten questions, which can be answered within 1 to 2 minutes.

Multiple countries, including Indonesia, have adopted implemented the RSES to measure the self-esteem of college students and the general population college student's and the general population's self-esteem [26]. Interestingly, Additionally, some previous studies have shown that self-esteem has been associated with schizophrenia [27-29]. For example, there was a significant correlation between a decrease in the intensity of adverse symptoms and an enhancement in self-esteem, and conversely, [30]. Unfortunately, the scale for people diagnosed with schizophrenia was not evaluated using psychometric testing in a prior studyHowever, a previous study by [26]. did not use psychometric testing to evaluate the scale in people diagnosed with schizophrenia. Moreover,Additionally, they only focused on internal consistency and factor structure invariance.

Nevertheless, there is no available evidence supporting the utilization of RSES among individuals diagnosed with schizophrenia in Indonesia. Hence, evaluating the RSES in individuals diagnosed with schizophrenia is imperative to ascertain its validity and reliability. This study aimed to assess the construct validity of the RSES as a measure for patients with schizophrenia in Indonesia through confirmatory factor analyses (CFA).Therefore, it is essential to assess the RSES for patients who have schizophrenia to determine its construct validity and reliability. In this study, patients with schizophrenia in Indonesia were given exploratory and confirmatory factor analyses (EFA and CFA) to test the reliability and validity of the RSES as a measure of construct validity.

2. Methods

2.1 Participants

This is an instrumental questionnaire validation studyThis study employed a cross-sectional design. Two psychiatric hospitals and one psychiatric rehabilitation center in East Java, Indonesia, were visited to obtain the required data. We distributed the questionnaire from August 2018 to February 2019. Participants were recruited using the convenience sampling technique. The following requirements had to be met for someone to be included: (a) they had been diagnosed with schizophrenia; (b) aged ≥ 20 years; (c) hospitalized in a psychiatric ward; and (d) able to speak, read, and write Indonesian.; and (e) provision of informed consent. The Mini-Mental State Examination (MMSE) was utilized to screen out participants with cognitive impairment (i.e., MMSE scores < 24).

The size of 200 participants required in this study is acceptable based on recommendations from prior studies [31, 32]. In this study, data was missing from four questionnaires because they were not accurately completed, and 21 participants were excluded because their MMSE score was less than 24. Considering the response rate of 20%, the final sample comprised 260 participants. In addition, we recruited an additional 30 individuals to investigate test-retest reliability over two weeks.

The participants were informed before signing the written consent form that participation is voluntary and their confidentiality was carefully guarded. The purposes of the study, procedure for filling out questionnaires, potential risk or inconvenient, confidentiality issues and contact detail of the researcher were explained in the written consent forms and informs the participant directly. The researcher also gives an option to the participants to not participate in the study. The participants in this study was vulnerable group (psychiatric patients/ case with mental illness), therefore, additional protection was provided. The researcher was involved with the nurse and must be under supervision by the nurse in charge during data collection.

The minimum number of participants recommended by was 20 times the number of variables, which in this case equaled 200 participants. In this study, data was missing from four questionnaires

because they were not accurately completed, and 21 participants were excluded because their MMSE score was less than 24. Considering the response rate of 20%, the final sample comprised 260 participants. In addition, we recruited an additional 30 individuals to investigate test-retest reliability over two weeks.

2.2 Instruments

The study incorporates many socio-demographic data, including age as a continuous variable. The remaining variables as categorical variables, namely gender (1=male; 2=female), marital status (1=single; 2=married; 3=divorced, or widowed), employment status (1=employed; 2=unemployed), source of income (1=personal income; 2=family support; 3=personal and family support), education (1=elementary; 2=junior; 3=high school; 4=university/ college), previous hospitalization (1=yes; 2=no), and onset of illness (1=<1 year; 2=1-5 years; 3=>5 years).

The RSES is not licensed and; therefore, it is available for public use. Information about the scale can easily be gathered, and permission to use this resource can be sought at https://socy.umd.edu/about-us/rosenberg-self-esteem-scale. The scale consists of 10 items evaluated on a 4-point Likert scale from 1 (strongly disagree) to 4 (strongly agree). Scores vary from 10 to 40, with higher scores indicating a more positive self-esteem appraisal. To measure the reverse score, five questions are worded positively (items 1, 3, 4, 7, and 10), while five are worded negatively (items 2, 5, 6, 8, and 9).

The validity and reliability of the RSES were assessed in prior studies, and the obtained results are presented herein. According to a previous study [33] it is suggested to ensure that the Cronbach alpha criteria for each sub-scale is ≥ 0.70. The Cronbach's alpha coefficients for the positive and negative self-esteem subscales were determined to be 0.96 and 0.98, respectively [19]. A prior study conducted on individuals who are native English speakers has also yielded Cronbach alpha coefficients of 0.87 and 0.75 for the subscales measuring positive and negative self-esteem, respectively [14].

Concerning the evaluation of validity evidence (construct validity), prior studies have demonstrated that the RSES yielded an excellent model fit [19, 34].

found that the dependability of the RSES was satisfactory, with a Cronbach's alpha of 0.91. Similar to the Dutch version , reported that Cronbach's alpha of the Thai version of the scale was 0.86. A study conducted across 53 nations also revealed that the mean reliability necessary was 0.81 . tested the construct validity by correlating the RSES score with the NEO Five-Factor Inventory subscales. In line with , the RSES negatively correlated with the Thai Depression Inventory.

2.3 Translation Procedure

Due to their specificity and straightforwardness, we adhered to the parameters suggested by Sousa and Rojjanasrirat (35). Initially, the original questionnaire was separately translated into Indonesian by two translators, a psychiatrist and a professional translator whose native language was Indonesian, with the author'sauthor's approval. Both were bilingual and English-proficient. Second, we compared the two translated versions and created a new draft by combining the terminology and phrases supplied by the two translators in the previous step. Thirdly, the information was back-translated by two more independent translators with the same credentials and qualities as the first translators. Fourth, we compared the original questionnaire with the two back-translations of the questionnaire from the third phase. Considering the distance and time variations, we communicated with all four translators by email at this stage. Fifth, 30 volunteers were selected from a psychiatric hospital to evaluate the clarity of the questionnaire'squestionnaire's instructions, items, and response format. In addition, we asked two professionals (a psychiatrist and a psychologist) for revisions and ideas. In the final phase, the full Indonesian version of the RSES scale was administered to the study sample and tested the evidence of validity and reliability.In the last phase, the complete Indonesian version of the RSES scale was administered to the study sample, and its validity and reliability were determined.

2.4 Statistical Analysis

This study utilized SPSS and AMOS version 23.0 software (IBM; Armonk, New York, USA). All statistical significance was indicated by a *p*-value < 0.05.

2.4.1 Descriptive analysis

Descriptive statistics were used to present the demographic characteristics of the study. The proportion of participants who obtained minimum and maximum scores is defined as floor and ceiling effect, respectively. Continuous variables are presented as means and standard deviations, whereas categorical variables are expressed as frequencies and percentages. The quantitative characteristics of the RSES were computed as mean, standard deviation (SD), skewness, and kurtosis. A skewness value of between −1 and 1 was considered adequate [36]. The degree of vertical spread in the mean distribution corresponded to the kurtosis. Morgan and Griego (37) said that normality was assumed if the kurtosis value was less than 2.5 times the standard error.

2.4.12.4.2 Reliability evidence

Internal consistency (Cronbach'sCronbach's alpha) and the interclass correlation coefficient (ICC) were used to evaluate the RSES'RSES' test-retest reliability. A Cronbach'sCronbach's alpha equal to or better than 0.70 demonstrates adequate internal consistency [38, 39]. suggested that an The ICC score between 0.75 and 0.90 indicated satisfactory reliability, and consistency between two-time measurements, and a score greater than 0.90 revealed excellent reliability [40]..

2.4.22.4.3 Validity evidence

We carried out an EFA (exploratory factor analysis) to examine the construct validity of the scale using a principal component analysis with varimax rotation . Sampling adequacy was tested and confirmed using the Kaiser-Meyer-Olkin (KMO) and Bartlett's sphericity tests . The KMO index runs from 0 to 1 , and a value greater than 0.60 is preferred . Bartlett's test of sphericity yields significant results at $p < 0.001$. Again, to determine the number of components, an eigenvalue greater than 1.00 was utilized .

Composite reliability (CR), average variance extracted (AVE), and component loading were measures of convergent validity. The values of CR were more significant than 0.70 ; AVE was more critical than 0.50 , whereas the item loading was less than 0.50 . If the square root of AVE in each construct was more significant than the value of the other correlation constructs, the discriminant validity was satisfactory .

A CFA was carried out toin order to assess how well the RSES model fits the data. The following fit indices were utilized during the evaluation process: X^2/df, the comparative fit index (CFI), incremental fit index (IFI), the Tucker–Lewis index (TLI), goodness-of-fit index (GFI), adjusted goodness-of-fit index (AGFI), the standardized root mean square residual (SRMR), and the root mean square error of approximation (RMSEA). A chi-square with a degree of freedom ratio of less than 5.0 indicated that the model was a good fit [41]. An acceptable model fit was characterized by a GFI greater than 0.80 [42] and an AGFI in the range of 0.80 to 0.90 [43-47]. When the CFI, IFI, and TLI values were all greater than 0.90 [48], the SRMR value was less than 0.08 [48, 49], and the RMSEA value was less than 0.10 [42], the model fit was deemed to be satisfactory. Akaike Information Criterion (AIC) and Bayesian Information Criterion (BIC) were used to evaluate the alternative model, with lower AIC and BIC values indicating the best model fit [50, 51].

The composite reliability (CR) measurement and average variance extracted (AVE) are integral components of the comprehensive construct validity assessment. The values of CR were more significant than 0.70 [52], and AVE was more critical than 0.50 [53].

2.5 Ethical Approval

The Ethics Research Committee approved this research of the University of Muhammadiyah Malang on July 19, 2018 (approval number: E.5.a/239/KEPK- UMM/VII/2018).

3. Results

Table 1 presents the clinical demographics of the study. The average mean age of the 260 participants was 38.13 (SD = 9.56). Most participants were men (169, 65%) and single (139, 53.5%). MostThe majority were unemployed (173, 66.5%) and received financial support from their families (196, 75.4%). For 127 participants (48.8%), the highest educational level was senior high school. In total, 220 participants (84.6%) had previously been hospitalized, and the illness duration to onset was >5 years for 142 participants (54.6%).

Table 2 displays the descriptive statistics, inter-item correlation, and item-total correlation of the RSES. Item 8 and item 3 received the highest and lowest mean scores of 2.57 (SD = 0.87) and 1.92 (SD = 0.81), respectively. The skewness score ranged from 0.39 to 0.90 for the total RSES items, and the kurtosis score ranged from 0.74 to 0.75. The item-total correlation varied from 0.47 to 0.6. Floor and ceiling effect was found at 5.80% - 54.20%, respectively (Table 3).

3.1 Reliability evidence

The RSES had an alpha coefficient of 0.75, according to Cronbach'sCronbach's method. The results of Cronbach'sCronbach's alpha, which measures internal consistency, came in at 0.89 and 0.88 for each subscale (factor), indicating acceptable reliability. Test-retest reliability exhibited satisfactory results, with an ICC ranging between 0.87 and 0.93 (Table 3).

3.2 EFA

Based on the criteria regarding an eigenvalue >1.00, two factors were obtained. The outcomes of the factor analysis performed on the RSES are detailed in Table 3. A value of 0.83 was obtained from the KMO test, and the Bartlett test of sphericity was significant ($p<0.001$). The PCA with varimax rotation revealed that the factor loading of the RSES for all items exceeded 0.50 and was divided into two factors, positive self-esteem and negative self-esteem.

3.33.2 Validity evidenceand CFA

As presented in Table 3, the The CR was calculated for both positive and negative factors, and the values were 0.92 and 0.91, respectively. The AVE values were 0.69 and 0.68, and the square roots of

the AVE were 0.83 and 0.82, indicating that each measured variable was significant. Figure 1 displays the goodness of fit for the RSES model structure. The model obtained $X^2/df = 2.93$, $p < 0.001$, GFI = 0.93, AGFI = 0.88, CFI = 0.96, TLI = 0.95, IFI = 0.96, SRMR = 0.04, and RMSEA = 0.08.

The goodness of indices for all alternative models is shown in Table 4. Considering the single-factor model (Model 1a), the overall fit criteria were inadequate (See Figure 1). However, after some modification indices (See Figure 2), all fit criteria were significantly adequate (Model 1b). The adequacy of all fit criteria remained satisfactory when the two-factor model (Model 2) and hierarchical model (Model 3) were applied (See Figures 3 and 4).

4. Discussion

This study aimedThe purpose of this study was to establish the construct validity of the RSES. We included patients with schizophrenia in our study, which was not the case in the study by Schmitt and Allik (26). Our sample size was adequate to perform factor analysis. In addition, evidence of the construct validity of the RSES was demonstrated through EFA and CFA, contrary to previous studies conducted in Indonesia.

The total score on the Indonesian version of the RSES had a Cronbach'sCronbach's alpha coefficient of 0.75. In contrast, the positive self-esteem subscale had a coefficient of 0.89, and the negative self-esteem subscale had a coefficient of 0.88. In line with this finding, a previous investigation found satisfactory levels of internal consistency, as measured by a Cronbach'sCronbach's alpha coefficient that ranged from 0.81 to 0.91 [10, 11, 22, 26]. Our results were also consistent with a previous study by [54], which was conducted in individuals with severe mental illnesses, not specific only to patients with schizophrenia, and which reported strong internal consistency. This study also showed that the RSES has good internal consistency, especially for a particularcertain group of people (those with schizophrenia).

In addition, we looked at the test-retest reliability of the RSES. The ICC values that were generated were 0.93 for the positive self-esteem subscale and 0.87 for the negative self-esteem subscale. The ICC results were adequate, indicatingresults of the ICC were adequate, which indicated the stability of each factor of the RSES. Additionally, it was observed that We also noted that there was a high correlation coefficient between the test-retest reliability and Cronbach's alphathe correlation coefficient between test-retest and Cronbach's alpha was high. In conclusion, the findings of this study provide evidence supporting the robust reliability of the RSES as a reliable instrument for assessing self-esteem in individuals diagnosed with schizophrenia in the Indonesian contextIn sum, this result demonstrated that the RSES has strong reliability and is suitable for measuring self-esteem among patients with schizophrenia in Indonesia.

.An EFA was carried out in order to determine the components of the RSES, and the results revealed the presence of two factors: positive and negative self-esteem. The findings of several other studies on the RSES factor structure support the two-factor model that was measured in this study. Our finding supports the previous research by , which also concluded that the RSES comprises two factors. A meta-analysis by reported that most studies support the two-factor structure of the RSES. Another study indicated that a two-factor model considerably increases the fit compared to a one-factor model . Finally, our analysis based on patients with schizophrenia provided evidence that a two-factor model has a good fit.

We examine four models in our study: the single-factor (Model 1a), the single-factor with correlated error (Model 1b), the two-factor (Model 2), and the hierarchical model (Model 3). The results of Model 1a indicate that there is inadequate evidence to support the adequacy of a single factor. A notable distinction was observed while implementing the correlated error in Model 1b, as it demonstrates an enhancement in the adequate fit criteria. This adjustment was carried out on the negative self-esteem factor, a negatively worded item. This finding aligns with some prior studies that

have examined the presence of method effects related to negative items on the RSES [55-57]. The model with a single factor with correlated error yielded the best model fit.

Surprisingly, adequate fit indexes were also obtained in Models 2 and 3. The two-factor and hierarchical models exhibit comparable model fit in their respective analyses. Based on the findings mentioned earlier, it is suggested that the Rosenberg Self-Esteem Scale (RSES) can be conceptualized as comprising two distinct constructs, specifically positive self-esteem and negative self-esteem. Similar findings were also demonstrated in a previous study that a two-factor model was deemed to have an adequate model fit [16, 19, 58, 59]. In summary, our evidence indicates that the RSES scale construct has an acceptable model fit, which means that the RSES is suitable for individuals with schizophrenia in Indonesia. In short, the evidence from our study shows that the RSES scale construct fits well, which means that the RSES is a valid scale to use with people with schizophrenia in Indonesia.

Based on the rationale mentioned earlier, our study's results indicate that the RSES is a unidimensional measure of overall self-esteem, as initially proposed by Rosenberg (9). As previously established by various studies, RSES was also shown to assess two separate variables (positive and negative self-esteem) [16, 19, 59]. According to a prior study [60] it has been firmly indicated that the RSES is uni-dimensional. However, the influence of wording impacts on scale items may result in or contribute to bi-dimensionality. Acknowledging the necessity of reassessing the utilization of the RSES and its theoretical foundations in administering the scale to target populations is imperative.

The construct validity was evaluated using a CFA that was carried out. Convergent validity was assessed using the CR, AVE, and factor loading values, while discriminant validity was assessed using the square root of the AVE. Both convergent and discriminant validity analyses indicated that our study met the adequate criteria for validity, as shown by the fact that both the CR score and the AVE score, as well as the square root of the AVE score, are higher than the threshold score. Based on our analysis, the RSES meets the recommended model fit criteria for a two-factor model.

As shown in Figure 1, all RSES factors had good factor loading values ranging from 0.58 to 0.94. The overall results indicated that our model presented adequate GFIs. In support of our findings, the CFA from a prior study revealed that two factors are required for a good model fit . demonstrated in a previous study that a two-factor model was deemed to have an adequate model fit. In summary, our study's evidence indicates that the RSES scale's construct has a satisfactory fit, which means that the RSES is a suitable scale to use in individuals with schizophrenia in Indonesia. In short, the evidence from our study shows that the RSES scale's construct fits well, which means that the RSES is a good scale to use with people with schizophrenia in Indonesia

The present study also has limitations. Because our study sample included only patients with schizophrenia, these findings cannot be extrapolated to populations with other mental illnesses. Moreover, a significant majority of our study's participants are men, leading to a more prominent interpretation of item scores among this group. Future studies should aim to expand the participant pool by including individuals diagnosed with a diverse range of mental illnesses while also ensuring a balanced representation of both genders. In addition to the limitations mentioned above, it is essential to note that our study produced favorable outcomes regarding the validity and reliability of evidence. Hence establishing the RSES as a valid and reliable questionnaire appropriate for implementation within the tested sample group.

The present study also has limitations. Because our study sample included only patients with schizophrenia, these findings cannot be extrapolated to populations with other mental illnesses. However, because our study yielded satisfactory results for validity and reliability, future research must recruit additional participants with various mental illness diagnoses.

5. Conclusion

The current investigation provided evidence supporting the construct validity and reliability of the RSES, indicating that the RSES can be considered a valid and reliable measurement. This finding suggests that the use of the RSES is beneficial and applicable in assessing levels of self-esteem in individuals diagnosed with schizophrenia in Indonesia. Nevertheless, further research is needed to understand better the characteristics of the method factors for different populations.

The present study demonstrated that the RSES has satisfactory construct validity and reliability levels. This indicates that the RSES is valuable and practical for measuring self-esteem among patients with schizophrenia in Indonesia.

Supporting information

S1 Dataset

Data availability

All relevant data are within the paper and its S1 Dataset files. Please inform the authors if data are being used.

Conflict of Interest

The authors declare that they have no conflicts of interest.

Funding

The authors did not receivereceived any funding for this study.

AcknowledgmentAcknowledgement

We would like to acknowledge Wallace Academic Editing Company for editing this manuscript.

Authors'Authors' Contributions

M.M collected, analyzed, and interpreted the data and drafted the manuscript. M.H.C studied and analyzed the data and co-drafted the manuscript.

References

1. Cast AD, Burke PJ. A theory of self-esteem. Social forces. 2002;80(3):1041-68.
2. Blascovich J, Tomaka J. Measures of self-esteem. Measures of personality and social psychological attitudes. 1991;1:115-60.
3. Battle J. Promoting self-esteem, achievement and well being: An effective instructional curriculum for all levels: James Battle and Associates; 1994.
4. Huang C, Dong N. Factor structures of the Rosenberg self-esteem scale. European Journal of Psychological Assessment. 2012.
5. Bonfine N. Stigma self concept and stigma resistance among individuals with mental illness [Disertation]. Ann Arbor: Kent State University; 2013.
6. Mann M, Hosman CM, Schaalma HP, de Vries NK. Self-esteem in a broad-spectrum approach for mental health promotion. Health Educ Res. 2004;19(4):357-72. Epub 2004/06/17. doi: 10.1093/her/cyg041. PubMed PMID: 15199011.
7. Link BG, Struening EL, Neese-Todd S, Asmussen S, Phelan JC. Stigma as a barrier to recovery: The consequences of stigma for the self-esteem of people with mental illnesses. Psychiatric services. 2001;52(12):1621-6.
8. Silverstone PH, Salsali M. Low self-esteem and psychiatric patients: Part I – The relationship between low self-esteem and psychiatric diagnosis. Annals of General Hospital Psychiatry. 2003;2(1):2. doi: 10.1186/1475-2832-2-2.
9. Rosenberg M. Society and the Adolescent Self-Image. Revised edition. Middletown, CT: Wesleyan University Press; 1989.
10. Sinclair SJ, Blais MA, Gansler DA, Sandberg E, Bistis K, LoCicero A. Psychometric properties of the Rosenberg Self-Esteem Scale: Overall and across demographic groups living within the United States. Evaluation & the health professions. 2010;33(1):56-80.
11. Tinakon W, Nahathai W. A comparison of reliability and construct validity between the original and revised versions of the Rosenberg Self-Esteem Scale. Psychiatry investigation. 2012;9(1):54.
12. Martin CR, Thompson DR, Chan DS. An examination of the psychometric properties of the Rosenberg Self-Esteem Scale (RSES) in Chinese acute coronary syndrome (ACS) patients. Psychology, health & medicine. 2006;11(4):507-21.
13. Martín-Albo J, Núñez JL, Navarro JG, Grijalvo F. The Rosenberg Self-Esteem Scale: translation and validation in university students. The Spanish journal of psychology. 2007;10(2):458-67.
14. Mimura C, Griffiths P. A Japanese version of the Rosenberg Self-Esteem Scale: Translation and equivalence assessment. Journal of Psychosomatic Research. 2007;62(5):589-94.
15. Salerno L, Ingoglia S, Coco GL. Competing factor structures of the Rosenberg Self-Esteem Scale (RSES) and its measurement invariance across clinical and non-clinical samples. Personality and Individual Differences. 2017;113:13-9.

16. Marsh HW, Scalas LF, Nagengast B. Longitudinal tests of competing factor structures for the Rosenberg Self-Esteem Scale: traits, ephemeral artifacts, and stable response styles. Psychological assessment. 2010;22(2):366.
17. Gana K, Saada Y, Bailly N, Joulain M, Hervé C, Alaphilippe D. Longitudinal factorial invariance of the Rosenberg Self-Esteem Scale: Determining the nature of method effects due to item wording. Journal of Research in Personality. 2013;47(4):406-16.
18. Roth M, Decker O, Herzberg PY, Brähler E. Dimensionality and norms of the Rosenberg Self-Esteem Scale in a German general population sample. European Journal of Psychological Assessment. 2008;24(3):190-7.
19. Boduszek D, Hyland P, Dhingra K, Mallett J. The factor structure and composite reliability of the Rosenberg Self-Esteem Scale among ex-prisoners. Personality and individual differences. 2013;55(8):877-81.
20. Wang J, Siegal HA, Falck RS, Carlson RG. Factorial structure of Rosenberg's Self-Esteem Scale among crack-cocaine drug users. Structural Equation Modeling. 2001;8(2):275-86.
21. Hatcher J, Hall LA. Psychometric properties of the Rosenberg self-esteem scale in African American single mothers. Issues in Mental Health Nursing. 2009;30(2):70-7.
22. Franck E, De Raedt R, Barbez C, Rosseel Y. Psychometric properties of the Dutch Rosenberg self-esteem scale. Psychologica Belgica. 2008;48(1):25-35.
23. Pullmann H, Allik J. The Rosenberg Self-Esteem Scale: its dimensionality, stability and personality correlates in Estonian. Personality and Individual differences. 2000;28(4):701-15.
24. Vallieres EF, Vallerand RJ. Traduction et validation canadienne- française de l'échelle de l'estime de soi de Rosenberg. International journal of psychology. 1990;25(2):305-16.
25. Vasconcelos-Raposo J, Fernandes HM, Teixeira CM, Bertelli R. Factorial validity and invariance of the Rosenberg Self-Esteem Scale among Portuguese youngsters. Social Indicators Research. 2012;105(3):483-98.
26. Schmitt DP, Allik J. Simultaneous administration of the Rosenberg Self-Esteem Scale in 53 nations: exploring the universal and culture-specific features of global self-esteem. Journal of personality and social psychology. 2005;89(4):623.
27. Moritz S, Veckenstedt R, Randjbar S, Vitzthum F, Karow A, Lincoln TM. Course and determinants of self- esteem in people diagnosed with schizophrenia during psychiatric treatment. Psychosis. 2010;2(2):144-53.
28. Haug E, Øie MG, Andreassen OA, Bratlien U, Romm KL, Møller P, et al. The association between anomalous self-experiences, self-esteem and depressive symptoms in first episode schizophrenia. Frontiers in human neuroscience. 2016;10:557.
29. Kim EY, Jang MH. The Mediating Effects of Self-Esteem and Resilience on the Relationship Between Internalized Stigma and Quality of Life in People with Schizophrenia. Asian Nursing Research. 2019;13(4):257-63. doi: https://doi.org/10.1016/j.anr.2019.09.004.
30. Jones RM, Hansen L, Moskvina V, Kingdon D, Turkington D. The relationship between self-esteem and psychotic symptoms in schizophrenia: A longitudinal study. Psychosis. 2010;2(3):218-26.
31. Ferrando Piera PJ, Lorenzo Seva U, Hernández Dorado A, Muñiz Fernández J. Decálogo para el Análisis Factorial de los Ítems de un Test. Psicothema. 2022.

32. Lloret-Segura S, Ferreres-Traver A, Hernandez-Baeza A, Tomas-Marco I. Exploratory item factor analysis: A practical guide revised and updated. Anales de Psicología. 2014;30(3):1151-69.
33. Prinsen CA, Mokkink LB, Bouter LM, Alonso J, Patrick DL, De Vet HC, et al. COSMIN guideline for systematic reviews of patient-reported outcome measures. Quality of life research. 2018;27:1147-57.
34. Xu ML, Leung SO. Effects of varying numbers of Likert scale points on factor structure of the Rosenberg Self- Esteem Scale. Asian Journal of Social Psychology. 2018;21(3):119-28.
35. Sousa VD, Rojjanasrirat W. Translation, adaptation and validation of instruments or scales for use in cross-cultural health care research: a clear and user-friendly guideline. Journal of Evaluation Clinical Practice. 2011;17:268-74. Epub 2010/09/30. doi: 10.1111/j.1365-2753.2010.01434.x. PubMed PMID: 20874835.
36. Indrayan A, Malhotra RK. Medical biostatistics: Chapman and Hall/CRC; 2017.
37. Morgan GA, Griego OV. Easy use and interpretation of SPSS for Windows: Answering research questions with statistics: Psychology Press; 1998.
38. Polit DF, Beck CT. Essentials of nursing research: Appraising evidence for nursing practice: Lippincott Williams & Wilkins; 2010.
39. Tavakol M, Dennick R. Making sense of Cronbach's alpha. International journal of medical education. 2011;2:53.
40. Koo TK, Li MY. A guideline of selecting and reporting intraclass correlation coefficients for reliability research. Journal of chiropractic medicine. 2016;15(2):155-63.
41. Marsh HW, Hocevar D. Application of confirmatory factor analysis to the study of self-concept: First-and higher order factor models and their invariance across groups. Psychological bulletin. 1985;97(3):562.
42. Meyers LS, Gamst G, Guarino AJ. Applied Multivariate Research: Design and Interpretation: SAGE Publications; 2006.
43. Bentler PM. Comparative fit indexes in structural models. Psychological bulletin. 1990;107(2):238.
44. Marsh HW, Balla JR, McDonald RP. Goodness-of-fit indexes in confirmatory factor analysis: The effect of sample size. Psychological bulletin. 1988;103(3):391.
45. Cole DA. Utility of confirmatory factor analysis in test validation research. Journal of consulting and clinical psychology. 1987;55(4):584.
46. Floyd FJ, Widaman KF. Factor analysis in the development and refinement of clinical assessment instruments. Psychological assessment. 1995;7(3):286.
47. Greenspoon PJ, Saklofske DH. Confirmatory factor analysis of the multidimensional students' life satisfaction scale. Personality and Individual Differences. 1998;25(5):965-71.
48. Hu Lt, Bentler PM. Cutoff criteria for fit indexes in covariance structure analysis: Conventional criteria versus new alternatives. Structural equation modeling: a multidisciplinary journal. 1999;6(1):1-55.
49. Hoyle RH. Structural equation modeling: Concepts, issues, and applications: Sage; 1995.
50. Akaike H. A new look at the statistical model identification. IEEE transactions on automatic control. 1974;19(6):716-23.
51. Schwarz G. Estimating the dimension of a model. The annals of statistics. 1978:461-4.

52. Grewal R, Cote JA, Baumgartner H. Multicollinearity and measurement error in structural equation models: Implications for theory testing. Marketing science. 2004;23(4):519-29.

53. Hair J, Anderson R, Black B, Babin B. Multivariate Data Analysis: Pearson Education; 2016.

54. Torrey WC, Mueser KT, McHugo GH, Drake RE. Self-esteem as an outcome measure in studies of vocational rehabilitation for adults with severe mental illness. Psychiatric Services. 2000;51(2):229-33.

55. Mullen SP, Gothe NP, McAuley E. Evaluation of the factor structure of the Rosenberg Self-Esteem Scale in older adults. Personality and individual differences. 2013;54(2):153-7.

56. Aluja A, Rolland J-P, García LF, Rossier J. Dimensionality of the Rosenberg Self-Esteem Scale and its relationships with the three-and the five-factor personality models. Journal of Personality Assessment. 2007;88(2):246-9.

57. Makhubela M, Mashegoane S. Psychological validation of the Rosenberg Self-Esteem Scale (RSES) in South Africa: Method effects and dimensionality in black African and white university students. Journal of Psychology in Africa. 2017;27(3):277-81.

58. Quilty LC, Oakman JM, Risko E. Correlates of the Rosenberg self-esteem scale method effects. Structural Equation Modeling. 2006;13(1):99-117.

59. Boduszek D, Shevlin M, Mallett J, Hyland P, O'Kane D. Dimensionality and construct validity of the Rosenberg self- esteem scale within a sample of recidivistic prisoners. Journal of Criminal Psychology. 2012;2(1):19-25.

60. Reise SP, Kim DS, Mansolf M, Widaman KF. Is the bifactor model a better model or is it just better at modeling implausible responses? Application of iteratively reweighted least squares to the Rosenberg Self-Esteem Scale. Multivariate behavioral research. 2016;51(6):818-38.

Table 1. Demographic Characteristics of the Study (*n* = 260)

Characteristics	Participants (*n* = 260)	
	Mean (SD)	n (%)
Age	38.13 (9.56)	
Gender		
Male		169 (65.00)
Female		91 (35.50)
Marital status		
Single		139 (53.465)
Married		81 (31.152)
Divorce or widowed		40 (15.394)
Employment status		
Employed		87 (33.50)
Unemployed		173 (66.50)
Source of income		
Personal income		30 (11.50)
Family support		196 (75.40)
Personal and family		34 (13.10)
Education		
Elementary school		63 (24.23)
Junior high school		44 (16.92)
Senior high school		127 (48.85)
University/college		26 (10.00)
Previous hospitalization		
Yes		220 (84.60)
No		40 (15.40)
Onset of illness		
<1 year		75 (28.85)
1–5 years		43 (16.54)
>5 years		142 (54.61)

*missing data = 4; **MMSE score < 24 = 21
SD = standard deviation; MMSE = Mini-Mental State Examination.

Table 2. Descriptive Statistics, Interitem, and Item-Total Correlation of the RSES Items (*n* = 260)

Items		Interitem correlation (*n* = 260)									
		1	2	3	4	5	6	7	8	9	10
RSES 1	On the whole, I am satisfied with myself.	1									
RSES 2	At times, I think I am no good at all.	−0.11	1								
RSES 3	I feel that I have a number of good qualities.	0.56	−0.12	1							
RSES 4	I am able to do things as well as most other people.	0.51	−0.04	0.63	1						
RSES 5	I feel I do not have much to be proud of.	−0.03	0.48	−0.13	−0.11	1					
RSES 6	I certainly feel useless at times.	−0.00	0.62	−0.04	−0.01	0.48	1				
RSES 7	I feel that I'mI'm a person of worth, at least on an equal plane with others.	0.52	−0.01	0.64	0.86	−0.07	0.06	1			
RSES 8	I wish I could have more respect for myself.	−0.19	0.71	−0.22	−0.15	0.43	0.50	−0.15	1		
RSES 9	All in all, I am inclined to feel that I am a failure.	−0.1	0.85	−0.13	−0.09	0.56	0.63	−0.05	0.71	1	
RSES 10	I take a positive attitude toward myself.	0.55	0.01	0.58	0.63	−0.01	−0.03	0.64	−0.08	−0.04	1
Item-total correlation		**0.49**	**0.61**	**0.50**	**0.58**	**0.47**	**0.59**	**0.62**	**0.46**	**0.61**	**0.59**
Mean		2.05	2.39	1.92	1.98	2.32	2.30	2.02	2.57	2.31	1.97
SD		0.94	0.86	0.81	0.84	0.86	0.91	0.83	0.87	0.83	0.86
Skewness		0.58	−0.02	0.90	0.69	0.32	0.21	0.59	−0.39	0.28	0.72
Kurtosis		−0.55	−0.68	0.75	0.05	−0.47	−0.74	−0.08	−0.53	−0.40	0.00

SD = standard deviation

Table 3. Factor Structure, Reliability Analysis for the Indonesian Adaptation of the Rosenberg Self-Esteem Scale (*n* = 260)Reliability, and Validity Analysis for the Indonesian Adaptation of the Rosenberg Self-Esteem Scale (*n* = 260)

Items	% floor effect	% ceiling effect	Cronbach's alpha	Test-retest reliability		AVE	CR
				ICC	95% CI		
Positive self-esteem			0.89	0.93	0.88–0.96	0.69	0.92
RSES 1	9.20	39.60					
RSES 3	6.50	54.20					
RSES 4	6.50	48.80					
RSES 7	5.80	48.80					
RSES 10	6.90	46.90					
Negative self-esteem			0.88	0.87	0.78–0.93	0.68	0.91
RSES 2	8.50	38.10					
RSES 5	10.40	47.30					
RSES 6	10.40	40.00					
RSES 8	10.80	50.00					
RSES 9	8.50	47.70					

CI = confidence interval, ICC = intraclass correlation coefficient, AVE = average variance extracted, CR = composite reliability,

KMO = 0.83, df = 45, Bartlett's test of sphericity ($p < 0.0001$)
KMO = Kaiser-Meyer-Olkin, df = degree of freedom, CI = confidence interval, ICC = intraclass correlation coefficient, AVE = average variance extracted, CR = composite reliability,
PSE = positive self-esteem, NSE = negative self-esteem.

Table 4. The model fits criteria of the RSES Indonesian version.

Item	Model 1a	Model 1b	Model 2	Model 3
X^2/df	24.50	2.96	2.93	2.93
GFI	0.55	0.93	0.93	0.93
AGFI	0.30	0.88	0.88	0.88
CFI	0.48	0.97	0.96	0.96
TLI	0.33	0.94	0.95	0.95
IFI	0.48	0.97	0.96	0.96
SRMR	0.19	0.03	0.04	0.04
RMSEA	0.30	0.08	0.08	0.08
AIC	897.634	133.992	141.497	141.497
BIC	968.848	240.812	216.27	216.27

df = degree of freedom GFI = goodness of fit index; AGFI = adjusted goodness of fit index; CFI = comparative fit index; TLI = Tucker-Lewis index; IFI = incremental fit index; SRMR = standardized root mean square residual; RMSEA = root mean square error of approximation; AIC = Akaike information criterion; BIC = Bayesian information criterion

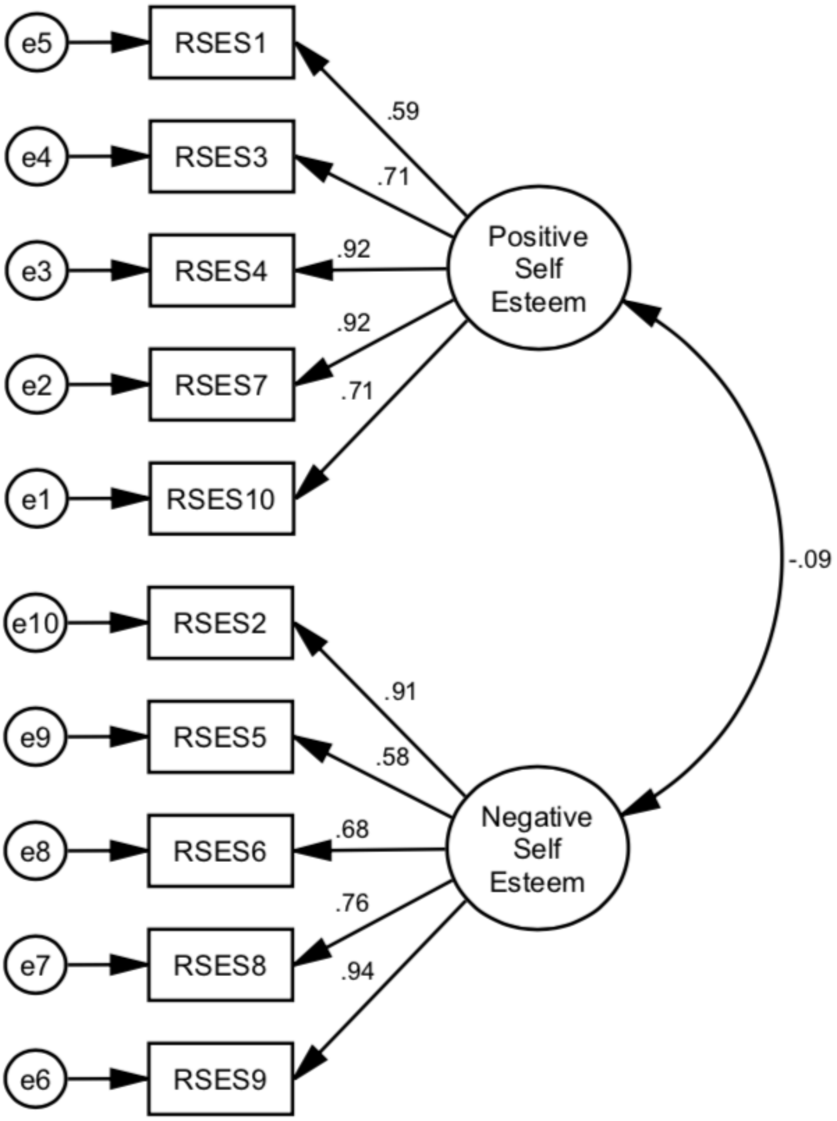

Figure 1. Model fit of the two-factor model of the RSES.
X^2/df = 2.93, $p < 0.001$, GFI = 0.93, AGFI = 0.88, CFI = 0.96,
TLI = 0.95, IFI = 0.96, NFI = 0.94, SRMR = 0.04, RMSEA = 0.08.
