# Supplementary material for: Structural validity of the Rosenberg self-esteem scale in patients with schizophrenia in Indonesia
Source: PLoS One. 2024 May 10;19(5):e0300184. doi: 10.1371/journal.pone.0300184 (PMC11086833; doi:10.1371/journal.pone.0300184)
Supplement: Attachment — Submitted filename: PONE-D-23-08354_R2_reviewer.pdf [file pone.0300184.s004.pdf]

PLOS ONE

Structural Validity of the Rosenberg Self-Esteem Scale in Patients with Schizophrenia in Indonesia

--Manuscript Draft--

**Manuscript Number:**	PONE-D-23-08354R2
**Article Type:**	Research Article
**Full Title:**	Structural Validity of the Rosenberg Self-Esteem Scale in Patients with Schizophrenia in Indonesia
**Short Title:**	Structural Validity of the Rosenberg Self-Esteem Scale in patients with Schizophrenia in Indonesia
**Corresponding Author:**	Min-Huey Chung, Ph.D.<br>Taipei Medical University<br>Taipei, TAIWAN
**Keywords:**	self-esteem; validity; reliability, schizophrenia
**Abstract:**	Background: The Rosenberg self-esteem scale (RSES) is a commonly employed instrument for measuring self-esteem in the general population and those with mental illness. However, confirmatory factor analyses (CFA) to determine the structural validity of the RSES for schizophrenia patients in Indonesia are limited.<br>Objectives: We examined the structural validity of the RSES as a measurement for patients with schizophrenia in Indonesia through confirmatory factor analyses (CFA), as well as assessing internal consistency and reliability.<br>Methods: The sample comprised 260 participants. Over two weeks, 30 subjects were added to investigate test-retest reliability. The structural validity analyzed was based on a CFA to determine the model fit. We used internal consistency (Cronbach's alpha) to evaluate the reliability evidence.<br>Results: Four different models were analyzed in this study. Considering the single-factor model (Model 1a), the overall fit criteria were inadequate. However, after some modification indices, all fit criteria were significantly adequate (Model 1b). The adequacy of all fit standards remained satisfactory when the two-factor model (Model 2) and hierarchical model (Model 3) were applied. The RSES had an alpha Cronbach coefficient of 0.75. While 0.89 and 0.88 for the positive and negative self-esteem subscale, respectively. Test-retest reliability yielded adequate results with an interclass correlation score ranging from 0.87 to 0.93.<br>Conclusions: The current investigation provided evidence supporting the structural validity, internal consistency, and reliability of the RSES, indicating that the RSES can be considered a valid and reliable measurement. A two-factor model of RSES was an appropriate model to measure self-esteem in our study. This finding suggests that the use of the RSES is beneficial and applicable in assessing levels of self-esteem in individuals diagnosed with schizophrenia in Indonesia.
**Order of Authors:**	Muhammad Muslih
	Min-Huey Chung, Ph.D.
**Response to Reviewers:**	Dear Editor and Reviewer<br><br>Manuscript ID: PONE-D-23-08354R1<br>Manuscript Title: “Structural Validity of the Rosenberg Self-Esteem Scale in Patients with Schizophrenia in Indonesia”<br><br>We sincerely thank the editor and all reviewers for their valuable suggestions and for allowing us to revise our manuscript entitled “Construct Validity of the Rosenberg Self-Esteem Scale in Patients with Schizophrenia in Indonesia”. We have incorporated all the suggested changes into the manuscript and have highlighted the revised sections. At this moment, our responses and revisions are based on the editor and reviewer’s comments.<br><br>--

Powered by Editorial Manager® and ProduXion Manager® from Aries Systems Corporation

EDITORIAL COMMENTS:
Please note that the discussion, as it is now, is confusing. On the one hand they state that the scale is unidimensional, but then conclude that the best structure is the two-factor model. Furthermore, in order to reach a conclusion on this, other parameters, such as factor loadings, should be taken into consideration. Factor loadings below 0.30 should not be included in these items because they do not explain enough variance.
Response: Thank you for your correction. The necessary amendments and recommendations provided by the reviewers have been incorporated into our work. The discussion section of our study has been revised.
(Page 10)
The adequate fit indexes were also obtained in Models 2 and 3. The two-factor and hierarchical models exhibit comparable model fit in their respective analyses. Based on the findings mentioned above, it is suggested that the RSES can be characterized as two factors, which are positive and negative self-esteem. A previous study also referred to these two factors as positive and negative self-esteem [69]. The influence of wording effect on scale items may result in or contribute to a two-factor model. The wording item effect, then further related to the method effect has been observed in earlier studies [37, 70], which suggests the presence of a two-factor of RSES [70]. In summary, our finding indicates that the RSES scale has an acceptable model fit with two factors, Similar findings were also demonstrated in a previous study that a two-factor model was deemed to have an adequate model fit [21, 36, 71, 72]. Based on our finding, we can conclude that the RSES with is a two-factor model was a valid instrument for people with schizophrenia in Indonesia. Acknowledging the necessity of reassessing the utilization of the RSES and its theoretical foundations in administering the scale to target populations is essential.

(Page 12; conclusion).
The current investigation provided evidence supporting the structural validity, internal consistency, and reliability of the RSES, indicating that the RSES can be considered a valid and reliable measurement. A two-factor model of RSES was an appropriate model to measure self-esteem in our study. This finding suggests that the use of the RSES is beneficial and applicable in assessing levels of self-esteem in individuals diagnosed with schizophrenia in Indonesia.

With respect to loading factors, the initial manuscript we submitted details that our study employed loading factors ranging from 0.69 to 0.92, indicating that all loading factors exceeded 0.30. As previously mentioned in the last revision, our analysis was limited to CFA without EFA. We therefore do not report any factor loading in our recent revision.

REVIEWER #1 COMMENTS: No comments.

REVIEWER #2 COMMENTS:

I appreciate the authors' time spent in enhancing the manuscript. Statistically, it seems they have done an acceptable job. Nevertheless, there are still certain concerns I have

Powered by Editorial Manager® and ProduXion Manager® from Aries Systems Corporation

regarding the manuscript's writing (Attached is a PDF where I've highlighted in red the elements that strike me as discordant in the text):

INTRODUCTION:

1.In summary, self-esteem is a pivotal psychological construct that controls several facets of an individual's existence, encompassing mental well-being, accomplishments, interpersonal engagements, and coping abilities-->Evidence is needed for this statement.
Response: Thank you for your correction. We have corrected and replaced it following your suggestions.

(Page 3; paragraph 1)
In summary, self-esteem is a pivotal psychological construct [6] that controls several facets of an individual's existence, encompassing mental well-being, accomplishments, interpersonal engagements, and coping abilities [3, 4].

2.There exists a reciprocal relationship between self-esteem and mental illnesses. A previous study found that self-esteem plays a pivotal role in developing diverse mental illnesses and social problems encompassing a range of internalizing issues, such as depression, suicidal tendencies, eating disorders, and anxiety, as well as externalizing problems, including violence and substance abuse -->Evidence is needed for this statement. A visual representation or diagram displaying the relationship between constructs/variables would be interesting for the reader.
Response: Thank you for your correction and suggestion. We have corrected and replaced it following your suggestions.

(Page 3; paragraph 2)
There is a reciprocal relationship between self-esteem and mental illnesses [7].

3.Researchers often use the RSES to measure self-esteem in the clinical population [15]--> Please add the characteristics of the population, diagnosis...
Response: Thank you for your correction and suggestion. We have corrected and replaced it following your suggestions.

(Page 3; paragraph 3)
Researchers often use the RSES to measure self-esteem in the clinical population, such as eating disorders [18], anxiety, depression [7], attention and emotional disorder [19], schizophrenia and bipolar disorder [20]. Other studies have tested the RSES in specific people, such as ex-prisoners [21], drug users [22], and single mothers [23].

4.General Overview: The repetition of some ideas and the lack of a smooth transition between paragraphs make it difficult to read. Ensure that the citations are correctly referenced and that there is coherence in the bibliography throughout the text. Some key statements lack specific citations or references, which compromises the credibility of the text. Through a brief search, I find a large number of validations, such as those in Spanish, which the authors have not included in the relevant section of the introduction. An exposition of the structural differences and psychometric properties of the tool in the target population of the study should be made, in this case, in patients with schizophrenia.
Response: Thank you for your correction and suggestion. We have corrected and replaced it following your suggestions, including the substitution of multiple sentences in the introduction to make it easier for readers to fully understand our idea. (See page 3-4; paragraph 1-5)

(Page 3-4; paragraph 3)
The RSES has been translated and adapted into a number of different languages, including German [24], Dutch [25], Estonian [26], French [27], Portuguese [28], Spanish [29], Japanese [17], and Thai [14]; thus, making it applicable to participants from diverse samples or populations.

METHODS:
5.Instruments-->Remove the numbering from the categorical variables. Is the

Powered by Editorial Manager® and ProduXion Manager® from Aries Systems Corporation

instruments section appropriate to discuss the known evidence of internal consistency?
Response: Thank you for your correction and suggestion. We have corrected and replaced it following your suggestions. This section aims to elucidate the rationale for the utilization of this questionnaire. It will accomplish this by providing information about the questionnaire's developer, the scale employed, its interpretation, and the questionnaire's validity and reliability evidence, as established by prior research.

(Page 5)
The remaining variables as categorical variables, namely gender (male; female), marital status (single; married; divorced, or widowed), employment status (employed; unemployed), source of income (personal income; family support; personal and family support), education (elementary; junior; high school; university/ college), previous hospitalization (yes; no), and onset of illness (<1 year; 1-5 years; >5 years).

6.Translation procedure-->Include an explanatory diagram/figure of the process.
Response: Thank you for your correction and suggestion. We have provided the flow diagram of the translation procedure (see: Figure 1)
(Figure 1)

7.Statistical Analysis--> Please remove the sub-sections and write in a simpler manner.
Response: Thank you for your correction and suggestion. We have corrected and replaced it following your suggestions. (Page 7-8).

8.Validity evidence--> What estimator have you used for the CFA? ULS, WLSMV?
Response: Thank you for your correction and suggestion. Unweighted Least Squares (ULS) was used in our analysis, since our data meet the assumption as a continuous and normally distributed.

9.General Overview: Please present structural validity section before reliability and internal consistency. I refer you to COSMIN. The data on internal consistency cannot be interpreted before data on structural validity.
Response: Thank you for your correction and suggestion. We have corrected and replaced it following your suggestions, as mentioned based on the COSMIN.

RESULTS:
10.General Overview: Please provide the evidence for structural validity before internal consistency and reliability. According to COSMIN (reference provided in the first review), internal consistency and reliability cannot be interpreted without first having evidence of structural validity.
Response: Thank you for your correction and suggestion. We have corrected and replaced it following your suggestions.

(Page 9)
Structural validity.
The goodness of indices for all alternative models is shown in Table 3. Considering the single-factor or uni-dimensional model (M1a), the overall fit criteria were inadequate (See Figure 2). However, after some modification indices (See Figure 3), all fit criteria were significantly adequate (M1b). The adequacy of all fit criteria remained satisfactory

Powered by Editorial Manager® and ProduXion Manager® from Aries Systems Corporation

	when the two-factor model (M2) and hierarchical model (M3) were applied (See Figures 4 and 5). The AVE values were 0.69 and 0.68, and the square roots of the AVE were 0.83 and 0.82, indicating that each measured variable was significant (Table 4).<br><br>Internal consistency and reliability evidence.<br>The RSES had an alpha coefficient of 0.75, according to Cronbach's method. The results of Cronbach's alpha, which measures internal consistency, came in at 0.89 and 0.88 for each subscale (factor), indicating acceptable internal consistency. As presented in Table 4, the CR was calculated for positive and negative factors, and the values were 0.92 and 0.91, respectively. Test-retest reliability exhibited satisfactory results, with an ICC between 0.87 and 0.93 (Table 4).<br><br>DISCUSSION:<br>11.General Overview: The discussion still requires further development. Currently, the flow of information is disorganized. I also encourage the authors to delve into the results they have obtained and provide an explanation and reasoning behind their findings. They do not discuss the advantages of obtaining a single factor or the disadvantages of not obtaining it... I'm also unsure whether the studies they are comparing with have clinical or non-clinical samples. There is an anthropomorphic language that should be removed. The writing needs to be revised as it still does not adhere to AERA, APA, NMCE, COSMIN standards.<br>Response: Thank you for your correction and suggestion. We have corrected and replaced it following your suggestions. Such as; construct validity was replaced specifically as structural validity; and reliability was replaced and divided as internal consistency and reliability. The details are on pages 9-11.<br><br>Next, I copy fragments of the discussion that bother me when reading:<br><br>"Surprisingly", "can be conceptualized as comprising two distinct constructs", "In short, the evidence from our study shows that the RSES scale construct fits well", Our sample size was adequate to perform factor analysis", "Our results were consistent with a previous study by [54], conducted in", "individuals with severe mental illnesses, not specific only to patients with schizophrenia and reported strong internal consistency".......<br>Response: Thank you for your correction and suggestion. We have also improved the discussion section to make it easier for readers to understand what we mean in our study. The details are in the discussion section (pages 9-11). Furthermore, this manuscript was edited by Wallace Academic Editing Company to help us in providing writing corrections to our manuscript so that it complies with journal standards.<br><br>REVIEWER #3 COMMENTS:<br>1.Authors have successfully adressed all of my concerns. The manuscript can be considered for its publication now.<br>Response: We express our gratitude for your kind attention.
**Additional Information:**	
**Question**	**Response**
**Financial Disclosure**<br><br>Enter a financial disclosure statement that describes the sources of funding for the work included in this submission. Review the submission guidelines for detailed requirements. View published research articles from *PLOS ONE* for specific examples.<br><br>This statement is required for submission and **will appear in the published article** if	The authors did not received any funding for this study.

the submission is accepted. Please make sure it is accurate.<br><br>**Unfunded studies**<br>Enter: *The author(s) received no specific funding for this work.*<br><br>**Funded studies**<br>Enter a statement with the following details:<br>• Initials of the authors who received each award<br>• Grant numbers awarded to each author<br>• The full name of each funder<br>• URL of each funder website<br>• Did the sponsors or funders play any role in the study design, data collection and analysis, decision to publish, or preparation of the manuscript?<br>• **NO** - Include this sentence at the end of your statement: *The funders had no role in study design, data collection and analysis, decision to publish, or preparation of the manuscript.*<br>• **YES** - Specify the role(s) played.<br><br>* typeset	
**Competing Interests**<br><br>Use the instructions below to enter a competing interest statement for this submission. On behalf of all authors, disclose any competing interests that could be perceived to bias this work—acknowledging all financial support and any other relevant financial or non-financial competing interests.<br><br>This statement is required for submission and **will appear in the published article** if the submission is accepted. Please make sure it is accurate and that any funding sources listed in your Funding Information later in the submission form are also declared in your Financial Disclosure statement.<br><br>View published research articles from *PLOS ONE* for specific examples.	The authors declare that they have no conflicts of interest.

**NO authors have competing interests**<br><br>Enter: *The authors have declared that no competing interests exist.*<br><br>**Authors with competing interests**<br><br>Enter competing interest details beginning with this statement:<br><br>*I have read the journal's policy and the authors of this manuscript have the following competing interests: [insert competing interests here]*<br><br>* typeset	
**Ethics Statement**<br><br>Enter an ethics statement for this submission. This statement is required if the study involved:<br><br>• Human participants<br>• Human specimens or tissue<br>• Vertebrate animals or cephalopods<br>• Vertebrate embryos or tissues<br>• Field research<br><br>Write "N/A" if the submission does not require an ethics statement.<br><br>General guidance is provided below. Consult the submission guidelines for detailed instructions. **Make sure that all information entered here is included in the Methods section of the manuscript.**	The Ethics Research Committee approved this research of the University of Muhammadiyah Malang on July 19, 2018 (approval number: E.5.a/239/KEPK-UMM/VII/2018).The participants were informed before signing the written consent form that participation is voluntary and their confidentiality was carefully guarded. The purposes of the study, procedure for filling out questionnaires, potential risk or inconvenient, confidentiality issues and contact detail of the researcher were explained in the written consent forms and informs the participant directly. The researcher also gives an option to the participants to not participate in the study. The participants in this study was vulnerable group (psychiatric patients/ case with mental illness), therefore, additional protection was provided. The researcher was involved with the nurse and must be under supervision by the nurse in charge during data collection.

Powered by Editorial Manager® and ProduXion Manager® from Aries Systems Corporation

**Format for specific study types**<br><br>**Human Subject Research (involving human participants and/or tissue)**<br>• Give the name of the institutional review board or ethics committee that approved the study<br>• Include the approval number and/or a statement indicating approval of this research<br>• Indicate the form of consent obtained (written/oral) or the reason that consent was not obtained (e.g. the data were analyzed anonymously)<br><br>**Animal Research (involving vertebrate animals, embryos or tissues)**<br>• Provide the name of the Institutional Animal Care and Use Committee (IACUC) or other relevant ethics board that reviewed the study protocol, and indicate whether they approved this research or granted a formal waiver of ethical approval<br>• Include an approval number if one was obtained<br>• If the study involved *non-human primates*, add *additional details* about animal welfare and steps taken to ameliorate suffering<br>• If anesthesia, euthanasia, or any kind of animal sacrifice is part of the study, include briefly which substances and/or methods were applied<br><br>**Field Research**<br><br>Include the following details if this study involves the collection of plant, animal, or other materials from a natural setting:<br>• Field permit number<br>• Name of the institution or relevant body that granted permission	
**Data Availability**<br><br>Authors are required to make all data underlying the findings described fully available, without restriction, and from the time of publication. PLOS allows rare exceptions to address legal and ethical concerns. See the PLOS Data Policy and FAQ for detailed information.	Yes - all data are fully available without restriction

A Data Availability Statement describing where the data can be found is required at submission. Your answers to this question constitute the Data Availability Statement and **will be published in the article**, if accepted.<br><br>**Important:** Stating 'data available on request from the author' is not sufficient. If your data are only available upon request, select 'No' for the first question and explain your exceptional situation in the text box.<br><br>Do the authors confirm that all data underlying the findings described in their manuscript are fully available without restriction?	
**Describe where the data may be found in full sentences. If you are copying our sample text, replace any instances of XXX with the appropriate details.**<br><br>• If the data are **held or will be held in a public repository**, include URLs, accession numbers or DOIs. If this information will only be available after acceptance, indicate this by ticking the box below. For example: *All XXX files are available from the XXX database (accession number(s) XXX, XXX.*).<br>• If the data are all contained **within the manuscript and/or Supporting Information files**, enter the following: *All relevant data are within the manuscript and its Supporting Information files.*<br>• If neither of these applies but you are able to provide **details of access elsewhere**, with or without limitations, please do so. For example:<br><br>*Data cannot be shared publicly because of [XXX]. Data are available from the XXX Institutional Data Access / Ethics Committee (contact via XXX) for researchers who meet the criteria for access to confidential data.*<br><br>*The data underlying the results presented in the study are available from (include the name of the third party*	All relevant data are within the paper and its S1 Dataset files. Please inform the authors if data are being used.

and contact information or URL).<br>• This text is appropriate if the data are owned by a third party and authors do not have permission to share the data.<br><br>* typeset	
Additional data availability information:	

Manuscript

Click here to access/download;Manuscript;Manuscript - revision 2_20231229_clean.docx

Structural Validity of the Rosenberg Self-Esteem Scale in Patients with Schizophrenia in Indonesia

Muhammad Muslih [a, b], Min-Huey Chung [a, c*]

[a] School of Nursing, College of Nursing, Taipei Medical University, Taiwan

[b] School of Nursing, Faculty of Health Science, Universitas Muhammadiyah Malang, Indonesia

[c] Department of Nursing, Shuang Ho Hospital, Taipei Medical University, New Taipei City, Taiwan

Corresponding Author:

Min-Huey Chung, PhD, RN, FAAN

School of Nursing, College of Nursing, Taipei Medical University

No. 250, Wu-Xing Street, Taipei, Taiwan, 110, ROC.

Email address: minhuey300@tmu.edu.tw

Abstract

Background: The Rosenberg self-esteem scale (RSES) is a commonly employed instrument for measuring self-esteem in the general population and those with mental illness. However, confirmatory factor analyses (CFA) to determine the structural validity of the RSES for schizophrenia patients in Indonesia are limited.

Objectives: We examined the structural validity of the RSES as a measurement for patients with schizophrenia in Indonesia through confirmatory factor analyses (CFA), as well as assessing internal consistency and reliability.

Methods: The sample comprised 260 participants. Over two weeks, 30 subjects were added to investigate test-retest reliability. The structural validity analyzed was based on a CFA to determine the model fit. We used internal consistency (Cronbach's alpha) to evaluate the reliability evidence.

Results: Four different models were analyzed in this study. Considering the single-factor model (Model 1a), the overall fit criteria were inadequate. However, after some modification indices, all fit criteria were significantly adequate (Model 1b). The adequacy of all fit standards remained satisfactory when the two-factor model (Model 2) and hierarchical model (Model 3) were applied. The RSES had an alpha Cronbach coefficient of 0.75. While 0.89 and 0.88 for the positive and negative self-esteem subscale, respectively. Test-retest reliability yielded adequate results with an interclass correlation score ranging from 0.87 to 0.93.

Conclusions: The current investigation provided evidence supporting the structural validity, internal consistency, and reliability of the RSES, indicating that the RSES can be considered a valid and reliable measurement. A two-factor model of RSES was an appropriate model to measure self-esteem in our study. This finding suggests that the use of the RSES is beneficial and applicable in assessing levels of self-esteem in individuals diagnosed with schizophrenia in Indonesia.

Keywords: self-esteem; validity; reliability, schizophrenia

1. Introduction

Self-esteem is an overall individual evaluation or appraisal of the self [1] and how a person thinks of themselves. Self-esteem is "the degree to which a person values, approves of, or likes himself or herself." [2]. Self-esteem is a crucial component of mental health and general psychological well-being. It influences an individual's achievements and successes, social interactions, and ability to cope with environmental stressors [3, 4]. Individuals with high self-esteem believe they possess many positive qualities and attitudes toward themselves [5]. In summary, self-esteem is a pivotal psychological construct [6] that controls several facets of an individual's existence, encompassing mental well-being, accomplishments, interpersonal engagements, and coping abilities [3, 4].

There is a reciprocal relationship between self-esteem and mental illnesses [7]. A previous study found that self-esteem plays a vital role in developing diverse mental illnesses and social problems encompassing a range of internalizing issues, such as depression, suicidal tendencies, eating disorders, and anxiety, as well as externalizing problems, including violence and substance abuse [8]. Conversely, it has been hypothesized that mental illnesses can lead to low self-esteem as a significant consequence [9]. Consistent with prior research, low self-esteem has been found to heighten susceptibility to the onset of mental illness [10]. Eventually, individuals with mental illnesses are likely to have fluctuating self-esteem levels [11]. Therefore, drawing from the aforementioned explanation, we can conclude that self-esteem is considered a component of self-assessment, which influences mental health and vice versa.

The Rosenberg Self-Esteem Scale (RSES), which was developed by Rosenberg [12], is one of the most extensively used instruments for measuring self-esteem globally [13-17]. Researchers often use the RSES to measure self-esteem in the clinical population, such as eating disorders [18], anxiety, depression [7], attention and emotional disorder [19], schizophrenia and bipolar disorder [20]. Other studies have tested the RSES in specific people, such as ex-prisoners [21], drug users [22], and single mothers [23]. The RSES has been translated and adapted into a number of different languages,

including German [24], Dutch [25], Estonian [26], French [27], Portuguese [28], Spanish [29], Japanese [17], and Thai [14]; thus, making it applicable to participants from diverse samples or populations. It has been adapted across 53 nations with distinct ethnic groups and cultures [30]. This finding indicates that the RSES is widely used to measure self-esteem. Supporting this idea, a prior study suggests that the popularity of the RSES can be attributed to its brevity and simplicity, as it comprises only ten questions that can be completed within a short timeframe of 1 to 2 minutes [13].

Multiple countries, including Indonesia, have implemented the RSES to measure the self-esteem of college students and the general population [30]. Some previous studies have shown that self-esteem has been associated with schizophrenia [31-33]. For example, there was a significant correlation between a decrease in the intensity of adverse symptoms and an enhancement in self-esteem, and conversely [34]. However, psychometric analyses, including structural validity and reliability, in patients diagnosed with schizophrenia were not explicitly addressed in previous studies [30, 35].

In a prior study, the factor structure of the RSES was examined using psychometric tests, and it focused on adolescents [36], as they were the original target population of this scale. It has also been tested in adults [37] and the general population [24]. Nevertheless, there is no available evidence supporting the utilization of RSES among individuals diagnosed with schizophrenia in Indonesia. Hence, evaluating the RSES in individuals diagnosed with schizophrenia is imperative to ascertain its psychometric analysis. This study aimed to assess the structural validity of the RSES as a measurement for patients with schizophrenia in Indonesia through confirmatory factor analyses (CFA), as well as assessing internal consistency and reliability.

2. Methods

2.1 Participants

This is an instrumental questionnaire validation study. Two psychiatric hospitals and one psychiatric rehabilitation center in East Java, Indonesia, were visited to obtain the required data. We

distributed the questionnaire from August 2018 to February 2019. Participants were recruited using the convenience sampling technique. The following requirements had to be met for someone to be included: (a) they had been diagnosed with schizophrenia; (b) aged ≥ 20 years; (c) hospitalized in a psychiatric ward; and (d) able to speak, read, and write Indonesian. The Mini-Mental State Examination (MMSE) was utilized to screen out participants with cognitive impairment (i.e., MMSE scores < 24).

The size of 200 participants required in this study is acceptable based on recommendations from prior studies [38, 39]. In this study, data was missing from four questionnaires because they were not accurately completed, and 21 participants were excluded because their MMSE score was less than 24. Considering the response rate of 20%, the final sample comprised 260 participants. In addition, we recruited an additional 30 individuals to investigate test-retest reliability over two weeks.

2.2 Instruments

The study incorporates many socio-demographic data, including age as a continuous variable. The remaining variables as categorical variables, namely gender (male; female), marital status (single; married; divorced, or widowed), employment status (employed; unemployed), source of income (personal income; family support; personal and family support), education (elementary; junior; high school; university/ college), previous hospitalization (yes; no), and onset of illness (<1 year; 1-5 years; >5 years).

The RSES is not licensed and is available for public use. Information about the scale can easily be gathered, and permission to use this resource can be sought at https://socy.umd.edu/about-us/rosenberg-self-esteem-scale. The scale consists of 10 items evaluated on a 4-point Likert scale from 1 (strongly disagree) to 4 (strongly agree). Scores vary from 10 to 40, with higher scores indicating a more positive self-esteem appraisal. To measure the reverse score, five questions are worded positively (items 1, 3, 4, 7, and 10), while five are worded negatively (items 2, 5, 6, 8, and 9).

The validity and reliability evidence of the RSES were assessed in prior studies, and the obtained results are presented herein. Concerning the evaluation of construct validity, prior studies have demonstrated that the RSES yielded an excellent model fit [21, 40]. According to a previous study [41] it is suggested to ensure that the Cronbach alpha criteria for each sub-scale is ≥ 0.70. The Cronbach's alpha coefficients for the positive and negative self-esteem subscales were determined to be 0.96 and 0.98, respectively [21]. A prior study conducted on individuals who are native English speakers has also yielded Cronbach alpha coefficients of 0.87 and 0.75 for the subscales measuring positive and negative self-esteem, respectively [17].

2.3 Translation Procedure

Due to their specificity and straightforwardness, we adhered to the parameters suggested by a previous study [42]. Initially, the original questionnaire was translated into Indonesian by two translators, a psychiatrist and a professional translator whose native language was Indonesian, with the author's approval. Both were bilingual and English-proficient. Second, we compared the two translated versions and created a new draft by combining the terminology and phrases supplied by the two translators in the previous step. Thirdly, the information was back-translated by two more independent translators with the same credentials and qualities as the first translators. Fourth, we compared the original questionnaire with the two back-translations of the questionnaire from the third phase. Considering the distance and time variations, we communicated with all four translators by email at this stage. Fifth, 30 volunteers were selected from a psychiatric hospital to evaluate the clarity of the questionnaire's instructions, items, and response format. In addition, we asked two professionals (a psychiatrist and a psychologist) for revisions and ideas. In the final phase, the full Indonesian version of the RSES scale was administered to the study sample and tested the evidence of validity and reliability (See Figure 1).

2.4 Statistical Analysis

This study utilized SPSS and AMOS version 23.0 software (IBM; Armonk, New York, USA). All statistical significance was indicated by a p-value < 0.05.

Descriptive analysis

Descriptive statistics were used to present the demographic characteristics of the study. The proportion of participants who obtained minimum and maximum scores is defined as floor and ceiling effect, respectively. Continuous variables are presented as means and standard deviations, whereas categorical variables are expressed as frequencies and percentages. The quantitative characteristics of the RSES were computed as mean, standard deviation (SD), skewness, and kurtosis. A skewness value between −1 and 1 was considered adequate [43]. The degree of vertical spread in the mean distribution corresponded to the kurtosis. The normality was assumed if the kurtosis value was less than 2.5 times the standard error [44].

Structural validity.

Structural validity pertains to the extent to which the scores of a Patient-Reported Outcome Measure (PROM) accurately represent the underlying structure of the construct being measured [41]. Assessment of structural validity is typically conducted through the use of CFA [41, 45]. A CFA was carried out to assess how well the RSES model fits the data. The following fit indices were utilized during the evaluation process: X^2/df, the comparative fit index (CFI), incremental fit index (IFI), the Tucker–Lewis index (TLI), goodness-of-fit index (GFI), adjusted goodness-of-fit index (AGFI), the standardized root mean square residual (SRMR), and the root mean square error of approximation (RMSEA). A chi-square with a degree of freedom ratio of less than 5.0 indicated that the model was a good fit [46]. An acceptable model fit was characterized by a GFI greater than 0.80 [47] and an AGFI of 0.80 to 0.90 [48-52]. When the CFI, IFI, and TLI values were all greater than 0.90 [53], the SRMR value was less than 0.08 [53, 54], and the RMSEA value was less than 0.10 [47], the model fit was deemed to be satisfactory. Akaike Information Criterion (AIC) and Bayesian Information Criterion

(BIC) were used to evaluate the alternative model, with lower AIC and BIC values indicating the best model fit [55, 56]. The average variance extracted (AVE) are an integral component of the comprehensive construct validity assessment., and an AVE score greater than 0.50 indicates an adequate result [58].

Internal consistency.

Internal consistency refers to “the extent of interrelatedness among the items and is commonly evaluated using Cronbach's alpha” [62]. A Cronbach's alpha equal to or better than 0.70 demonstrates an adequate internal consistency [59, 60]. The composite reliability (CR) was used to measure a robust internal consistency. The values of CR greater than 0.70 indicated a significant result [57].

Reliability.

Interclass correlation coefficient (ICC) was used to evaluate the reliability [41]. The ICC score between 0.75 and 0.90 indicated satisfactory reliability and consistency between two-time measurements, and a score greater than 0.90 revealed excellent reliability [61].

2.5 Ethical Approval

The Ethics Research Committee approved this research of the University of Muhammadiyah Malang on July 19, 2018 (approval number: E.5.a/239/KEPK- UMM/VII/2018).

3. Results

Table 1 presents the clinical demographics of the study. The mean age of the 260 participants was 38.13 (SD = 9.56). Most participants were men (169, 65%) and single (139, 53.5%). Most were unemployed (173, 66.5%) and received financial support from their families (196, 75.4%). For 127 participants (48.8%), the highest educational level was senior high school. In total, 220 participants

(84.6%) had previously been hospitalized, and the illness duration to onset was >5 years for 142 participants (54.6%).

Table 2 displays the descriptive statistics, inter-item correlation, and item-total correlation of the RSES. Item 8 and item 3 received the highest and lowest mean scores of 2.57 (SD = 0.87) and 1.92 (SD = 0.81), respectively. The skewness score ranged from 0.39 to 0.90 for the total RSES items, and the kurtosis score ranged from 0.74 to 0.75. The item-total correlation varied from 0.47 to 0.6. Floor and ceiling effect was found at 5.80% - 54.20%, respectively (Table 4).

3.1 Structural validity.

The goodness of indices for all alternative models is shown in Table 3. Considering the single-factor or uni-dimensional model (M1a), the overall fit criteria were inadequate (See Figure 2). However, after some modification indices (See Figure 3), all fit criteria were significantly adequate (M1b). The adequacy of all fit criteria remained satisfactory when the two-factor model (M2) and hierarchical model (M3) were applied (See Figure 4 and Figure 5). The AVE values were 0.69 and 0.68, and the square roots of the AVE were 0.83 and 0.82, indicating that each measured variable was significant (Table 4).

3.2 Internal consistency and reliability evidence.

The RSES had an alpha coefficient of 0.75, according to Cronbach's method. The results of Cronbach's alpha, which measures internal consistency, came in at 0.89 and 0.88 for each subscale (factor), indicating acceptable internal consistency. As presented in Table 4, the CR was calculated for positive and negative factors, and the values were 0.92 and 0.91, respectively. Test-retest reliability exhibited satisfactory results, with an ICC between 0.87 and 0.93 (Table 4).

4. Discussion

This study aimed to establish the structural validity of the RSES as well as assess internal consistency and reliability. We included patients with schizophrenia in our study, which was not the

case in the previous study [30]. The sample size of our study was adequate to perform factor analysis. In addition, the structural validity of the RSES was demonstrated through CFA, an approach that has not been conducted in prior studies in Indonesia.

Through the CFA, we examine four models of structural validity in our study, which are; the single-factor or uni-dimensional model (M1a), the single-factor with correlated error (M1b), the two-factor model (M2), and the hierarchical model (M3). The findings from Model 1a, suggest that there is insufficient data to support the acceptance of a single factor. Where every model fit criterion failed to meet the required levels. A notable distinction was observed while implementing the correlated error in Model 1b, demonstrating an enhancement in the good fit criteria. This adjustment was done on the negatively worded item. This finding aligns with some prior studies that have examined the presence of method effects related to negative items on the RSES [64-66]. While some of the model fit criteria are met, it is unfortunate that this model also fell outside of other criteria. The BIC value is significantly higher compared to models 2 and 3. Following a previous study, the difference in BIC score greater than 10 provides strong evidence of the model [67]. If the objective is to achieve a goodness of fit, the BIC is the preferred option. Therefore, using BIC is more advantageous when selecting an accurate model [68].

The adequate fit indexes were also obtained in Models 2 and 3. The two-factor and hierarchical models exhibit comparable model fit in their respective analyses. Based on the findings mentioned above, it is suggested that the RSES can be characterized as two factors, which are positive and negative self-esteem. A previous study also referred to these two factors as positive and negative self-esteem [69]. The influence of wording effect on scale items may result in or contribute to a two-factor model. The wording item effect, then further related to the method effect has been observed in earlier studies [37, 70], which suggests the presence of a two-factor of RSES [70]. In summary, our finding indicates that the RSES scale has an acceptable model fit with two factors, Similar findings were also demonstrated in a previous study that a two-factor model was deemed to have an adequate model fit

[21, 36, 71, 72]. Based on our finding, we can conclude that the RSES with is a two-factor model was a valid instrument for people with schizophrenia in Indonesia. Acknowledging the necessity of reassessing the utilization of the RSES and its theoretical foundations in administering the scale to target populations is essential.

The total score on the Indonesian version of the RSES had a Cronbach's alpha coefficient of 0.75. In contrast, the positive and negative self-esteem subscale had a coefficient of 0.89, and 0.88 respectively. In line with this finding, a previous investigation found satisfactory levels of internal consistency, as measured by a Cronbach's alpha coefficient that ranged from 0.81 to 0.91 [13, 14, 25, 30]. Our result was consistent with a previous study by Torrey, Mueser, McHugo, and Drake [63], which was conducted in individuals with severe mental illnesses, not specific only to patients with schizophrenia, and which reported strong internal consistency. To demonstrate more robust internal consistency, composite reliability also exhibits favorable outcomes.

In addition, we looked at the test-retest reliability of the RSES. The ICC values generated were 0.93 and 0.87 for the positive and negative self-esteem respectively. The ICC results were adequate, indicating the stability of each factor of the RSES. Additionally, it was observed that there was a high correlation coefficient between the test-retest reliability and Cronbach's alpha. In conclusion, the findings of this study provide evidence supporting the robust reliability of the RSES as a reliable instrument for assessing self-esteem in individuals diagnosed with schizophrenia in Indonesia.

The present study also has limitations. Because our study sample included only patients with schizophrenia, these findings cannot be extrapolated to populations with other mental illnesses. Moreover, most of our study's participants are men, leading to a more prominent interpretation of item scores among this group. Future studies should aim to expand the participant pool by including individuals diagnosed with a diverse range of mental illnesses while also ensuring a balanced representation of both genders. In addition to the limitations mentioned above, it is essential to note that our study produced favorable evidence regarding structural validity, internal consistency, and

reliability. Hence establishing the RSES as a valid and reliable questionnaire appropriate for implementation within the tested sample group.

5. Conclusion

The current investigation provided evidence supporting the structural validity, internal consistency, and reliability of the RSES, indicating that the RSES can be considered a valid and reliable measurement. A two-factor model of RSES was an appropriate model to measure self-esteem in our study. This finding suggests that the use of the RSES is beneficial and applicable in assessing levels of self-esteem in individuals diagnosed with schizophrenia in Indonesia. Nevertheless, further research is needed to understand better the characteristics of the method factors for different populations. As previously stated, positive and negative item wording is still a major consideration in psychometric analysis.

Supporting information

S1 Dataset

Data availability

All relevant data are within the paper and its S1 Dataset files. Please inform the authors if data are being used.

Conflict of Interest

The authors declare that they have no conflicts of interest.

Funding

The authors did not receive any funding for this study.

Acknowledgment

We would like to acknowledge Wallace Academic Editing Company for editing this manuscript.

Authors' Contributions

M.M collected, analyzed, and interpreted the data and drafted the manuscript. M.H.C studied and analyzed the data and co-drafted the manuscript.

References

1. Cast AD, Burke PJ. A theory of self-esteem. Social forces. 2002;80(3):1041-68.

2. Blascovich J, Tomaka J. Measures of self-esteem. Measures of personality and social psychological attitudes. 1991;1:115-60.

3. Battle J. Promoting self-esteem, achievement and well being: An effective instructional curriculum for all levels: James Battle and Associates; 1994.

4. Huang C, Dong N. Factor structures of the Rosenberg self-esteem scale. European Journal of Psychological Assessment. 2012.

5. Bonfine N. Stigma self concept and stigma resistance among individuals with mental illness [Disertation]. Ann Arbor: Kent State University; 2013.

6. von Soest T, Wichstrøm L, Kvalem IL. The development of global and domain-specific self-esteem from age 13 to 31. Journal of Personality and Social Psychology. 2016;110(4):592.

7. van Tuijl LA, de Jong PJ, Sportel BE, de Hullu E, Nauta MH. Implicit and explicit self-esteem and their reciprocal relationship with symptoms of depression and social anxiety: A longitudinal study in adolescents. Journal of behavior therapy and experimental psychiatry. 2014;45(1):113-21.

8. Mann M, Hosman CM, Schaalma HP, de Vries NK. Self-esteem in a broad-spectrum approach for mental health promotion. Health Educ Res. 2004;19(4):357-72. Epub 2004/06/17. doi: 10.1093/her/cyg041. PubMed PMID: 15199011.

9. Link BG, Struening EL, Neese-Todd S, Asmussen S, Phelan JC. Stigma as a barrier to recovery: The consequences of stigma for the self-esteem of people with mental illnesses. Psychiatric services. 2001;52(12):1621-6.

10. Silverstone PH, Salsali M. Low self-esteem and psychiatric patients: Part I – The relationship between low self-esteem and psychiatric diagnosis. Annals of General Hospital Psychiatry. 2003;2(1):2. doi: 10.1186/1475-2832-2-2.

11. Romm KL, Rossberg JI, Hansen CF, Haug E, Andreassen OA, Melle I. Self-esteem is associated with premorbid adjustment and positive psychotic symptoms in early psychosis. BMC psychiatry. 2011;11(1):1-8.

12. Rosenberg M. Society and the Adolescent Self-Image. Revised edition. Middletown, CT: Wesleyan University Press; 1989.

13. Sinclair SJ, Blais MA, Gansler DA, Sandberg E, Bistis K, LoCicero A. Psychometric properties of the Rosenberg Self-Esteem Scale: Overall and across demographic groups living within the United States. Evaluation & the health professions. 2010;33(1):56-80.

14. Tinakon W, Nahathai W. A comparison of reliability and construct validity between the original and revised versions of the Rosenberg Self-Esteem Scale. Psychiatry investigation. 2012;9(1):54.

15. Martin CR, Thompson DR, Chan DS. An examination of the psychometric properties of the Rosenberg Self-Esteem Scale (RSES) in Chinese acute coronary syndrome (ACS) patients. Psychology, health & medicine. 2006;11(4):507-21.

16. Martín-Albo J, Núñez JL, Navarro JG, Grijalvo F. The Rosenberg Self-Esteem Scale: translation and validation in university students. The Spanish journal of psychology. 2007;10(2):458-67.

17. Mimura C, Griffiths P. A Japanese version of the Rosenberg Self-Esteem Scale: Translation and equivalence assessment. Journal of Psychosomatic Research. 2007;62(5):589-94.

18. Salerno L, Ingoglia S, Coco GL. Competing factor structures of the Rosenberg Self-Esteem Scale (RSES) and its measurement invariance across clinical and non-clinical samples. Personality and Individual Differences. 2017;113:13-9.

19. Henriksen IO, Ranøyen I, Indredavik MS, Stenseng F. The role of self-esteem in the development of psychiatric problems: a three-year prospective study in a clinical sample of adolescents. Child and adolescent psychiatry and mental health. 2017;11:1-9.

20. Oliveira SE, Esteves F, Carvalho H. Clinical profiles of stigma experiences, self-esteem and social relationships among people with schizophrenia, depressive, and bipolar disorders. Psychiatry research. 2015;229(1-2):167-73.

21. Boduszek D, Hyland P, Dhingra K, Mallett J. The factor structure and composite reliability of the Rosenberg Self-Esteem Scale among ex-prisoners. Personality and individual differences. 2013;55(8):877-81.

22. Wang J, Siegal HA, Falck RS, Carlson RG. Factorial structure of Rosenberg's Self-Esteem Scale among crack-cocaine drug users. Structural Equation Modeling. 2001;8(2):275-86.

23. Hatcher J, Hall LA. Psychometric properties of the Rosenberg self-esteem scale in African American single mothers. Issues in Mental Health Nursing. 2009;30(2):70-7.

24. Roth M, Decker O, Herzberg PY, Brähler E. Dimensionality and norms of the Rosenberg Self-Esteem Scale in a German general population sample. European Journal of Psychological Assessment. 2008;24(3):190-7.

25. Franck E, De Raedt R, Barbez C, Rosseel Y. Psychometric properties of the Dutch Rosenberg self-esteem scale. Psychologica Belgica. 2008;48(1):25-35.

26. Pullmann H, Allik J. The Rosenberg Self-Esteem Scale: its dimensionality, stability and personality correlates in Estonian. Personality and Individual differences. 2000;28(4):701-15.

27. Vallieres EF, Vallerand RJ. Traduction et validation canadienne- française de l'échelle de l'estime de soi de Rosenberg. International journal of psychology. 1990;25(2):305-16.

28. Vasconcelos-Raposo J, Fernandes HM, Teixeira CM, Bertelli R. Factorial validity and invariance of the Rosenberg Self-Esteem Scale among Portuguese youngsters. Social Indicators Research. 2012;105(3):483-98.

29. Mayordomo T, Gutierrez M, Sales A. Adapting and validating the Rosenberg Self-Esteem Scale for elderly Spanish population. International Psychogeriatrics. 2020;32(2):183-90.

30. Schmitt DP, Allik J. Simultaneous administration of the Rosenberg Self-Esteem Scale in 53 nations: exploring the universal and culture-specific features of global self-esteem. Journal of personality and social psychology. 2005;89(4):623.

31. Moritz S, Veckenstedt R, Randjbar S, Vitzthum F, Karow A, Lincoln TM. Course and determinants of self- esteem in people diagnosed with schizophrenia during psychiatric treatment. Psychosis. 2010;2(2):144-53.

32. Haug E, Øie MG, Andreassen OA, Bratlien U, Romm KL, Møller P, et al. The association between anomalous self-experiences, self-esteem and depressive symptoms in first episode schizophrenia. Frontiers in human neuroscience. 2016;10:557.

33. Kim EY, Jang MH. The Mediating Effects of Self-Esteem and Resilience on the Relationship Between Internalized Stigma and Quality of Life in People with Schizophrenia. Asian Nursing Research. 2019;13(4):257-63. doi: https://doi.org/10.1016/j.anr.2019.09.004.

34. Jones RM, Hansen L, Moskvina V, Kingdon D, Turkington D. The relationship between self- esteem and psychotic symptoms in schizophrenia: A longitudinal study. Psychosis. 2010;2(3):218-26.

35. Pardede JA, Keliat BA, Wardani IY. The Symptoms of Low Self-Esteem Decline after Being Given Acceptance and Commitment Therapy. Adv Practice Nurs. 2020;5(170):10.37421.

36. Marsh HW, Scalas LF, Nagengast B. Longitudinal tests of competing factor structures for the Rosenberg Self-Esteem Scale: traits, ephemeral artifacts, and stable response styles. Psychological assessment. 2010;22(2):366.

37. Gana K, Saada Y, Bailly N, Joulain M, Hervé C, Alaphilippe D. Longitudinal factorial invariance of the Rosenberg Self-Esteem Scale: Determining the nature of method effects due to item wording. Journal of Research in Personality. 2013;47(4):406-16.

38. Ferrando Piera PJ, Lorenzo Seva U, Hernández Dorado A, Muñiz Fernández J. Decálogo para el Análisis Factorial de los Ítems de un Test. Psicothema. 2022.

39. Lloret-Segura S, Ferreres-Traver A, Hernandez-Baeza A, Tomas-Marco I. Exploratory item factor analysis: A practical guide revised and updated. Anales de Psicología. 2014;30(3):1151-69.

40. Xu ML, Leung SO. Effects of varying numbers of Likert scale points on factor structure of the Rosenberg Self- Esteem Scale. Asian Journal of Social Psychology. 2018;21(3):119-28.

41. Prinsen CA, Mokkink LB, Bouter LM, Alonso J, Patrick DL, De Vet HC, et al. COSMIN guideline for systematic reviews of patient-reported outcome measures. Quality of life research. 2018;27:1147-57.

42. Sousa VD, Rojjanasrirat W. Translation, adaptation and validation of instruments or scales for use in cross-cultural health care research: a clear and user-friendly guideline. Journal of Evaluation Clinical Practice. 2011;17:268-74. Epub 2010/09/30. doi: 10.1111/j.1365-2753.2010.01434.x. PubMed PMID: 20874835.

43. Indrayan A, Malhotra RK. Medical biostatistics: Chapman and Hall/CRC; 2017.

44. Morgan GA, Griego OV. Easy use and interpretation of SPSS for Windows: Answering research questions with statistics: Psychology Press; 1998.

45. Polit DF. Assessing measurement in health: Beyond reliability and validity. International journal of nursing studies. 2015;52(11):1746-53.

46. Marsh HW, Hocevar D. Application of confirmatory factor analysis to the study of self-concept: First-and higher order factor models and their invariance across groups. Psychological bulletin. 1985;97(3):562.

47. Meyers LS, Gamst G, Guarino AJ. Applied Multivariate Research: Design and Interpretation: SAGE Publications; 2006.

48. Bentler PM. Comparative fit indexes in structural models. Psychological bulletin. 1990;107(2):238.

49. Marsh HW, Balla JR, McDonald RP. Goodness-of-fit indexes in confirmatory factor analysis: The effect of sample size. Psychological bulletin. 1988;103(3):391.

50. Cole DA. Utility of confirmatory factor analysis in test validation research. Journal of consulting and clinical psychology. 1987;55(4):584.

51. Floyd FJ, Widaman KF. Factor analysis in the development and refinement of clinical assessment instruments. Psychological assessment. 1995;7(3):286.

52. Greenspoon PJ, Saklofske DH. Confirmatory factor analysis of the multidimensional students' life satisfaction scale. Personality and Individual Differences. 1998;25(5):965-71.

53. Hu Lt, Bentler PM. Cutoff criteria for fit indexes in covariance structure analysis: Conventional criteria versus new alternatives. Structural equation modeling: a multidisciplinary journal. 1999;6(1):1-55.

54. Hoyle RH. Structural equation modeling: Concepts, issues, and applications: Sage; 1995.

55. Akaike H. A new look at the statistical model identification. IEEE transactions on automatic control. 1974;19(6):716-23.

56. Schwarz G. Estimating the dimension of a model. The annals of statistics. 1978:461-4.

57. Grewal R, Cote JA, Baumgartner H. Multicollinearity and measurement error in structural equation models: Implications for theory testing. Marketing science. 2004;23(4):519-29.

58. Hair J, Anderson R, Black B, Babin B. Multivariate Data Analysis: Pearson Education; 2016.

59. Polit DF, Beck CT. Essentials of nursing research: Appraising evidence for nursing practice: Lippincott Williams & Wilkins; 2010.

60. Tavakol M, Dennick R. Making sense of Cronbach's alpha. International journal of medical education. 2011;2:53.

61. Koo TK, Li MY. A guideline of selecting and reporting intraclass correlation coefficients for reliability research. Journal of chiropractic medicine. 2016;15(2):155-63.

62. Mokkink LB, Terwee CB, Patrick DL, Alonso J, Stratford PW, Knol DL, et al. The COSMIN study reached international consensus on taxonomy, terminology, and definitions of measurement properties for health-related patient-reported outcomes. Journal of clinical epidemiology. 2010;63(7):737-45.

63. Torrey WC, Mueser KT, McHugo GH, Drake RE. Self-esteem as an outcome measure in studies of vocational rehabilitation for adults with severe mental illness. Psychiatric Services. 2000;51(2):229-33.

64. Mullen SP, Gothe NP, McAuley E. Evaluation of the factor structure of the Rosenberg Self-Esteem Scale in older adults. Personality and individual differences. 2013;54(2):153-7.

65. Aluja A, Rolland J-P, García LF, Rossier J. Dimensionality of the Rosenberg Self-Esteem Scale and its relationships with the three-and the five-factor personality models. Journal of Personality Assessment. 2007;88(2):246-9.

66. Makhubela M, Mashegoane S. Psychological validation of the Rosenberg Self-Esteem Scale (RSES) in South Africa: Method effects and dimensionality in black African and white university students. Journal of Psychology in Africa. 2017;27(3):277-81.

67. Bauldry S. Structural Equation Modeling. In: Wright JD, editor. International Encyclopedia of the Social & Behavioral Sciences (Second Edition). Oxford: Elsevier; 2015. p. 615-20.

68. Chakrabarti A, Ghosh JK. AIC, BIC and recent advances in model selection. Philosophy of statistics. 2011:583-605.

69. Ang RP, Neubronner M, Oh S-A, Leong V. Dimensionality of Rosenberg's self-esteem scale among normal-technical stream students in Singapore. Current Psychology. 2006;25(2):120-31.

70. Wu C-H. An examination of the wording effect in the Rosenberg Self-Esteem Scale among culturally Chinese people. Journal of Social Psychology. 2008;148(5).

71. Quilty LC, Oakman JM, Risko E. Correlates of the Rosenberg self-esteem scale method effects. Structural Equation Modeling. 2006;13(1):99-117.

72. Boduszek D, Shevlin M, Mallett J, Hyland P, O'Kane D. Dimensionality and construct validity of the Rosenberg self- esteem scale within a sample of recidivistic prisoners. Journal of Criminal Psychology. 2012;2(1):19-25.

73. Reise SP, Kim DS, Mansolf M, Widaman KF. Is the bifactor model a better model or is it just better at modeling implausible responses? Application of iteratively reweighted least squares to the Rosenberg Self-Esteem Scale. Multivariate behavioral research. 2016;51(6):818-38.

Table 1. Demographic Characteristics of the Study (*n* = 260)

Characteristics	Participants (*n* = 260)	
	**Mean (SD)**	**n (%)**
Age	38.13 (9.56)	
Gender		
Male		169 (65.00)
Female		91 (35.50)
Marital status		
Single		139 (53.46)
Married		81 (31.15)
Divorce or widowed		40 (15.39)
Employment status		
Employed		87 (33.50)
Unemployed		173 (66.50)
Source of income		
Personal income		30 (11.50)
Family support		196 (75.40)
Personal and family		34 (13.10)
Education		
Elementary school		63 (24.23)
Junior high school		44 (16.92)
Senior high school		127 (48.85)
University/college		26 (10.00)
Previous hospitalization		
Yes		220 (84.60)
No		40 (15.40)
Onset of illness		
<1 year		75 (28.85)
1–5 years		43 (16.54)
>5 years		142 (54.61)

*missing data = 4; **MMSE score < 24 = 21
SD = standard deviation; MMSE = Mini-Mental State Examination.

Table 2. Descriptive Statistics, Interitem, and Item-Total Correlation of the RSES Items (n = 260)

Items		Inter-item correlation (n = 260)									
		1	2	3	4	5	6	7	8	9	10
RSES 1	On the whole, I am satisfied with myself.	1									
RSES 2	At times, I think I am no good at all.	−0.11	1								
RSES 3	I feel that I have a number of good qualities.	0.56	−0.12	1							
RSES 4	I am able to do things as well as most other people.	0.51	−0.04	0.63	1						
RSES 5	I feel I do not have much to be proud of.	−0.03	0.48	−0.13	−0.11	1					
RSES 6	I certainly feel useless at times.	−0.00	0.62	−0.04	−0.01	0.48	1				
RSES 7	I feel that I'm a person of worth, at least on an equal plane with others.	0.52	−0.01	0.64	0.86	−0.07	0.06	1			
RSES 8	I wish I could have more respect for myself.	−0.19	0.71	−0.22	−0.15	0.43	0.50	−0.15	1		
RSES 9	All in all, I am inclined to feel that I am a failure.	−0.1	0.85	−0.13	−0.09	0.56	0.63	−0.05	0.71	1	
RSES 10	I take a positive attitude toward myself.	0.55	0.01	0.58	0.63	−0.01	−0.03	0.64	−0.08	−0.04	1
Item-total correlation		**0.49**	**0.61**	**0.50**	**0.58**	**0.47**	**0.59**	**0.62**	**0.46**	**0.61**	**0.59**
Mean		2.05	2.39	1.92	1.98	2.32	2.30	2.02	2.57	2.31	1.97
SD		0.94	0.86	0.81	0.84	0.86	0.91	0.83	0.87	0.83	0.86
Skewness		0.58	−0.02	0.90	0.69	0.32	0.21	0.59	−0.39	0.28	0.72
Kurtosis		−0.55	−0.68	0.75	0.05	−0.47	−0.74	−0.08	−0.53	−0.40	0.00

SD = standard deviation

Table 3. Goodness of fit indexes of the RSES Indonesian version.

Item	Model 1	Model 2	Model 3	Model 4
X^2/df	24.50	2.96	2.93	2.93
GFI	0.55	0.93	0.93	0.93
AGFI	0.30	0.88	0.88	0.88
CFI	0.48	0.97	0.96	0.96
TLI	0.33	0.94	0.95	0.95
IFI	0.48	0.97	0.96	0.96
SRMR	0.19	0.03	0.04	0.04
RMSEA	0.30	0.08	0.08	0.08
AIC	897.634	133.992	141.497	141.497
BIC	968.848	240.812	216.27	216.27

df = degree of freedom GFI = goodness of fit index; AGFI = adjusted goodness of fit index; CFI = comparative fit index; TLI = Tucker-Lewis index; IFI = incremental fit index; SRMR = standardized root mean square residual; RMSEA = root mean square error of approximation; AIC = Akaike information criterion; BIC = Bayesian information criterion

Table 4. Reliability Analysis for the Indonesian Adaptation of the Rosenberg Self-Esteem Scale (*n* = 260)

Items	% floor effect	% ceiling effect	Cronbach's alpha	Test-retest reliability		AVE	CR
				ICC	95% CI		
Positive self-esteem			0.89	0.93	0.88–0.96	0.69	0.92
RSES 1	9.20	39.60					
RSES 3	6.50	54.20					
RSES 4	6.50	48.80					
RSES 7	5.80	48.80					
RSES 10	6.90	46.90					
Negative self-esteem			0.88	0.87	0.78–0.93	0.68	0.91
RSES 2	8.50	38.10					
RSES 5	10.40	47.30					
RSES 6	10.40	40.00					
RSES 8	10.80	50.00					
RSES 9	8.50	47.70					

CI = confidence interval, ICC = intraclass correlation coefficient, AVE = average variance extracted, CR = composite reliability,

Figure
Click here to access/download;Figure;Figure new..pdf

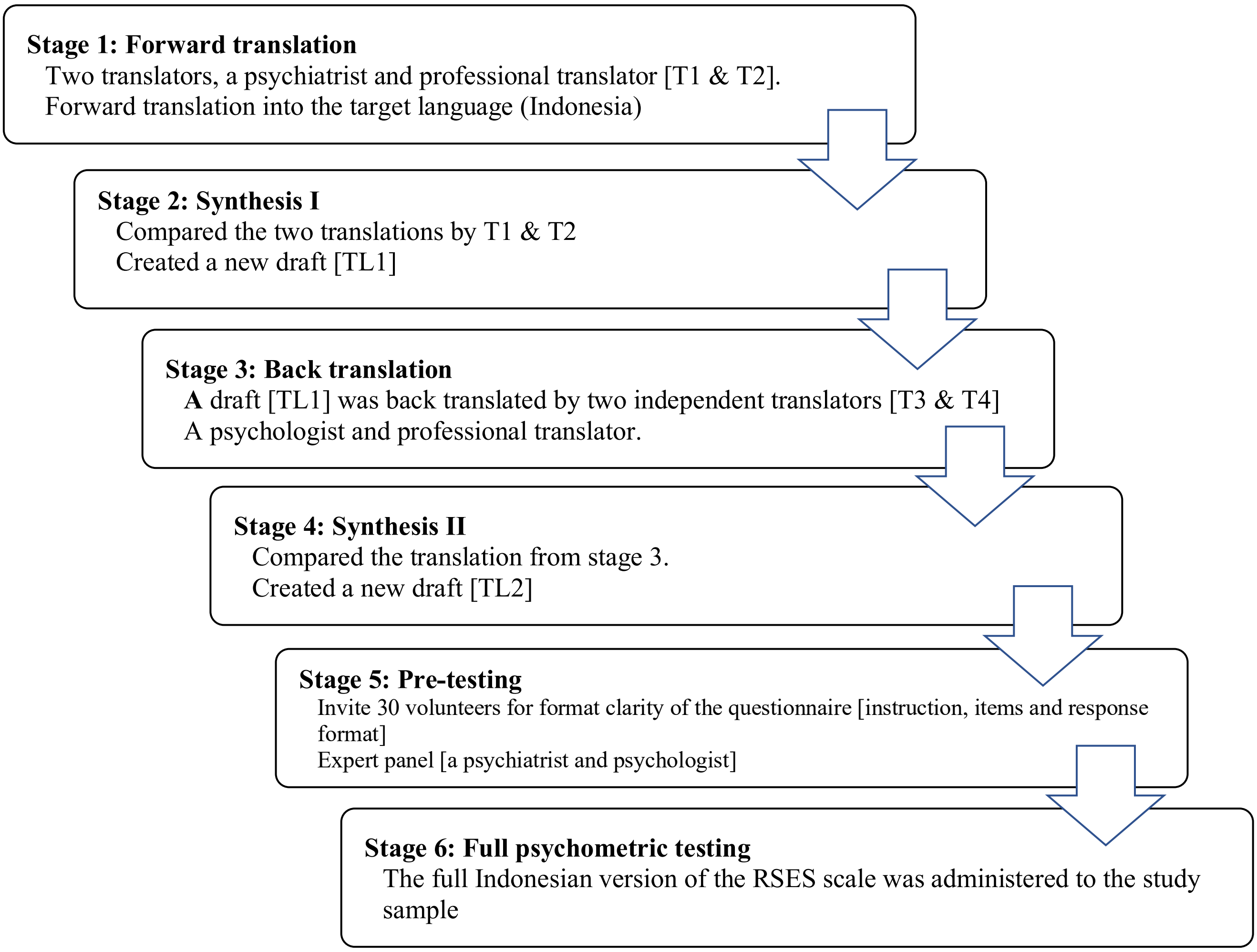

Figure 1. Flow diagram of the translational process of RSES.

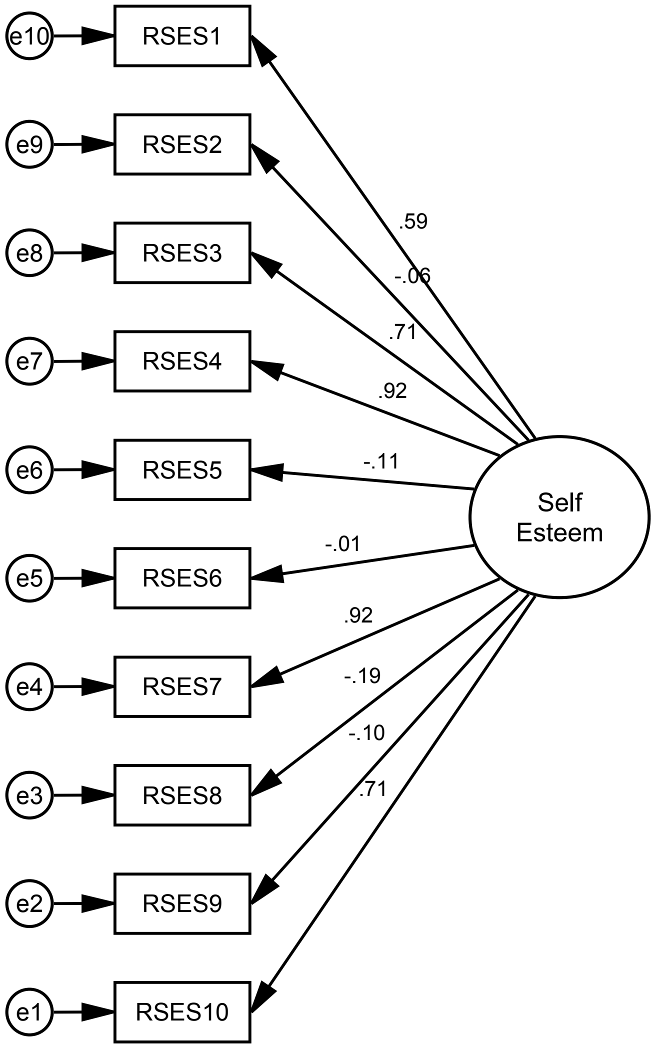

Figure 2. Single-factor model of RSES-Indonesian version (Model 1a)

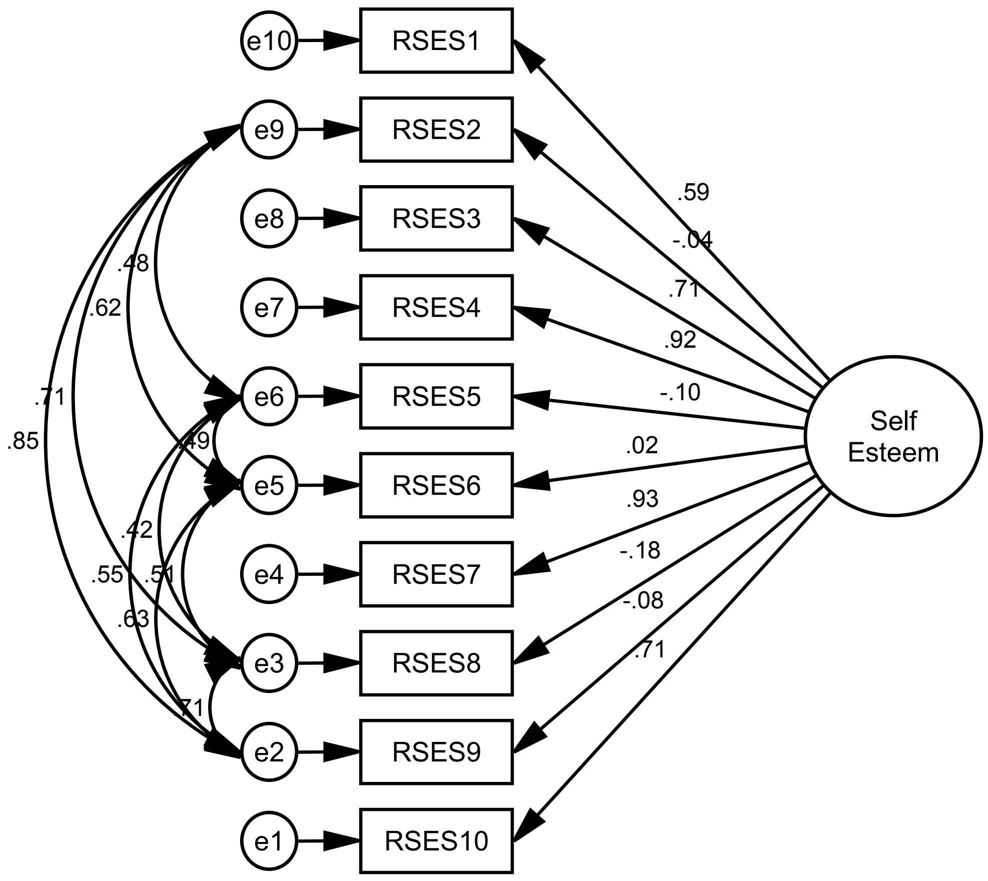

Figure 3. Single-factor model with correlated error (Model 1b)

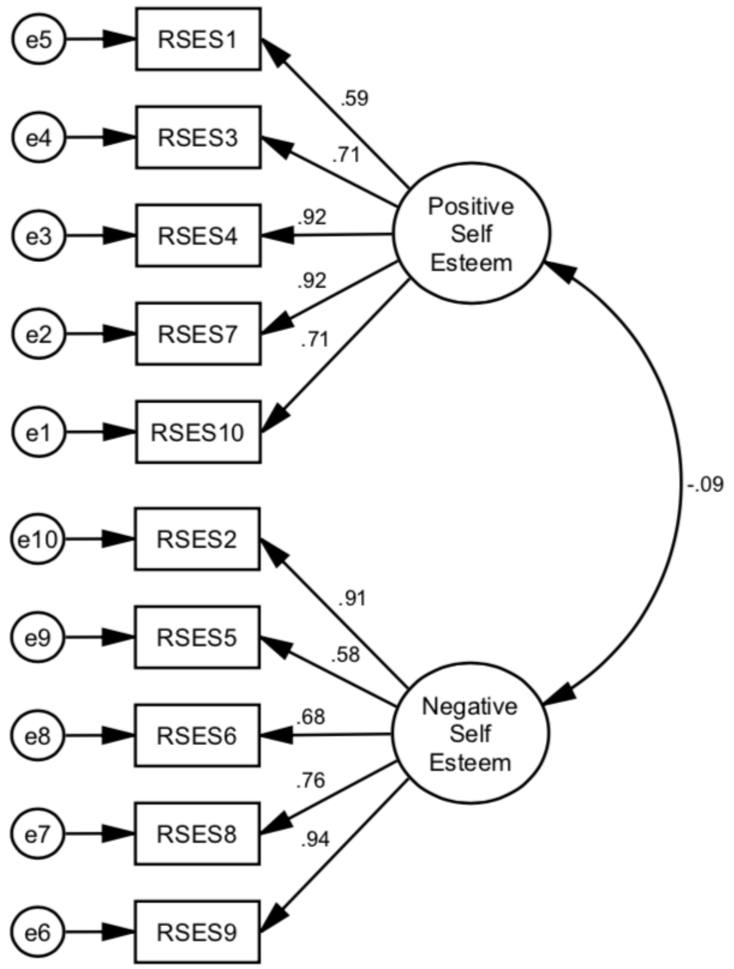

Figure 4. Two-factor model of RSES-Indonesian version (Model 2)

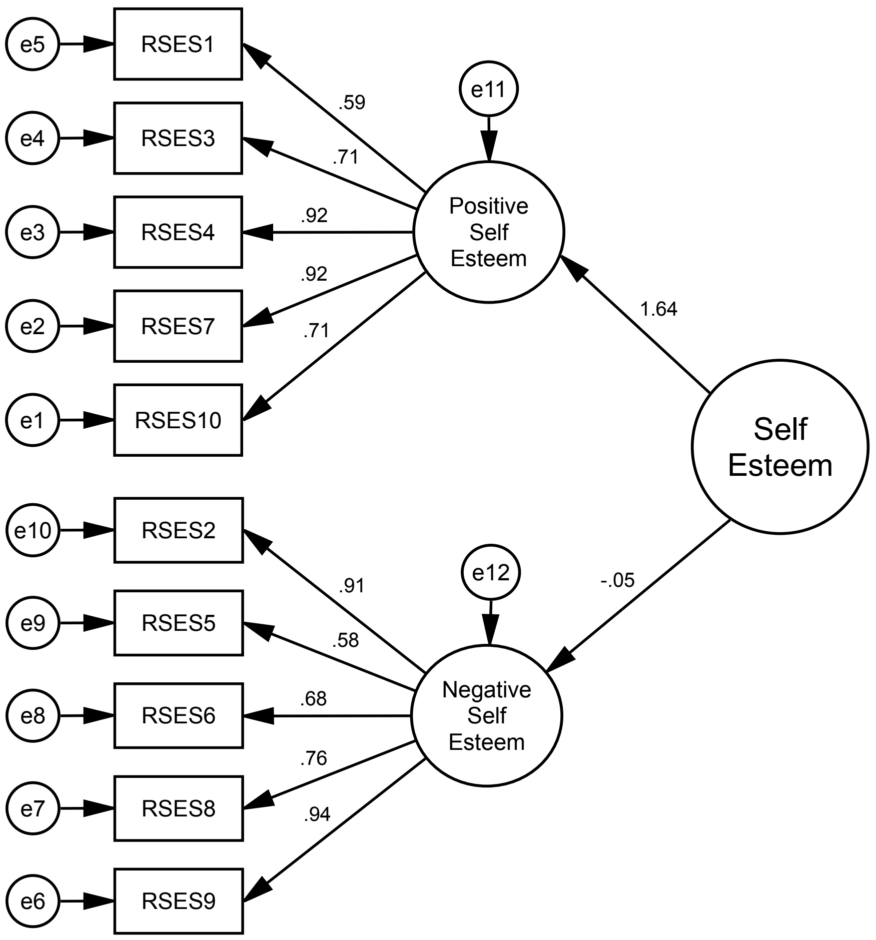

Figure 5. Hierarchical factor model of RSES-Indonesian version (Model 3)

Click here to access/download
Supporting Information
S1 Dataset.xlsx

~~Construct~~ Structural Validity of the Rosenberg Self-Esteem Scale in Patients with Schizophrenia in Indonesia

Muhammad Muslih [a, b], Min-Huey Chung [a, c*]

[a] School of Nursing, College of Nursing, Taipei Medical University, Taiwan

[b] School of Nursing, Faculty of Health Science, Universitas Muhammadiyah Malang, Indonesia

[c] Department of Nursing, Shuang Ho Hospital, Taipei Medical University, New Taipei City, Taiwan

Corresponding Author:

Min-Huey Chung, PhD, RN, FAAN

School of Nursing, College of Nursing, Taipei Medical University

No. 250, Wu-Xing Street, Taipei, Taiwan, 110, ROC.

Email address: minhuey300@tmu.edu.tw

Abstract

Background: The Rosenberg self-esteem scale (RSES) is a commonly employed instrument for measuring self-esteem in the general population and those with mental illness. However, confirmatory factor analyses (CFA) to determine the factor structure and model fitstructural validity of the RSES for schizophrenia patients in Indonesia are limited.

Objectives: We examined the structural validity of the RSES as a measurement for patients with schizophrenia in Indonesia through confirmatory factor analyses (CFA), as well as assessing internal consistency and reliability.the validity and reliability evidence of the RSES by measuring confirmatory factors among patients with schizophrenia in Indonesia.

Methods: The sample comprised 260 participants. Over two weeks, 30 subjects were added to investigate test-retest reliability. The structural validity evidenceanalyzed was based on a CFA to determine the model fit.

We used internal consistency (Cronbach's alpha) and test-retest reliability to evaluate the reliability evidence. The validity evidence was based on a CFA to determine the model fit.

Results: Four different models were analyzed in this study. Considering the single-factor model (Model 1a), the overall fit criteria were inadequate. However, after some modification indices, all fit criteria were significantly adequate (Model 1b). The adequacy of all fit standards remained satisfactory when the two-factor model (Model 2) and hierarchical model (Model 3) were applied.

The RSES had an alpha Cronbach coefficient of 0.75. While 0.89 and 0.88 for the positive and negative self-esteem subscale, respectively. Test-retest reliability yielded adequate results with an interclass correlation score ranging from 0.87 to 0.93. Four different models were analyzed in this study. Considering the single-factor model (Model 1a), the overall fit criteria were inadequate. However, after some modification indices, all fit criteria were significantly adequate (Model 1b). The adequacy

of all fit standards remained satisfactory when the two-factor model (Model 2) and hierarchical model (Model 3) were applied.

Conclusions: The current investigation provided evidence supporting the structural validity, internal consistency, and reliability of the RSES, indicating that the RSES can be considered a valid and reliable measurement. A two-factor model of RSES was an appropriate model to measure self-esteem in our study. This finding suggests that the use of the RSES is beneficial and applicable in assessing levels of self-esteem in individuals diagnosed with schizophrenia in Indonesia.

Keywords: self-esteem; validity; reliability, schizophrenia

1. Introduction

Self-esteem is an overall individual evaluation or appraisal of the self [1] and how a person thinks of themselves. Self-esteem is "the degree to which a person values, approves of, or likes himself or herself." [2]. Self-esteem is a crucial component of mental health and general psychological well-being. It influences an individual's achievements and successes, social interactions, and ability to cope with environmental stressors [3, 4]. Individuals with high self-esteem believe they possess many positive qualities and attitudes toward themselves [5]. In summary, self-esteem is a pivotal psychological construct [6] that controls several facets of an individual's existence, encompassing mental well-being, accomplishments, interpersonal engagements, and coping abilities [3, 4].

There is a reciprocal relationship between self-esteem and mental illnesses [7]. A previous study found that self-esteem plays a pivotalvital role in developing diverse mental illnesses and social problems encompassing a range of internalizing issues, such as depression, suicidal tendencies, eating disorders, and anxiety, as well as externalizing problems, including violence and substance abuse [8]. Conversely, it has been hypothesized that mental illnesses can lead to low self-esteem as a significant consequence [9]. Consistent with prior research, low self-esteem has been found to heighten

susceptibility to the onset of mental illness [10] low self-esteem has been found to heighten susceptibility to the onset of mental illness. In contrast, the existence of mental illness subsequently diminishes self-esteem. Eventually, individuals with mental illnesses are likely to have fluctuating self-esteem levels [11]. HenceTherefore, drawing from the aforementioned explanation, we can conclude that, self-esteem is considered a component of self-assessment, which influences mental health and vice versa.

The Rosenberg Self-Esteem Scale (RSES), which was developed by Rosenberg [12], is one of the most extensively used instruments for measuring self-esteem globally [13-17]. Researchers often use the RSES to measure self-esteem in the clinical population, such as eating disorders [18], anxiety, depression [7], attention and emotional disorder [19], schizophrenia and bipolar disorder [20]. Other studies have tested the RSES in specific people, such as ex-prisoners [21], drug users [22], and single mothers [23]. Thus, the RSES applies to participants from various samples or populations. The RSES has been translated and adapted into a number of differentvariety of languages, including German [24], Dutch [25], Estonian [26], French [27], Portuguese [28], Spanish [29], Japanese [17], and Thai [14]; thus, making it applicable to participants from diverse samples or populations. It has been adapted across 53 nations with distinct ethnic groups and cultures [30]. This finding indicates that the RSES is widely used to measure self-esteem. Supporting this idea, a prior study suggests that the popularity of the RSES can be attributed to its brevity and simplicity, as it comprises only ten questions that can be completed within a short timeframe of 1 to 2 minutes [13].

Multiple countries, including Indonesia, have implemented the RSES to measure the self-esteem of college students and the general population [30]. Some previous studies have shown that self-esteem has been associated with schizophrenia [31-33]. For example, there was a significant correlation between a decrease in the intensity of adverse symptoms and an enhancement in self-esteem, and conversely [34]. However, psychometric analyses, including structural validity and reliability, in patients diagnosed with schizophrenia were not explicitly addressed in previous studies [30, 35].

In a prior study, the factor structure of the RSES was examined using psychometric tests, and it focused on adolescents [36], as they were the original target population of this scale. It has also been tested in adults [37] and the general population [24]. Other studies have tested the RSES in specific people, such as ex-prisoners [21], drug users [22], and single mothers [23]. Accordingly, the RSES applies to participants from various samples or populations. Nevertheless, there is no available evidence supporting the utilization of RSES among individuals diagnosed with schizophrenia in Indonesia.

The RSES has been translated and adapted into a variety of languages, including German [24], Dutch [25], Estonian [26], French [27], Portuguese [28], [29]Japanese [17], and Thai [14]. It has been adapted across 53 nations with distinct ethnic groups and cultures [30]. This finding indicates that the RSES is widely used to measure self-esteem. Supporting this idea, [13] suggest that the popularity of the RSES can be attributed to its brevity and simplicity, as it comprises only ten questions that can be completed within a short timeframe of 1 to 2 minutes.

Multiple countries, including Indonesia, have implemented the RSES to measure the self-esteem of college students and the general population [30]. Interestingly, some previous studies have shown that self-esteem has been associated with schizophrenia [31-33]. For example, there was a significant correlation between a decrease in the intensity of adverse symptoms and an enhancement in self-esteem, and conversely, [34]. Unfortunately, the scale for people diagnosed with schizophrenia was not evaluated using psychometric testing in a prior study [30]. Additionally, they only focused on internal consistency and factor structure invariance.

Nevertheless, there is no available evidence supporting the utilization of RSES among individuals diagnosed with schizophrenia in Indonesia. Hence, evaluating the RSES in individuals diagnosed with schizophrenia is imperative to ascertain its psychometric analysis. validity and reliability. This study aimed to assess the construct structural validity of the RSES as a measurement for patients with

schizophrenia in Indonesia through confirmatory factor analyses (CFA), as well as assessing internal consistency and reliability.

2. Methods

2.1 Participants

This is an instrumental questionnaire validation study. Two psychiatric hospitals and one psychiatric rehabilitation center in East Java, Indonesia, were visited to obtain the required data. We distributed the questionnaire from August 2018 to February 2019. Participants were recruited using the convenience sampling technique. The following requirements had to be met for someone to be included: (a) they had been diagnosed with schizophrenia; (b) aged ≥ 20 years; (c) hospitalized in a psychiatric ward; and (d) able to speak, read, and write Indonesian. The Mini-Mental State Examination (MMSE) was utilized to screen out participants with cognitive impairment (i.e., MMSE scores < 24).

The size of 200 participants required in this study is acceptable based on recommendations from prior studies [38, 39]. In this study, data was missing from four questionnaires because they were not accurately completed, and 21 participants were excluded because their MMSE score was less than 24. Considering the response rate of 20%, the final sample comprised 260 participants. In addition, we recruited an additional 30 individuals to investigate test-retest reliability over two weeks.

2.2 Instruments

The study incorporates many socio-demographic data, including age as a continuous variable. The remaining variables as categorical variables, namely gender (1=male; 2=female), marital status (1=single; 2=married; 3=divorced, or widowed), employment status (1=employed; 2=unemployed), source of income (1=personal income; 2=family support; 3=personal and family support), education (1=elementary; 2=junior; 3=high school; 4=university/ college), previous hospitalization (1=yes; 2=no), and onset of illness (1=<1 year; 2=1-5 years; 3=>5 years).

The RSES is not licensed and is available for public use. Information about the scale can easily be gathered, and permission to use this resource can be sought at https://socy.umd.edu/about-us/rosenberg-self-esteem-scale. The scale consists of 10 items evaluated on a 4-point Likert scale from 1 (strongly disagree) to 4 (strongly agree). Scores vary from 10 to 40, with higher scores indicating a more positive self-esteem appraisal. To measure the reverse score, five questions are worded positively (items 1, 3, 4, 7, and 10), while five are worded negatively (items 2, 5, 6, 8, and 9).

The validity and reliability evidence of the RSES were assessed in prior studies, and the obtained results are presented herein. Concerning the evaluation of validity evidence (construct validity), construct validity, prior studies have demonstrated that the RSES yielded an excellent model fit [21, 40]. According to a previous study [41] it is suggested to ensure that the Cronbach alpha criteria for each sub-scale is ≥ 0.70. The Cronbach's alpha coefficients for the positive and negative self-esteem subscales were determined to be 0.96 and 0.98, respectively [21]. A prior study conducted on individuals who are native English speakers has also yielded Cronbach alpha coefficients of 0.87 and 0.75 for the subscales measuring positive and negative self-esteem, respectively [17]. Concerning the evaluation of validity evidence (construct validity), prior studies have demonstrated that the RSES yielded an excellent model fit [21, 40].

2.3 Translation Procedure

Due to their specificity and straightforwardness, we adhered to the parameters suggested by a previous study [42]. Initially, the original questionnaire was translated into Indonesian by two translators, a psychiatrist and a professional translator whose native language was Indonesian, with the author's approval. Both were bilingual and English-proficient. Second, we compared the two translated versions and created a new draft by combining the terminology and phrases supplied by the two translators in the previous step. Thirdly, the information was back-translated by two more independent translators with the same credentials and qualities as the first translators. Fourth, we compared the original questionnaire with the two back-translations of the questionnaire from the third phase.

Considering the distance and time variations, we communicated with all four translators by email at this stage. Fifth, 30 volunteers were selected from a psychiatric hospital to evaluate the clarity of the questionnaire's instructions, items, and response format. In addition, we asked two professionals (a psychiatrist and a psychologist) for revisions and ideas. In the final phase, the full Indonesian version of the RSES scale was administered to the study sample and tested the evidence of validity and reliability (See Figure 1)..

2.4 Statistical Analysis

This study utilized SPSS and AMOS version 23.0 software (IBM; Armonk, New York, USA). All statistical significance was indicated by a p-value < 0.05.

Descriptive analysis

Descriptive statistics were used to present the demographic characteristics of the study. The proportion of participants who obtained minimum and maximum scores is defined as floor and ceiling effect, respectively. Continuous variables are presented as means and standard deviations, whereas categorical variables are expressed as frequencies and percentages. The quantitative characteristics of the RSES were computed as mean, standard deviation (SD), skewness, and kurtosis. A skewness value between −1 and 1 was considered adequate [43]. The degree of vertical spread in the mean distribution corresponded to the kurtosis. The normality was assumed if the kurtosis value was less than 2.5 times the standard error [44]. said that normality was assumed if the kurtosis value was less than 2.5 times the standard error.

Reliability Structural validity.evidence

Structural validity pertains to the extent to which the scores of a Patient-Reported Outcome Measure (PROM) accurately represent the underlying structure of the construct being measured [41]. Assessment of structural validity is typically conducted through the use of CFA [41, 45]. A CFA was

carried out to assess how well the RSES model fits the data. The following fit indices were utilized during the evaluation process: X^2/df, the comparative fit index (CFI), incremental fit index (IFI), the Tucker–Lewis index (TLI), goodness-of-fit index (GFI), adjusted goodness-of-fit index (AGFI), the standardized root mean square residual (SRMR), and the root mean square error of approximation (RMSEA). A chi-square with a degree of freedom ratio of less than 5.0 indicated that the model was a good fit [46]. An acceptable model fit was characterized by a GFI greater than 0.80 [47] and an AGFI of 0.80 to 0.90 [48-52]. When the CFI, IFI, and TLI values were all greater than 0.90 [53], the SRMR value was less than 0.08 [53, 54], and the RMSEA value was less than 0.10 [47], the model fit was deemed to be satisfactory. Akaike Information Criterion (AIC) and Bayesian Information Criterion (BIC) were used to evaluate the alternative model, with lower AIC and BIC values indicating the best model fit [55, 56].

Field Code Changed

The ~~The composite reliability (CR) measurement and~~ average variance extracted (AVE) are an integral ~~components~~component of the comprehensive construct validity assessment. ~~The values of CR were more significant than 0.70 [57]~~, and an AVE score greater than 0.50 indicates an a~~was more critical than 0.50~~dequate result [58].

~~*Internal consistency (Cronbach's alpha) and the interclass correlation coefficient (ICC) were used to evaluate the RSES' test-retest reliability. A Cronbach's alpha equal to or better than 0.70 demonstrates adequate internal consistency*~~ *[59, 60]*~~*. The ICC score between 0.75 and 0.90 indicated satisfactory reliability and consistency between two-time measurements, and a score greater than 0.90 revealed excellent reliability*~~ *[61]*.

Internal consistency.~~*Validity evidence*~~

Internal consistency refers to "the extent of interrelatedness among the items and is commonly evaluated using Cronbach's alpha" [62]. A Cronbach's alpha equal to or better than 0.70 demonstrates an adequate internal consistency [59, 60]. The composite reliability (CR) was used to

measure a robust internal consistency. The values of CR greater than 0.70 indicated a significant result [57].

Reliability.

Interclass correlation coefficient (ICC) was used to evaluate the reliability [41]. The ICC score between 0.75 and 0.90 indicated satisfactory reliability and consistency between two-time measurements, and a score greater than 0.90 revealed excellent reliability [61].

A CFA was carried out to assess how well the RSES model fits the data. The following fit indices were utilized during the evaluation process: X^2/df, the comparative fit index (CFI), incremental fit index (IFI), the Tucker–Lewis index (TLI), goodness-of-fit index (GFI), adjusted goodness-of-fit index (AGFI), the standardized root mean square residual (SRMR), and the root mean square error of approximation (RMSEA). A chi-square with a degree of freedom ratio of less than 5.0 indicated that the model was a good fit [46]. An acceptable model fit was characterized by a GFI greater than 0.80 [47] and an AGFI of 0.80 to 0.90 [48-52]. When the CFI, IFI, and TLI values were all greater than 0.90 [53], the SRMR value was less than 0.08 [53, 54], and the RMSEA value was less than 0.10 [47], the model fit was deemed to be satisfactory. Akaike Information Criterion (AIC) and Bayesian Information Criterion (BIC) were used to evaluate the alternative model, with lower AIC and BIC values indicating the best model fit [55, 56].

The composite reliability (CR) measurement and average variance extracted (AVE) are integral components of the comprehensive construct validity assessment. The values of CR were more significant than 0.70 [57], and AVE was more critical than 0.50 [58].

2.5 Ethical Approval

The Ethics Research Committee approved this research of the University of Muhammadiyah Malang on July 19, 2018 (approval number: E.5.a/239/KEPK- UMM/VII/2018).

Field Code Changed

3. Results

Table 1 presents the clinical demographics of the study. The mean age of the 260 participants was 38.13 (SD = 9.56). Most participants were men (169, 65%) and single (139, 53.5%). Most were unemployed (173, 66.5%) and received financial support from their families (196, 75.4%). For 127 participants (48.8%), the highest educational level was senior high school. In total, 220 participants (84.6%) had previously been hospitalized, and the illness duration to onset was >5 years for 142 participants (54.6%).

Table 2 displays the descriptive statistics, inter-item correlation, and item-total correlation of the RSES. Item 8 and item 3 received the highest and lowest mean scores of 2.57 (SD = 0.87) and 1.92 (SD = 0.81), respectively. The skewness score ranged from 0.39 to 0.90 for the total RSES items, and the kurtosis score ranged from 0.74 to 0.75. The item-total correlation varied from 0.47 to 0.6. Floor and ceiling effect was found at 5.80% - 54.20%, respectively (Table 34).

3.1 Structural validity.

As presented in Table 3, the CR was calculated for positive and negative factors, and the values were 0.92 and 0.91, respectively. The AVE values were 0.69 and 0.68, and the square roots of the AVE were 0.83 and 0.82, indicating that each measured variable was significant. The goodness of indices for all alternative models is shown in Table 34. Considering the single-factor or uni-dimensional -model (Model M1aa), the overall fit criteria were inadequate (See Figure 21). However, after some modification indices (See Figure 32), all fit criteria were significantly adequate (ModelM 1b1b). The adequacy of all fit criteria remained satisfactory when the two-factor model (Model 22) and hierarchical model (Model 33) were applied (See Figures 43 and Figure 54). The AVE values were 0.69 and 0.68, and the square roots of the AVE were 0.83 and 0.82, indicating that each measured variable was significant (Table 4)..

3.13.2 Internal consistency and rReliability evidence.

The RSES had an alpha coefficient of 0.75, according to Cronbach's method. The results of Cronbach's alpha, which measures internal consistency, came in at 0.89 and 0.88 for each subscale (factor), indicating acceptable reliabilityinternal consistency. As presented in Table 4, the CR was calculated for positive and negative factors, and the values were 0.92 and 0.91, respectively. Test-retest reliability exhibited satisfactory results, with an ICC between 0.87 and 0.93 (Table 43).

3.2 Validity evidence

As presented in Table 3, the CR was calculated for positive and negative factors, and the values were 0.92 and 0.91, respectively. The AVE values were 0.69 and 0.68, and the square roots of the AVE were 0.83 and 0.82, indicating that each measured variable was significant. The goodness of indices for all alternative models is shown in Table 4. Considering the single-factor model (Model 1a), the overall fit criteria were inadequate (See Figure 1). However, after some modification indices (See Figure 2), all fit criteria were significantly adequate (Model 1b). The adequacy of all fit criteria remained satisfactory when the two-factor model (Model 2) and hierarchical model (Model 3) were applied (See Figures 3 and 4).

4. Discussion

This study aimed to establish the construct structural validity of the RSES as well as assess internal consistency and reliability. .We included patients with schizophrenia in our study, which was not the case in the previous study by [30]. The sample size of our studyOur sample size was adequate to perform factor analysis. In addition, evidence of the constructthe structural validity of the RSES was demonstrated through CFA, an approach that has not been conducted in prior studies in Indonesia.contrary to previous studies conducted in Indonesia.

Through the CFA, The total score on the Indonesian version of the RSES had a Cronbach's alpha coefficient of 0.75. In contrast, the positive self-esteem subscale had a coefficient of 0.89, and the

negative self-esteem subscale had a coefficient of 0.88. In line with this finding, a previous investigation found satisfactory levels of internal consistency, as measured by a Cronbach's alpha coefficient that ranged from 0.81 to 0.91 [13, 14, 25, 30]. Our results were consistent with a previous study by [63], conducted in individuals with severe mental illnesses, not specific only to patients with schizophrenia and reported strong internal consistency. This study also showed that the RSES has good internal consistency, especially for a particular group of people (those with schizophrenia).

In addition, we looked at the test-retest reliability of the RSES. The ICC values generated were 0.93 for the positive self-esteem subscale and 0.87 for the negative self-esteem subscale. The ICC results were adequate, indicating the stability of each factor of the RSES. Additionally, it was observed that there was a high correlation coefficient between the test-retest reliability and Cronbach's alpha. In conclusion, the findings of this study provide evidence supporting the robust reliability of the RSES as a reliable instrument for assessing self-esteem in individuals diagnosed with schizophrenia in the Indonesian context.

wWe examineexamine four models of structural validity in our study, which are;: the single-factor or uni-dimensional model (Model M1aa), the single-factor with correlated error (Model 1b1b), the two-factor model (Model 22), and the hierarchical model (Model 33). The results of Model 1a indicate that there is inadequate evidence to support the adequacy of a single factor. The findings from Model 1a, suggest that there is insufficient data to support the acceptance of a single factor. Where every model fit criterion failed to meet the required levels. A notable distinction was observed while implementing the correlated error in Model 1b1b, as it demonstratesdemonstrating an enhancement in the adequate good fit criteria. This adjustment was carried outdone on the the negative self-esteem factor, a negatively worded item. This finding aligns with some prior studies that have examined the presence of method effects related to negative items on the RSES [64-66]. While some of the model fit criteria are met, it is unfortunate that this model also fell outside of other criteria. The BIC value is significantly higher compared to models 2 and 3. Following a previous study, the difference in BIC

score greater than 10 provides strong evidence of the model [67]. If the objective is to achieve a goodness of fit, the BIC is the preferred option. Therefore, using BIC is more advantageous when selecting an accurate model [68]. ~~The model with a single factor with correlated error yielded the best model fit.~~

~~Surprisingly,~~ The adequate fit indexes were also obtained in Models 2~~2~~ and 3~~3~~. The two-factor and hierarchical models exhibit comparable model fit in their respective analyses. Based on the findings mentioned ~~earlier~~above, it is suggested that the ~~Rosenberg Self-Esteem Scale (RSES)~~RSES can be characterized as ~~conceptualized as comprising two distinct constructs~~two factors, ~~specifically~~ which are positive ~~self-esteem~~ and negative self-esteem. A previous study also referred to these two factors as positive and negative self-esteem [69]. The influence of wording effect on scale items may result in or contribute to a two-factor model. The wording item effect, then further related to the method effect has been observed in earlier studies [37, 70], which suggests the presence of a two-factor of RSES [70]. In summary, our finding indicates that the RSES scale has an acceptable model fit with two factors, Similar findings were also demonstrated in a previous study that a two-factor model was deemed to have an adequate model fit [21, 36, 71, 72]. Based on our finding, we can conclude that the RSES with is a two-factor model was a valid instrument for people with schizophrenia in Indonesia. Acknowledging the necessity of reassessing the utilization of the RSES and its theoretical foundations in administering the scale to target populations is essential.

Field Code Changed

~~Similar findings were also demonstrated in a previous study that a two-factor model was deemed to have an adequate model fit~~ [21, 36, 71, 72]~~. In summary, our evidence indicates that the RSES scale construct has an acceptable model fit, which means that the RSES is suitable for individuals with schizophrenia in Indonesia. In short, the evidence from our study shows that the RSES scale construct fits well, which means that the RSES is a valid scale to use with people with schizophrenia in Indonesia.~~

Field Code Changed

Based on the rationale mentioned earlier, our study's results indicate that the RSES is a unidimensional measure of overall self-esteem, as initially proposed by . However, the influence of wording impacts on scale items may result in or contribute to bi-dimensionality.As previously established by various studies, RSES was also shown to assess two separate variables (positive and negative self-esteem) . According to a prior study [73] it has been firmly indicated that the RSES is uni-dimensional. However, the influence of wording impacts on scale items may result in or contribute to bi-dimensionality. Acknowledging the necessity of reassessing the utilization of the RSES and its theoretical foundations in administering the scale to target populations is imperative.

The total score on the Indonesian version of the RSES had a Cronbach's alpha coefficient of 0.75. In contrast, the positive and negative self-esteem subscale had a coefficient of 0.89, and 0.88 respectively. In line with this finding, a previous investigation found satisfactory levels of internal consistency, as measured by a Cronbach's alpha coefficient that ranged from 0.81 to 0.91 [13, 14, 25, 30]. Our results waswere consistent with a previous study by Torrey, Mueser, McHugo, and Drake [63], which was conducted inconducted on individuals with severe mental illnesses, not specific only to patients with schizophrenia, and which reported strong internal consistency. To demonstrate more robust internal consistency, composite reliability also exhibits favorable outcomes.

In addition, we looked at the test-retest reliability of the RSES. The ICC values generated were 0.93 and 0.87 for the positive and negative self-esteem respectively. The ICC results were adequate, indicating the stability of each factor of the RSES. Additionally, it was observed that there was a high correlation coefficient between the test-retest reliability and Cronbach's alpha. In conclusion, the findings of this study provide evidence supporting the robust reliability of the RSES as a reliable instrument for assessing self-esteem in individuals diagnosed with schizophrenia in Indonesia.

The present study also has limitations. Because our study sample included only patients with schizophrenia, these findings cannot be extrapolated to populations with other mental illnesses. Moreover, a significant majoritymost of our study's participants are men, leading to a more prominent

interpretation of item scores among this group. Future studies should aim to expand the participant pool by including individuals diagnosed with a diverse range of mental illnesses while also ensuring a balanced representation of both genders. In addition to the limitations mentioned above, it is essential to note that our study produced favorable outcomes evidence regarding the structural validity, internal consistency, and reliability. of evidence. Hence establishing the RSES as a valid and reliable questionnaire appropriate for implementation within the tested sample group.

The model with a single factor with correlated error yielded the best model fit.

5. Conclusion

The current investigation provided evidence supporting the construct structural validity, internal consistency, and and reliability of the RSES, indicating that the RSES can be considered a valid and reliable measurement. A two-factor model of RSES was an appropriate model to measure self-esteem in our study. This finding suggests that the use of the RSES is beneficial and applicable in assessing levels of self-esteem in individuals diagnosed with schizophrenia in Indonesia. Nevertheless, further research is needed to understand better the characteristics of the method factors for different populations.

As previously stated, positive and negative item wording is still a major consideration in psychometric analysis.

Supporting information

S1 Dataset

Data availability

All relevant data are within the paper and its S1 Dataset files. Please inform the authors if data are being used.

Conflict of Interest

The authors declare that they have no conflicts of interest.

Funding

The authors did not receive any funding for this study.

Acknowledgment

We would like to acknowledge Wallace Academic Editing Company for editing this manuscript.

Authors' Contributions

M.M collected, analyzed, and interpreted the data and drafted the manuscript. M.H.C studied and analyzed the data and co-drafted the manuscript.

References

1. Cast AD, Burke PJ. A theory of self-esteem. Social forces. 2002;80(3):1041-68.

2. Blascovich J, Tomaka J. Measures of self-esteem. Measures of personality and social psychological attitudes. 1991;1:115-60.

3. Battle J. Promoting self-esteem, achievement and well being: An effective instructional curriculum for all levels: James Battle and Associates; 1994.

4. Huang C, Dong N. Factor structures of the Rosenberg self-esteem scale. European Journal of Psychological Assessment. 2012.

5. Bonfine N. Stigma self concept and stigma resistance among individuals with mental illness [Disertation]. Ann Arbor: Kent State University; 2013.

6. von Soest T, Wichstrøm L, Kvalem IL. The development of global and domain-specific self-esteem from age 13 to 31. Journal of Personality and Social Psychology. 2016;110(4):592.

7. van Tuijl LA, de Jong PJ, Sportel BE, de Hullu E, Nauta MH. Implicit and explicit self-esteem and their reciprocal relationship with symptoms of depression and social anxiety: A longitudinal study in adolescents. Journal of behavior therapy and experimental psychiatry. 2014;45(1):113-21.

8. Mann M, Hosman CM, Schaalma HP, de Vries NK. Self-esteem in a broad-spectrum approach for mental health promotion. Health Educ Res. 2004;19(4):357-72. Epub 2004/06/17. doi: 10.1093/her/cyg041. PubMed PMID: 15199011.

9. Link BG, Struening EL, Neese-Todd S, Asmussen S, Phelan JC. Stigma as a barrier to recovery: The consequences of stigma for the self-esteem of people with mental illnesses. Psychiatric services. 2001;52(12):1621-6.

10. Silverstone PH, Salsali M. Low self-esteem and psychiatric patients: Part I – The relationship between low self-esteem and psychiatric diagnosis. Annals of General Hospital Psychiatry. 2003;2(1):2. doi: 10.1186/1475-2832-2-2.

11. Romm KL, Rossberg JI, Hansen CF, Haug E, Andreassen OA, Melle I. Self-esteem is associated with premorbid adjustment and positive psychotic symptoms in early psychosis. BMC psychiatry. 2011;11(1):1-8.

12. Rosenberg M. Society and the Adolescent Self-Image. Revised edition. Middletown, CT: Wesleyan University Press; 1989.

13. Sinclair SJ, Blais MA, Gansler DA, Sandberg E, Bistis K, LoCicero A. Psychometric properties of the Rosenberg Self-Esteem Scale: Overall and across demographic groups living within the United States. Evaluation & the health professions. 2010;33(1):56-80.

14. Tinakon W, Nahathai W. A comparison of reliability and construct validity between the original and revised versions of the Rosenberg Self-Esteem Scale. Psychiatry investigation. 2012;9(1):54.

15. Martin CR, Thompson DR, Chan DS. An examination of the psychometric properties of the Rosenberg Self-Esteem Scale (RSES) in Chinese acute coronary syndrome (ACS) patients. Psychology, health & medicine. 2006;11(4):507-21.

16. Martín-Albo J, Núñez JL, Navarro JG, Grijalvo F. The Rosenberg Self-Esteem Scale: translation and validation in university students. The Spanish journal of psychology. 2007;10(2):458-67.

17. Mimura C, Griffiths P. A Japanese version of the Rosenberg Self-Esteem Scale: Translation and equivalence assessment. Journal of Psychosomatic Research. 2007;62(5):589-94.

18. Salerno L, Ingoglia S, Coco GL. Competing factor structures of the Rosenberg Self-Esteem Scale (RSES) and its measurement invariance across clinical and non-clinical samples. Personality and Individual Differences. 2017;113:13-9.

19. Henriksen IO, Ranøyen I, Indredavik MS, Stenseng F. The role of self-esteem in the development of psychiatric problems: a three-year prospective study in a clinical sample of adolescents. Child and adolescent psychiatry and mental health. 2017;11:1-9.

20. Oliveira SE, Esteves F, Carvalho H. Clinical profiles of stigma experiences, self-esteem and social relationships among people with schizophrenia, depressive, and bipolar disorders. Psychiatry research. 2015;229(1-2):167-73.

21. Boduszek D, Hyland P, Dhingra K, Mallett J. The factor structure and composite reliability of the Rosenberg Self-Esteem Scale among ex-prisoners. Personality and individual differences. 2013;55(8):877-81.

22. Wang J, Siegal HA, Falck RS, Carlson RG. Factorial structure of Rosenberg's Self-Esteem Scale among crack-cocaine drug users. Structural Equation Modeling. 2001;8(2):275-86.

23. Hatcher J, Hall LA. Psychometric properties of the Rosenberg self-esteem scale in African American single mothers. Issues in Mental Health Nursing. 2009;30(2):70-7.

24. Roth M, Decker O, Herzberg PY, Brähler E. Dimensionality and norms of the Rosenberg Self-Esteem Scale in a German general population sample. European Journal of Psychological Assessment. 2008;24(3):190-7.

25. Franck E, De Raedt R, Barbez C, Rosseel Y. Psychometric properties of the Dutch Rosenberg self-esteem scale. Psychologica Belgica. 2008;48(1):25-35.

26. Pullmann H, Allik J. The Rosenberg Self-Esteem Scale: its dimensionality, stability and personality correlates in Estonian. Personality and Individual differences. 2000;28(4):701-15.

27. Vallieres EF, Vallerand RJ. Traduction et validation canadienne- française de l'échelle de l'estime de soi de Rosenberg. International journal of psychology. 1990;25(2):305-16.

28. Vasconcelos-Raposo J, Fernandes HM, Teixeira CM, Bertelli R. Factorial validity and invariance of the Rosenberg Self-Esteem Scale among Portuguese youngsters. Social Indicators Research. 2012;105(3):483-98.

29. Mayordomo T, Gutierrez M, Sales A. Adapting and validating the Rosenberg Self-Esteem Scale for elderly Spanish population. International Psychogeriatrics. 2020;32(2):183-90.

30. Schmitt DP, Allik J. Simultaneous administration of the Rosenberg Self-Esteem Scale in 53 nations: exploring the universal and culture-specific features of global self-esteem. Journal of personality and social psychology. 2005;89(4):623.

31. Moritz S, Veckenstedt R, Randjbar S, Vitzthum F, Karow A, Lincoln TM. Course and determinants of self- esteem in people diagnosed with schizophrenia during psychiatric treatment. Psychosis. 2010;2(2):144-53.

32. Haug E, Øie MG, Andreassen OA, Bratlien U, Romm KL, Møller P, et al. The association between anomalous self-experiences, self-esteem and depressive symptoms in first episode schizophrenia. Frontiers in human neuroscience. 2016;10:557.

33. Kim EY, Jang MH. The Mediating Effects of Self-Esteem and Resilience on the Relationship Between Internalized Stigma and Quality of Life in People with Schizophrenia. Asian Nursing Research. 2019;13(4):257-63. doi: https://doi.org/10.1016/j.anr.2019.09.004.

34. Jones RM, Hansen L, Moskvina V, Kingdon D, Turkington D. The relationship between self- esteem and psychotic symptoms in schizophrenia: A longitudinal study. Psychosis. 2010;2(3):218-26.

35. Pardede JA, Keliat BA, Wardani IY. The Symptoms of Low Self-Esteem Decline after Being Given Acceptance and Commitment Therapy. Adv Practice Nurs. 2020;5(170):10.37421.

36. Marsh HW, Scalas LF, Nagengast B. Longitudinal tests of competing factor structures for the Rosenberg Self-Esteem Scale: traits, ephemeral artifacts, and stable response styles. Psychological assessment. 2010;22(2):366.

37. Gana K, Saada Y, Bailly N, Joulain M, Hervé C, Alaphilippe D. Longitudinal factorial invariance of the Rosenberg Self-Esteem Scale: Determining the nature of method effects due to item wording. Journal of Research in Personality. 2013;47(4):406-16.

38. Ferrando Piera PJ, Lorenzo Seva U, Hernández Dorado A, Muñiz Fernández J. Decálogo para el Análisis Factorial de los Ítems de un Test. Psicothema. 2022.

39. Lloret-Segura S, Ferreres-Traver A, Hernandez-Baeza A, Tomas-Marco I. Exploratory item factor analysis: A practical guide revised and updated. Anales de Psicología. 2014;30(3):1151-69.

40. Xu ML, Leung SO. Effects of varying numbers of Likert scale points on factor structure of the Rosenberg Self- Esteem Scale. Asian Journal of Social Psychology. 2018;21(3):119-28.

41. Prinsen CA, Mokkink LB, Bouter LM, Alonso J, Patrick DL, De Vet HC, et al. COSMIN guideline for systematic reviews of patient-reported outcome measures. Quality of life research. 2018;27:1147-57.

42. Sousa VD, Rojjanasrirat W. Translation, adaptation and validation of instruments or scales for use in cross-cultural health care research: a clear and user-friendly guideline. Journal of Evaluation Clinical Practice. 2011;17:268-74. Epub 2010/09/30. doi: 10.1111/j.1365-2753.2010.01434.x. PubMed PMID: 20874835.

43. Indrayan A, Malhotra RK. Medical biostatistics: Chapman and Hall/CRC; 2017.

44. Morgan GA, Griego OV. Easy use and interpretation of SPSS for Windows: Answering research questions with statistics: Psychology Press; 1998.

45. Polit DF. Assessing measurement in health: Beyond reliability and validity. International journal of nursing studies. 2015;52(11):1746-53.

46. Marsh HW, Hocevar D. Application of confirmatory factor analysis to the study of self-concept: First-and higher order factor models and their invariance across groups. Psychological bulletin. 1985;97(3):562.

47. Meyers LS, Gamst G, Guarino AJ. Applied Multivariate Research: Design and Interpretation: SAGE Publications; 2006.

48. Bentler PM. Comparative fit indexes in structural models. Psychological bulletin. 1990;107(2):238.

49. Marsh HW, Balla JR, McDonald RP. Goodness-of-fit indexes in confirmatory factor analysis: The effect of sample size. Psychological bulletin. 1988;103(3):391.

50. Cole DA. Utility of confirmatory factor analysis in test validation research. Journal of consulting and clinical psychology. 1987;55(4):584.

51. Floyd FJ, Widaman KF. Factor analysis in the development and refinement of clinical assessment instruments. Psychological assessment. 1995;7(3):286.

52. Greenspoon PJ, Saklofske DH. Confirmatory factor analysis of the multidimensional students' life satisfaction scale. Personality and Individual Differences. 1998;25(5):965-71.

53. Hu Lt, Bentler PM. Cutoff criteria for fit indexes in covariance structure analysis: Conventional criteria versus new alternatives. Structural equation modeling: a multidisciplinary journal. 1999;6(1):1-55.

54. Hoyle RH. Structural equation modeling: Concepts, issues, and applications: Sage; 1995.

55. Akaike H. A new look at the statistical model identification. IEEE transactions on automatic control. 1974;19(6):716-23.

56. Schwarz G. Estimating the dimension of a model. The annals of statistics. 1978:461-4.

57. Grewal R, Cote JA, Baumgartner H. Multicollinearity and measurement error in structural equation models: Implications for theory testing. Marketing science. 2004;23(4):519-29.

58. Hair J, Anderson R, Black B, Babin B. Multivariate Data Analysis: Pearson Education; 2016.

59. Polit DF, Beck CT. Essentials of nursing research: Appraising evidence for nursing practice: Lippincott Williams & Wilkins; 2010.

60. Tavakol M, Dennick R. Making sense of Cronbach's alpha. International journal of medical education. 2011;2:53.

61. Koo TK, Li MY. A guideline of selecting and reporting intraclass correlation coefficients for reliability research. Journal of chiropractic medicine. 2016;15(2):155-63.

62. Mokkink LB, Terwee CB, Patrick DL, Alonso J, Stratford PW, Knol DL, et al. The COSMIN study reached international consensus on taxonomy, terminology, and definitions of measurement

properties for health-related patient-reported outcomes. Journal of clinical epidemiology. 2010;63(7):737-45.

63. Torrey WC, Mueser KT, McHugo GH, Drake RE. Self-esteem as an outcome measure in studies of vocational rehabilitation for adults with severe mental illness. Psychiatric Services. 2000;51(2):229-33.

64. Mullen SP, Gothe NP, McAuley E. Evaluation of the factor structure of the Rosenberg Self-Esteem Scale in older adults. Personality and individual differences. 2013;54(2):153-7.

65. Aluja A, Rolland J-P, García LF, Rossier J. Dimensionality of the Rosenberg Self-Esteem Scale and its relationships with the three-and the five-factor personality models. Journal of Personality Assessment. 2007;88(2):246-9.

66. Makhubela M, Mashegoane S. Psychological validation of the Rosenberg Self-Esteem Scale (RSES) in South Africa: Method effects and dimensionality in black African and white university students. Journal of Psychology in Africa. 2017;27(3):277-81.

67. Bauldry S. Structural Equation Modeling. In: Wright JD, editor. International Encyclopedia of the Social & Behavioral Sciences (Second Edition). Oxford: Elsevier; 2015. p. 615-20.

68. Chakrabarti A, Ghosh JK. AIC, BIC and recent advances in model selection. Philosophy of statistics. 2011:583-605.

69. Ang RP, Neubronner M, Oh S-A, Leong V. Dimensionality of Rosenberg's self-esteem scale among normal-technical stream students in Singapore. Current Psychology. 2006;25(2):120-31.

70. Wu C-H. An examination of the wording effect in the Rosenberg Self-Esteem Scale among culturally Chinese people. Journal of Social Psychology. 2008;148(5).

71. Quilty LC, Oakman JM, Risko E. Correlates of the Rosenberg self-esteem scale method effects. Structural Equation Modeling. 2006;13(1):99-117.

72. Boduszek D, Shevlin M, Mallett J, Hyland P, O'Kane D. Dimensionality and construct validity of the Rosenberg self- esteem scale within a sample of recidivistic prisoners. Journal of Criminal Psychology. 2012;2(1):19-25.

73. Reise SP, Kim DS, Mansolf M, Widaman KF. Is the bifactor model a better model or is it just better at modeling implausible responses? Application of iteratively reweighted least squares to the Rosenberg Self-Esteem Scale. Multivariate behavioral research. 2016;51(6):818-38.

Table 1. Demographic Characteristics of the Study (*n* = 260)

**Characteristics**	**Participants (*n* = 260)**	
	**Mean (SD)**	**n (%)**
Age	38.13 (9.56)	
Gender		
Male		169 (65.00)
Female		91 (35.50)
Marital status		
Single		139 (53.46)
Married		81 (31.15)
Divorce or widowed		40 (15.39)
Employment status		
Employed		87 (33.50)
Unemployed		173 (66.50)
Source of income		
Personal income		30 (11.50)
Family support		196 (75.40)
Personal and family		34 (13.10)
Education		
Elementary school		63 (24.23)
Junior high school		44 (16.92)
Senior high school		127 (48.85)
University/college		26 (10.00)
Previous hospitalization		
Yes		220 (84.60)
No		40 (15.40)
Onset of illness		
<1 year		75 (28.85)
1–5 years		43 (16.54)
>5 years		142 (54.61)

*missing data = 4; **MMSE score < 24 = 21
SD = standard deviation; MMSE = Mini-Mental State Examination.

Table 2. Descriptive Statistics, Interitem, and Item-Total Correlation of the RSES Items (*n* = 260)

Items		Inter-item correlation (*n* = 260)									
		1	2	3	4	5	6	7	8	9	10
RSES 1	On the whole, I am satisfied with myself.	1									
RSES 2	At times, I think I am no good at all.	−0.11	1								
RSES 3	I feel that I have a number of good qualities.	0.56	−0.12	1							
RSES 4	I am able to do things as well as most other people.	0.51	−0.04	0.63	1						
RSES 5	I feel I do not have much to be proud of.	−0.03	0.48	−0.13	−0.11	1					
RSES 6	I certainly feel useless at times.	−0.00	0.62	−0.04	−0.01	0.48	1				
RSES 7	I feel that I'm a person of worth, at least on an equal plane with others.	0.52	−0.01	0.64	0.86	−0.07	0.06	1			
RSES 8	I wish I could have more respect for myself.	−0.19	0.71	−0.22	−0.15	0.43	0.50	−0.15	1		
RSES 9	All in all, I am inclined to feel that I am a failure.	−0.1	0.85	−0.13	−0.09	0.56	0.63	−0.05	0.71	1	
RSES 10	I take a positive attitude toward myself.	0.55	0.01	0.58	0.63	−0.01	−0.03	0.64	−0.08	−0.04	1
Item-total correlation		**0.49**	**0.61**	**0.50**	**0.58**	**0.47**	**0.59**	**0.62**	**0.46**	**0.61**	**0.59**
Mean		2.05	2.39	1.92	1.98	2.32	2.30	2.02	2.57	2.31	1.97
SD		0.94	0.86	0.81	0.84	0.86	0.91	0.83	0.87	0.83	0.86
Skewness		0.58	−0.02	0.90	0.69	0.32	0.21	0.59	−0.39	0.28	0.72
Kurtosis		−0.55	−0.68	0.75	0.05	−0.47	−0.74	−0.08	−0.53	−0.40	0.00

SD = standard deviation

Table 3. Goodness of fit indexes of the RSES Indonesian version.

Item	Model 1	Model 2	Model 3	Model 4
X^2/df	24.50	2.96	2.93	2.93
GFI	0.55	0.93	0.93	0.93
AGFI	0.30	0.88	0.88	0.88
CFI	0.48	0.97	0.96	0.96
TLI	0.33	0.94	0.95	0.95
IFI	0.48	0.97	0.96	0.96
SRMR	0.19	0.03	0.04	0.04
RMSEA	0.30	0.08	0.08	0.08
AIC	897.634	133.992	141.497	141.497
BIC	968.848	240.812	216.27	216.27

df = degree of freedom GFI = goodness of fit index; AGFI = adjusted goodness of fit index; CFI = comparative fit index; TLI = Tucker-Lewis index; IFI = incremental fit index; SRMR = standardized root mean square residual; RMSEA = root mean square error of approximation; AIC = Akaike information criterion; BIC = Bayesian information criterion

Table 4~~3~~. Reliability Analysis for the Indonesian Adaptation of the Rosenberg Self-Esteem Scale (*n* = 260)

Items	% floor effect	% ceiling effect	Cronbach's alpha	Test-retest reliability		AVE	CR
				ICC	95% CI		
Positive self-esteem			0.89	0.93	0.88–0.96	0.69	0.92
RSES 1	9.20	39.60					
RSES 3	6.50	54.20					
RSES 4	6.50	48.80					
RSES 7	5.80	48.80					
RSES 10	6.90	46.90					
Negative self-esteem			0.88	0.87	0.78–0.93	0.68	0.91
RSES 2	8.50	38.10					
RSES 5	10.40	47.30					
RSES 6	10.40	40.00					
RSES 8	10.80	50.00					
RSES 9	8.50	47.70					

CI = confidence interval, ICC = intraclass correlation coefficient, AVE = average variance extracted, CR = composite reliability,

Table 4. The model fits criteria of the RSES Indonesian version.

Item	Model 1a	Model 1b	Model 2	Model 3
X^2/df	24.50	2.96	2.93	2.93
GFI	0.55	0.93	0.93	0.93
AGFI	0.30	0.88	0.88	0.88
CFI	0.48	0.97	0.96	0.96
TLI	0.33	0.94	0.95	0.95
IFI	0.48	0.97	0.96	0.96
SRMR	0.19	0.03	0.04	0.04
RMSEA	0.30	0.08	0.08	0.08
AIC	897.634	133.992	141.497	141.497
BIC	968.848	240.812	216.27	216.27

df = degree of freedom GFI = goodness of fit index; AGFI = adjusted goodness of fit index; CFI = comparative fit index; TLI = Tucker-Lewis index; IFI = incremental fit index; SRMR = standardized root mean square residual; RMSEA = root mean square error of approximation; AIC = Akaike information criterion; BIC = Bayesian information criterion

Dear Editor and Reviewer

Manuscript ID: PONE-D-23-08354R1

Manuscript Title: "**Structural Validity of the Rosenberg Self-Esteem Scale in Patients with Schizophrenia in Indonesia**"

We sincerely thank the editor and all reviewers for their valuable suggestions and for allowing us to revise our manuscript entitled "Construct Validity of the Rosenberg Self-Esteem Scale in Patients with Schizophrenia in Indonesia". We have incorporated all the suggested changes into the manuscript and have highlighted the revised sections. At this moment, our responses and revisions are based on the editor and reviewer's comments.

EDITORIAL COMMENTS:

Please note that the discussion, as it is now, is confusing. On the one hand they state that the scale is unidimensional, but then conclude that the best structure is the two-factor model. Furthermore, in order to reach a conclusion on this, other parameters, such as factor loadings, should be taken into consideration. Factor loadings below 0.30 should not be included in these items because they do not explain enough variance.

Response: Thank you for your correction. The necessary amendments and recommendations provided by the reviewers have been incorporated into our work. The discussion section of our study has been revised.

(Page 10)

The adequate fit indexes were also obtained in Models 2 and 3. The two-factor and hierarchical models exhibit comparable model fit in their respective analyses. Based on the findings mentioned above, it is suggested that the RSES can be characterized as two factors, which are positive and negative self-esteem. A previous study also referred to these two factors as positive and negative self-esteem [69]. The influence of wording effect on scale items may result in or contribute to a two-factor model. The wording item effect, then further related to the method effect has been observed in earlier studies [37, 70], which suggests the presence of a two-factor of RSES [70]. In summary, our finding indicates that the RSES scale has an acceptable model fit with two factors, Similar findings were also demonstrated in a previous study that a two-factor model was deemed to have an adequate model fit [21, 36, 71, 72]. Based

on our finding, we can conclude that the RSES with is a two-factor model was a valid instrument for people with schizophrenia in Indonesia. Acknowledging the necessity of reassessing the utilization of the RSES and its theoretical foundations in administering the scale to target populations is essential.

(Page 12; conclusion).

The current investigation provided evidence supporting the structural validity, internal consistency, and reliability of the RSES, indicating that the RSES can be considered a valid and reliable measurement. A two-factor model of RSES was an appropriate model to measure self-esteem in our study. This finding suggests that the use of the RSES is beneficial and applicable in assessing levels of self-esteem in individuals diagnosed with schizophrenia in Indonesia.

With respect to loading factors, the initial manuscript we submitted details that our study employed loading factors ranging from 0.69 to 0.92, indicating that all loading factors exceeded 0.30. As previously mentioned in the last revision, our analysis was limited to CFA without EFA. We therefore do not report any factor loading in our recent revision.

REVIEWER #1 COMMENTS: No comments.

REVIEWER #2 COMMENTS:

I appreciate the authors' time spent in enhancing the manuscript. Statistically, it seems they have done an acceptable job. Nevertheless, there are still certain concerns I have regarding the manuscript's writing (Attached is a PDF where I've highlighted in red the elements that strike me as discordant in the text):

INTRODUCTION:

1. In summary, self-esteem is a pivotal psychological construct that controls several facets of an individual's existence, encompassing mental well-being, accomplishments, interpersonal engagements, and coping abilities-->Evidence is needed for this statement.

   Response: Thank you for your correction. We have corrected and replaced it following your suggestions.

   (Page 3; paragraph 1)

   In summary, self-esteem is a pivotal psychological construct [6] that controls several facets of an individual's existence, encompassing mental well-being, accomplishments, interpersonal engagements, and coping abilities [3, 4].

2. There exists a reciprocal relationship between self-esteem and mental illnesses. A previous study found that self-esteem plays a pivotal role in developing diverse mental illnesses and social problems encompassing a range of internalizing issues, such as depression, suicidal tendencies, eating disorders, and anxiety, as well as externalizing problems, including violence and substance abuse -->Evidence is needed for this statement. A visual representation or diagram displaying the relationship between constructs/variables would be interesting for the reader.

   Response: Thank you for your correction and suggestion. We have corrected and replaced it following your suggestions.

   (Page 3; paragraph 2)

   There is a reciprocal relationship between self-esteem and mental illnesses [7].

3. Researchers often use the RSES to measure self-esteem in the clinical population [15]--> Please add the characteristics of the population, diagnosis...

   Response: Thank you for your correction and suggestion. We have corrected and replaced it following your suggestions.

   (Page 3; paragraph 3)

   Researchers often use the RSES to measure self-esteem in the clinical population, such as eating disorders [18], anxiety, depression [7], attention and emotional disorder [19], schizophrenia and bipolar disorder [20]. Other studies have tested the RSES in specific people, such as ex-prisoners [21], drug users [22], and single mothers [23].

4. General Overview: The repetition of some ideas and the lack of a smooth transition between paragraphs make it difficult to read. Ensure that the citations are correctly referenced and that there is coherence in the bibliography throughout the text. Some key statements lack specific citations or references, which compromises the credibility of the text. Through a brief search, I find a large number of validations, such as those in Spanish, which the authors have not included in the relevant section of the introduction. An exposition of the structural differences and psychometric properties of the tool in the target population of the study should be made, in this case, in patients with schizophrenia.

   Response: Thank you for your correction and suggestion. We have corrected and replaced it following your suggestions, including the substitution of multiple sentences in the introduction to make it easier for readers to fully understand our idea. (See page 3-4; paragraph 1-5)

   (Page 3-4; paragraph 3)

   The RSES has been translated and adapted into a number of different languages, including German [24], Dutch [25], Estonian [26], French [27], Portuguese [28], Spanish [29], Japanese [17], and Thai [14]; thus, making it applicable to participants from diverse samples or populations.

METHODS:

5. Instruments-->Remove the numbering from the categorical variables. Is the instruments section appropriate to discuss the known evidence of internal consistency?

Response: Thank you for your correction and suggestion. We have corrected and replaced it following your suggestions. This section aims to elucidate the rationale for the utilization of this questionnaire. It will accomplish this by providing information about the questionnaire's developer, the scale employed, its interpretation, and the questionnaire's validity and reliability evidence, as established by prior research.

(Page 5)

The remaining variables as categorical variables, namely gender (male; female), marital status (single; married; divorced, or widowed), employment status (employed; unemployed), source of income (personal income; family support; personal and family support), education (elementary; junior; high school; university/ college), previous hospitalization (yes; no), and onset of illness (<1 year; 1-5 years; >5 years).

6. Translation procedure-->Include an explanatory diagram/figure of the process.

Response: Thank you for your correction and suggestion. We have provided the flow diagram of the translation procedure (see: Figure 1)

(Figure 1)

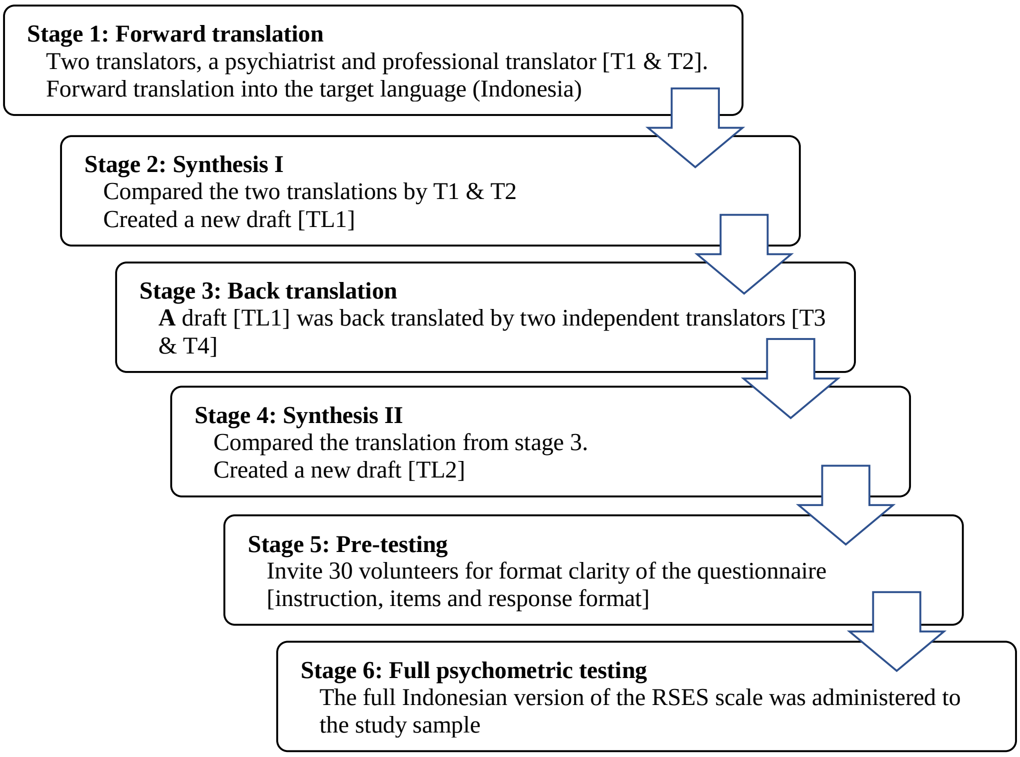

7. Statistical Analysis--> Please remove the sub-sections and write in a simpler manner.

   Response: Thank you for your correction and suggestion. We have corrected and replaced it following your suggestions. (Page 7-8).

8. Validity evidence--> What estimator have you used for the CFA? ULS, WLSMV?

   Response: Thank you for your correction and suggestion. Unweighted Least Squares (ULS) was used in our analysis, since our data meet the assumption as a continuous and normally distributed.

9. General Overview: Please present structural validity section before reliability and internal consistency. I refer you to COSMIN. The data on internal consistency cannot be interpreted before data on structural validity.

   Response: Thank you for your correction and suggestion. We have corrected and replaced it following your suggestions, as mentioned based on the COSMIN.

RESULTS:

10. General Overview: Please provide the evidence for structural validity before internal consistency and reliability. According to COSMIN (reference provided in the first review), internal consistency and reliability cannot be interpreted without first having evidence of structural validity.

    Response: Thank you for your correction and suggestion. We have corrected and replaced it following your suggestions.

    (Page 9)

    Structural validity.

    The goodness of indices for all alternative models is shown in Table 3. Considering the single-factor or uni-dimensional model (M1a), the overall fit criteria were inadequate (See Figure 2). However, after some modification indices (See Figure 3), all fit criteria were significantly adequate (M1b). The adequacy of all fit criteria remained satisfactory when the two-factor model (M2) and hierarchical model (M3) were applied (See Figures 4 and 5). The AVE values were 0.69 and 0.68, and the square roots of the AVE were 0.83 and 0.82, indicating that each measured variable was significant (Table 4).

    Internal consistency and reliability evidence.

The RSES had an alpha coefficient of 0.75, according to Cronbach's method. The results of Cronbach's alpha, which measures internal consistency, came in at 0.89 and 0.88 for each subscale (factor), indicating acceptable internal consistency. As presented in Table 4, the CR was calculated for positive and negative factors, and the values were 0.92 and 0.91, respectively. Test-retest reliability exhibited satisfactory results, with an ICC between 0.87 and 0.93 (Table 4).

DISCUSSION:

11. General Overview: The discussion still requires further development. Currently, the flow of information is disorganized. I also encourage the authors to delve into the results they have obtained and provide an explanation and reasoning behind their findings. They do not discuss the advantages of obtaining a single factor or the disadvantages of not obtaining it... I'm also unsure whether the studies they are comparing with have clinical or non-clinical samples. There is an anthropomorphic language that should be removed. The writing needs to be revised as it still does not adhere to AERA, APA, NMCE, COSMIN standards.

    Response: Thank you for your correction and suggestion. We have corrected and replaced it following your suggestions. Such as; *construct validity* was replaced specifically as *structural validity*; and *reliability* was replaced and divided as *internal consistency and reliability*. The details are on pages 9-11.

    Next, I copy fragments of the discussion that bother me when reading:

    "Surprisingly", "can be conceptualized as comprising two distinct constructs", "In short, the evidence from our study shows that the RSES scale construct fits well", Our sample size was adequate to perform factor analysis", "Our results were consistent with a previous study by [54], conducted in", "individuals with severe mental illnesses, not specific only to patients with schizophrenia and reported strong internal consistency".......

    Response: Thank you for your correction and suggestion. We have also improved the discussion section to make it easier for readers to understand what we mean in our study. The details are in the discussion section (pages 9-11). Furthermore, this manuscript was edited by Wallace Academic Editing Company to help us in providing writing corrections to our manuscript so that it complies with journal standards.

REVIEWER #3 COMMENTS:

1. Authors have successfully adressed all of my concerns. The manuscript can be considered for its publication now.

   Response: We express our gratitude for your kind attention**.**
