# Supplementary material for: Structural validity of the Rosenberg self-esteem scale in patients with schizophrenia in Indonesia
Source: PLoS One. 2024 May 10;19(5):e0300184. doi: 10.1371/journal.pone.0300184 (PMC11086833; doi:10.1371/journal.pone.0300184)
Supplement: Attachment — Submitted filename: Response to Reviewers (2)_20231229.docx [file pone.0300184.s003.docx]

Dear Editor and Reviewer

Manuscript ID: PONE-D-23-08354R1

Manuscript Title: “**Structural Validity of the Rosenberg Self-Esteem Scale in Patients with Schizophrenia in Indonesia**”

We sincerely thank the editor and all reviewers for their valuable suggestions and for allowing us to revise our manuscript entitled “Construct Validity of the Rosenberg Self-Esteem Scale in Patients with Schizophrenia in Indonesia”. We have incorporated all the suggested changes into the manuscript and have highlighted the revised sections. At this moment, our responses and revisions are based on the editor and reviewer’s comments.

--------------------------------------------------------------------------------------------------

**EDITORIAL COMMENTS:**

Please note that the discussion, as it is now, is confusing. On the one hand they state that the scale is unidimensional, but then conclude that the best structure is the two-factor model. Furthermore, in order to reach a conclusion on this, other parameters, such as factor loadings, should be taken into consideration. Factor loadings below 0.30 should not be included in these items because they do not explain enough variance.

**Response:** Thank you for your correction. The necessary amendments and recommendations provided by the reviewers have been incorporated into our work. The discussion section of our study has been revised.

(Page 10)

The adequate fit indexes were also obtained in Models 2 and 3. The two-factor and hierarchical models exhibit comparable model fit in their respective analyses. Based on the findings mentioned above, it is suggested that the RSES can be characterized as two factors, which are positive and negative self-esteem. A previous study also referred to these two factors as positive and negative self-esteem [69]. The influence of wording effect on scale items may result in or contribute to a two-factor model. The wording item effect, then further related to the method effect has been observed in earlier studies [37, 70], which suggests the presence of a two-factor of RSES [70]. In summary, our finding indicates that the RSES scale has an acceptable model fit with two factors, Similar findings were also demonstrated in a previous study that a two-factor model was deemed to have an adequate model fit [21, 36, 71, 72]. Based on our finding, we can conclude that the RSES with is a two-factor model was a valid instrument for people with schizophrenia in Indonesia. Acknowledging the necessity of reassessing the utilization of the RSES and its theoretical foundations in administering the scale to target populations is essential.

(Page 12; conclusion).

The current investigation provided evidence supporting the structural validity, internal consistency, and reliability of the RSES, indicating that the RSES can be considered a valid and reliable measurement. A two-factor model of RSES was an appropriate model to measure self-esteem in our study. This finding suggests that the use of the RSES is beneficial and applicable in assessing levels of self-esteem in individuals diagnosed with schizophrenia in Indonesia.

With respect to loading factors, the initial manuscript we submitted details that our study employed loading factors ranging from 0.69 to 0.92, indicating that all loading factors exceeded 0.30. As previously mentioned in the last revision, our analysis was limited to CFA without EFA. We therefore do not report any factor loading in our recent revision.

**REVIEWER #1 COMMENTS: No comments.**

**REVIEWER #2 COMMENTS:**

I appreciate the authors' time spent in enhancing the manuscript. Statistically, it seems they have done an acceptable job. Nevertheless, there are still certain concerns I have regarding the manuscript's writing (Attached is a PDF where I've highlighted in red the elements that strike me as discordant in the text):

INTRODUCTION:

1. In summary, self-esteem is a pivotal psychological construct that controls several facets of an individual's existence, encompassing mental well-being, accomplishments, interpersonal engagements, and coping abilities-->Evidence is needed for this statement.

**Response:** Thank you for your correction. We have corrected and replaced it following your suggestions.

(Page 3; paragraph 1)

In summary, self-esteem is a pivotal psychological construct [6] that controls several facets of an individual's existence, encompassing mental well-being, accomplishments, interpersonal engagements, and coping abilities [3, 4].

2. There exists a reciprocal relationship between self-esteem and mental illnesses. A previous study found that self-esteem plays a pivotal role in developing diverse mental illnesses and social problems encompassing a range of internalizing issues, such as depression, suicidal tendencies, eating disorders, and anxiety, as well as externalizing problems, including violence and substance abuse -->Evidence is needed for this statement. A visual representation or diagram displaying the relationship between constructs/variables would be interesting for the reader.

**Response:** Thank you for your correction and suggestion. We have corrected and replaced it following your suggestions.

(Page 3; paragraph 2)

There is a reciprocal relationship between self-esteem and mental illnesses [7].

3. Researchers often use the RSES to measure self-esteem in the clinical population [15]--> Please add the characteristics of the population, diagnosis...

**Response:** Thank you for your correction and suggestion. We have corrected and replaced it following your suggestions.

(Page 3; paragraph 3)

Researchers often use the RSES to measure self-esteem in the clinical population, such as eating disorders [18], anxiety, depression [7], attention and emotional disorder [19], schizophrenia and bipolar disorder [20]. Other studies have tested the RSES in specific people, such as ex-prisoners [21], drug users [22], and single mothers [23].

4. General Overview: The repetition of some ideas and the lack of a smooth transition between paragraphs make it difficult to read. Ensure that the citations are correctly referenced and that there is coherence in the bibliography throughout the text. Some key statements lack specific citations or references, which compromises the credibility of the text. Through a brief search, I find a large number of validations, such as those in Spanish, which the authors have not included in the relevant section of the introduction. An exposition of the structural differences and psychometric properties of the tool in the target population of the study should be made, in this case, in patients with schizophrenia.

**Response:** Thank you for your correction and suggestion. We have corrected and replaced it following your suggestions, including the substitution of multiple sentences in the introduction to make it easier for readers to fully understand our idea. (See page 3-4; paragraph 1-5)

(Page 3-4; paragraph 3)

The RSES has been translated and adapted into a number of different languages, including German [24], Dutch [25], Estonian [26], French [27], Portuguese [28], Spanish [29], Japanese [17], and Thai [14]; thus, making it applicable to participants from diverse samples or populations.

METHODS:

5. Instruments-->Remove the numbering from the categorical variables. Is the instruments section appropriate to discuss the known evidence of internal consistency?

**Response:** Thank you for your correction and suggestion. We have corrected and replaced it following your suggestions. This section aims to elucidate the rationale for the utilization of this questionnaire. It will accomplish this by providing information about the questionnaire's developer, the scale employed, its interpretation, and the questionnaire's validity and reliability evidence, as established by prior research.

(Page 5)

The remaining variables as categorical variables, namely gender (male; female), marital status (single; married; divorced, or widowed), employment status (employed; unemployed), source of income (personal income; family support; personal and family support), education (elementary; junior; high school; university/ college), previous hospitalization (yes; no), and onset of illness (<1 year; 1-5 years; >5 years).

6. Translation procedure-->Include an explanatory diagram/figure of the process.

**Response:** Thank you for your correction and suggestion. We have provided the flow diagram of the translation procedure (see: Figure 1)

(Figure 1)

**Stage 6: Full psychometric testing**

The full Indonesian version of the RSES scale was administered to the study sample

**Stage 1: Forward translation**

Two translators, a psychiatrist and professional translator [T1 & T2].
Forward translation into the target language (Indonesia)

**Stage 2: Synthesis I**

Compared the two translations by T1 & T2
Created a new draft [TL1]

**Stage 3: Back translation**

**A** draft [TL1] was back translated by two independent translators [T3 & T4]
A psychologist and professional translator.

**Stage 4: Synthesis II**

Compared the translation from stage 3.
Created a new draft [TL2]

**Stage 5: Pre-testing**

Invite 30 volunteers for format clarity of the questionnaire [instruction, items and response format]
Expert panel [a psychiatrist and psychologist]

7. Statistical Analysis--> Please remove the sub-sections and write in a simpler manner.

**Response:** Thank you for your correction and suggestion. We have corrected and replaced it following your suggestions. (Page 7-8).

8. Validity evidence--> What estimator have you used for the CFA? ULS, WLSMV?

**Response:** Thank you for your correction and suggestion. Unweighted Least Squares (ULS) was used in our analysis, since our data meet the assumption as a continuous and normally distributed.

9. General Overview: Please present structural validity section before reliability and internal consistency. I refer you to COSMIN. The data on internal consistency cannot be interpreted before data on structural validity.

**Response:** Thank you for your correction and suggestion. We have corrected and replaced it following your suggestions, as mentioned based on the COSMIN.

RESULTS:

10. General Overview: Please provide the evidence for structural validity before internal consistency and reliability. According to COSMIN (reference provided in the first review), internal consistency and reliability cannot be interpreted without first having evidence of structural validity.

**Response:** Thank you for your correction and suggestion. We have corrected and replaced it following your suggestions.

(Page 9)

*Structural validity.*

The goodness of indices for all alternative models is shown in Table 3. Considering the single-factor or uni-dimensional model (M1a), the overall fit criteria were inadequate (See Figure 2). However, after some modification indices (See Figure 3), all fit criteria were significantly adequate (M1b). The adequacy of all fit criteria remained satisfactory when the two-factor model (M2) and hierarchical model (M3) were applied (See Figures 4 and 5). The AVE values were 0.69 and 0.68, and the square roots of the AVE were 0.83 and 0.82, indicating that each measured variable was significant (Table 4).

*Internal consistency and reliability evidence.*

The RSES had an alpha coefficient of 0.75, according to Cronbach's method. The results of Cronbach's alpha, which measures internal consistency, came in at 0.89 and 0.88 for each subscale (factor), indicating acceptable internal consistency. As presented in Table 4, the CR was calculated for positive and negative factors, and the values were 0.92 and 0.91, respectively. Test-retest reliability exhibited satisfactory results, with an ICC between 0.87 and 0.93 (Table 4).

DISCUSSION:

11. General Overview: The discussion still requires further development. Currently, the flow of information is disorganized. I also encourage the authors to delve into the results they have obtained and provide an explanation and reasoning behind their findings. They do not discuss the advantages of obtaining a single factor or the disadvantages of not obtaining it... I'm also unsure whether the studies they are comparing with have clinical or non-clinical samples. There is an anthropomorphic language that should be removed. The writing needs to be revised as it still does not adhere to AERA, APA, NMCE, COSMIN standards.

**Response:** Thank you for your correction and suggestion. We have corrected and replaced it following your suggestions. Such as; *construct validity* was replaced specifically as *structural validity*; and *reliability* was replaced and divided as *internal consistency and reliability*. The details are on pages 9-11.

Next, I copy fragments of the discussion that bother me when reading:

"Surprisingly", "can be conceptualized as comprising two distinct constructs", "In short, the evidence from our study shows that the RSES scale construct fits well", Our sample size was adequate to perform factor analysis", "Our results were consistent with a previous study by [54], conducted in", "individuals with severe mental illnesses, not specific only to patients with schizophrenia and reported strong internal consistency".......

**Response:** Thank you for your correction and suggestion. We have also improved the discussion section to make it easier for readers to understand what we mean in our study. The details are in the discussion section (pages 9-11). Furthermore, this manuscript was edited by Wallace Academic Editing Company to help us in providing writing corrections to our manuscript so that it complies with journal standards.

**REVIEWER #3 COMMENTS:**

1. Authors have successfully adressed all of my concerns. The manuscript can be considered for its publication now.

**Response:** We express our gratitude for your kind attention**.**
