# Supplementary material for: Structural validity of the Rosenberg self-esteem scale in patients with schizophrenia in Indonesia
Source: PLoS One. 2024 May 10;19(5):e0300184. doi: 10.1371/journal.pone.0300184 (PMC11086833; doi:10.1371/journal.pone.0300184)
Supplement: Attachment — Submitted filename: Response to editor and reviewer.docx [file pone.0300184.s005.docx]

Dear Editor and Reviewer

Manuscript ID:

Manuscript Title: “Structural Validity of the Rosenberg Self-Esteem Scale in Patients with Schizophrenia in Indonesia”.

We sincerely thank the editor and all reviewers for their valuable suggestions and for allowing us to revise our manuscript entitled “Structural Validity of the Rosenberg Self-Esteem Scale in Patients with Schizophrenia in Indonesia”. We have incorporated all the suggested changes into the manuscript and have highlighted the revised sections. At this moment, our responses and revisions are based on the editor and reviewer’s comments.

--------------------------------------------------------------------------------------------------

**EDITORIAL COMMENTS:**

Please review your reference list to ensure that it is complete and correct. If you have cited papers that have been retracted, please include the rationale for doing so in the manuscript text, or remove these references and replace them with relevant current references.

**Response:** Thank you for your suggestion. We have verified the references in our manuscript and confirmed that the cited papers are still relevant.

**REVIEWER #2 COMMENTS:**

In the attached document I make some recommendations to improve the scientific quality of the article. The authors argued in previous reviews that the use of ordinal alpha was inadequate, using a critique by Chalmers. I provide a reference for you to reflect on and assess whether it would be relevant to modify the internal consistency estimator.

Zumbo, B. D., & Kroc, E. (2019). A Measurement Is a Choice and Stevens' Scales of Measurement Do Not Help Make It: A Response to Chalmers. Educational and psychological measurement, 79(6), 1184–1197. <https://doi.org/10.1177/0013164419844305>

**Response:** Thank you for your correction. We have corrected and replaced it following your suggestions. As a consideration, both Cronbach’s and Ordinal alpha methods produce similar results of reliability alpha.

(Page 7)

This study utilized SPSS and AMOS version 23.0 software (IBM; Armonk, New York, USA) and R Studio version 4.3.2 (R Foundation, Vienna, Austria).

(Page 8)

Ordinal coefficient alpha is considered a viable alternative coefficient alpha to calculate a reliability estimate using the Likert response data [59, 60].

**(**Page 9)

The overall score of the RSES has an alpha coefficient of 0.75, as determined by the ordinal alpha approach. The ordinal alpha results for each subscale were 0.89 and 0.88, demonstrating acceptable internal consistency.
